# Host origin of plastid solute transporters in the first photosynthetic eukaryotes

**DOI:** 10.1186/gb-2007-8-10-r212

**Published:** 2007-10-05

**Authors:** Heather M Tyra, Marc Linka, Andreas PM Weber, Debashish Bhattacharya

**Affiliations:** 1 Department of Biological Sciences and Roy J Carver Center for Comparative Genomics, 446 Biology Building, University of Iowa, Iowa City, IA 52242-1324, USA; 2 Department of Plant Biology, S-336 Plant Biology Building, Michigan State University, East Lansing, Michigan 48824-1312, USA; 3 Current address: Institute for Plant Biochemistry, Heinrich-Heine-University, Gebäude 26.03.01, Universitätsstrasse 1, D-40225 Düsseldorf, Germany

## Abstract

**Background:**

It is generally accepted that a single primary endosymbiosis in the Plantae (red, green (including land plants), and glaucophyte algae) common ancestor gave rise to the ancestral photosynthetic organelle (plastid). Plastid establishment necessitated many steps, including the transfer and activation of endosymbiont genes that were relocated to the nuclear genome of the 'host' followed by import of the encoded proteins into the organelle. These innovations are, however, highly complex and could not have driven the initial formation of the endosymbiosis. We postulate that the re-targeting of existing host solute transporters to the plastid fore-runner was critical for the early success of the primary endosymbiosis, allowing the host to harvest endosymbiont primary production.

**Results:**

We tested this model of transporter evolution by conducting a comprehensive analysis of the plastid permeome in *Arabidopsis thaliana*. Of 137 well-annotated transporter proteins that were initially considered, 83 that are broadly distributed in Plantae were submitted to phylogenetic analysis. Consistent with our hypothesis, we find that 58% of *Arabidopsis *transporters, including all carbohydrate transporters, are of host origin, whereas only 12% arose from the cyanobacterial endosymbiont. Four transporter genes are derived from a *Chlamydia*-like source, suggesting that establishment of the primary plastid likely involved contributions from at least two prokaryotic sources.

**Conclusion:**

Our results indicate that the existing plastid solute transport system shared by Plantae is derived primarily from host genes. Important contributions also came from the cyanobacterial endosymbiont and *Chlamydia*-like bacteria likely co-resident in the first algae.

## Abstract 2

Analysis of plastid transporter proteins in Arabidopsis suggests a host origin and provides new insights into plastid evolution.

## Background

Plastids in eukaryotes that contain chlorophyll are capable of carrying out photosynthesis, a process that converts light energy, carbon dioxide, and water into organic compounds. The evolutionary history of this organelle unfolded over a billion years ago when a previously non-photosynthetic protist engulfed and maintained a free-living cyanobacterium in its cytoplasm [1]. It is hard to over-state the importance of this ancient and extraordinarily rare primary endosymbiosis because plastids allowed the evolution of algae and the plants that form the base of the food chain for many ecosystems on Earth. Current data suggest that the primary endosymbiosis occurred once in the common ancestor of the red, green (including land plants), and glaucophyte algae, the Plantae [2-4], with the original plastid and the nuclear-encoded machinery for running the organelle spreading in subsequent cell captures to other branches of the eukaryotic tree [5-7]. The only other known case of a potential *bona fide *cyanobacterial primary endosymbiosis occurred relatively recently in the thecate amoeba *Paulinella chromatophora *[8,9].

The gradualist view of evolution through mutation-selection suggests that it would have taken millions of years for the captured prokaryote to become fully integrated into the 'host' eukaryote, ultimately becoming the site not only for carbon fixation but also for other complex functions, such as lipid, isoprenoid, and amino acid biosynthesis [10]. These processes were associated with the migration of much of the cyanobacterial genome to the host nucleus and development of the complex protein import system that are key shared features among all canonical plastids [3,11,12]. A remarkable exception to the view that endosymbiosis was a gradual process of integration is offered by the katablepharid protist 'Hatena', which undergoes large-scale morphological changes following the engulfment of a green alga [13].

Regardless of whether the ancient primary endosymbiosis fostered an accelerated rate of morphological evolution in the Plantae ancestor or whether general cell morphology was unchanged as in the *Paulinella *example [14], one thing is clear - in the absence of rapid benefits to the host it is unlikely that the endosymbiosis would long have been sustained. Given the need for short-term survival, a key feature of early success for the endosymbiosis must have been the integration of the metabolism of the two cells. The key to this process would have been solute transporters that regulate the flux of metabolites (for example, ATP, phosphate, sugars and sugar phosphates, metal ions, and other important ions) across the organelle membranes. Controlled exchange in response to environmental factors such as changes in light intensity and trace metal availability [15-17] is decisive because the unregulated flux of metabolites would have had detrimental effects and, thereby, lowered the evolutionary fitness of the endosymbiosis. A complex system of solute transporters is in place today in extant plastids that provides the link between this organelle and the surrounding cytosol [18-20]. Here we focus on the evolutionary history of these plastid metabolite transporters to infer early events in plastid evolution.

We make two assumptions in this study. First, a system of metabolite transporters was a critical and early development in plastid evolution to supply the endosymbiont with essential nutrients and to enable the host to reap immediate benefit from photosynthetic primary production. It is unclear why the cyanobacterium that was destined to become the plastid escaped digestion in the host but this scenario has also played out in 'Hatena' and in *Paulinella*. Second, whereas the genome of the previously free-living cyanobacterium encoded all the transport systems required for the uptake of essential inorganic nutrients, it most likely did not harbor genes encoding transporters for the export of organic solutes to the host - this would have served no obvious pre-existing purpose in the prokaryote. Precisely how the plastid solute transport system was established is unknown. One possible model involves a primarily cyanobacterial origin, in which the plastid continued to utilize its own original cyanobacterial solute transporters with their evolution over time into proteins that perform most or all currently known plastid permeome functions. An alternative model involves a host-driven solute transport system, likely derived from the vacuolar envelope that initially surrounded the endosymbiont after its engulfment [3]. And finally, both of the new partners could have contributed proteins equally to this machinery, resulting in a chimeric system composed of the most beneficial combination possible of prokaryotic and eukaryotic transporters. To determine which of these competing hypotheses best explains plastid transporter evolution, we undertook an initial bioinformatics analysis of 137 *Arabidopsis thaliana *solute transporters and then a detailed phylogenetic analysis of a subset of 83 conserved proteins that included available data from other Plantae. The *Arabidopsis *transporters are either predicted or have been shown to be chloroplast targeted and are ideal for tracking plastid permeome evolution. Using these data we demonstrate that over one-half of Plantae plastid targeted transporters are putatively of host origin whereas less than a quarter arose from the cyanobacterial endosymbiont. This suggests that the lasting contribution to the Plantae host-endosymbiont relationship with regard to the plastid solute transport system was made primarily by host genes. We also find evidence for the origin of four transporter genes or gene families from a *Chlamydia*-like source. This latter result raises the possibility that establishment of the ancient primary plastid may have involved contributions from at least two prokaryotic sources, perhaps explaining its singular nature. This hypothesis received substantial support from the recent finding of at least 21 genes of *Chlamydia*-like origin in the nuclear genome of the extremophilic red alga *Cyanidioschyzon merolae *[21].

## Results and discussions

### Distribution of transporters within Plantae

**Figure 1:**
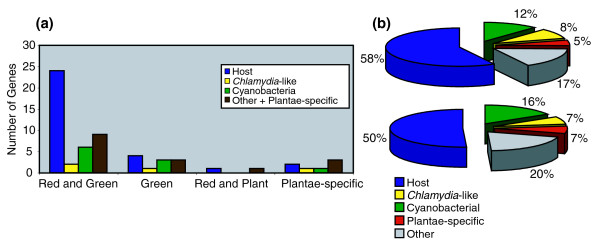
Origin of plastid targeted solute transporters in Plantae. **(a) **Gene distribution among Plantae and gene origin for 63 distinct transporters considered in this study. **(b) **Summary pie-charts showing the origin of all the 83 transporters (top chart) and the 63 distinct genes (lower chart) considered in this study.

Phylogenetic analysis of the best-annotated transporter data that are currently available from *Arabidopsis *was used to identify and putatively annotate homologs from other Plantae. Of 137 transporter proteins that were initially considered, BLAST and phylogenetic analyses and manual curation of recently available data led to the identification of 83 proteins that were of sufficient conservation and broad distribution among Plantae to be used for further analyses. Each of these 83 proteins that included gene families (that is, representing 63 distinct, ancestral genes; Table 1) was used as input in BLAST and PHYML bootstrap analyses to infer the trees. This approach identified 41 proteins that are present in both red and green algae (including land plants) and, therefore, were likely found in the Plantae ancestor (glaucophyte homologs were found for some of these genes; for example, ADP/ATP translocase, hypothetical protein At3g45890). Eleven proteins were restricted to green algae and land plants, seven were plant-specific, and two were limited to red algae and land plants. The distribution of these proteins with respect to their putative origin in Plantae is shown in Figure 1a. Given the lack of evidence for widespread horizontal gene transfer in extant Plantae, which most likely lost the capacity for phagotrophy early in its evolution [4,22], we postulate that the patchy distribution for many plastid targeted transporters primarily reflects differential gene loss over the greater than one billion years that has passed since the primary endosymbiosis [1]. Under this interpretation, the large set of shared transporters among Plantae lineages provides resounding support for the monophyly of this supergroup [23].

**Table 1:** *Arabidopsis *solute transporters

***Host***	
At1g05580	Cation/hydrogen exchanger
At1g54320	Ligand-effect modulator 3 (LEM3) family
At1g59870	ABC transporter
At1g61800	Glucose-6-phosphate/phosphate translocator 2 (GPT2)
At1g64150	Expressed protein
At1g66950	ABC transporter
At1g70610	Transporter associated with antigen processing protein 1 (AtTAP1)
At1g79450	Ligand-effect modulator 3 (LEM3) family
At2g04620	Cation efflux family protein
At2g13100	Glycerol-3-phosphate transporter
At2g27810	Xanthine/uracil permease
At2g28070	ABC transporter
At2g29650	Na+-dependent inorganic phosphate cotransporter
At2g38060	Na+-dependent inorganic phosphate cotransporter
At2g38330	Multi antimicrobial extrusion (MATE Efflux) protein
At2g40420	Amino acid transporter
At3g01550	Phosphoenolpyruvate/phosphate translocator 2 (PPT2)
At3g12740	Ligand-effect modulator 3 LEM3 family
At3g17690	Cyclic nucleotide-binding transporter 2
At3g17700	Cyclic nucleotide-binding transporter 1
At3g45890	Expressed protein
At3g52310	ABC transporter
At4g00370	Anion transporter 2 (ANTR2)
At4g13590	Expressed protein
At4g17340	Major intrinsic family protein
At4g25750	ABC transporter
At4g32400	Adenine nucleotide uniporter
At4g32650	*Arabidopsis thaliana *K^+ ^rectifying channel 1 (ATKC1)
At4g38380	Multi antimicrobial extrusion (MATE Efflux) protein
At4g39460	S-adenosylmethionine carrier 2 (SAMT)
At5g04770	Amino acid permease
At5g05630	Amino acid permease
At5g13550	Sulfate transporter
At5g14040	Mitochondrial phosphate transporter
At5g16150	Hexose transporter
At5g17630	Glucose-6-phosphate transporter 1 (XPT)
At5g19410	ABC transporter (White)
At5g19600	Sulfate transporter
At5g22830	CorA-like magnesium transporter
At5g26820	Ferroportin-related protein
At5g33320	Phosphoenolpyruvate/phosphate translocator (PPT1)
At5g42130	Mitochondrial substrate carrier family
At5g45450	Iron transporter-related
At5g46110	Triose phosphate translocator (TPT)
At5g52860	ABC transporter (White)
At5g54800	Glucose-6-phosphate/phosphate transporter 1(GPT1)
At5g59250	Sugar transporter
***Cyanobacterial***	
At1g04570	Integral membrane family protein
At1g08640	Expressed protein
At1g19800	Trigalactosyldiacylglycerol 1, TGD1
At1g78620	Integral membrane family protein
At2g32040	Folate monoglutamate transporter, FT
At3g51140	Expressed protein
At3g60590	Expressed protein
At4g33520	Metal-transporting P-type ATPase (PAA1)
At5g12470	Expressed protein
At5g64940	ABC1-family protein
***Chlamydia-like***	
At1g15500	Adenine nucleotide translocase 2 (AtNTT2)
At1g80300	Adenine nucleotide translocase 1 (AtNTT1)
At3g26570	Low affinity phosphate transporter (PHT2;1)
At4g37270	Heavy metal ATPase HMA1
At5g12860	Dicarboxylate translocator 1 (DiT1)
At5g64280	Dicarboxylate translocator 2.2 (DiT2.2)
At5g64290	Dicarboxylate translocator 2.1 (DiT2.1)
***Other***	
At1g01790	Potassium transporter
At1g32080	Membrane protein
At1g44920	Expressed protein
At1g54350	ABC transporter
At1g78560	Bile acid:sodium symporter
At2g02590	Expressed protein | (Putative small multi-drug export)
At2g21340	Enhanced disease susceptibility protein
At2g26900	Bile acid:sodium symporter
At3g25410	Bile acid:sodium symporter
At4g30580	1-Acylglycerol-3-phosphate O-acyltransferase (ATS2)
At5g03555	Cytosine/purines, uracil, thiamine, allantoin family permease
At5g13720	Expressed protein
At5g52540	Expressed protein
At5g62720	Integral membrane HPP family protein
***Plantae-specific***	
At2g38550	Expressed protein
At3g57280	Expressed protein
At5g17520	Maltose transporter (MEX1)
At5g24690	Expressed protein

List of *Arabidopsis thaliana *chloroplast solute transporters analyzed in this study and their putative evolutionary origins.

### Most proteins of the plastid envelope permeome are host-derived

**Figure 2:**
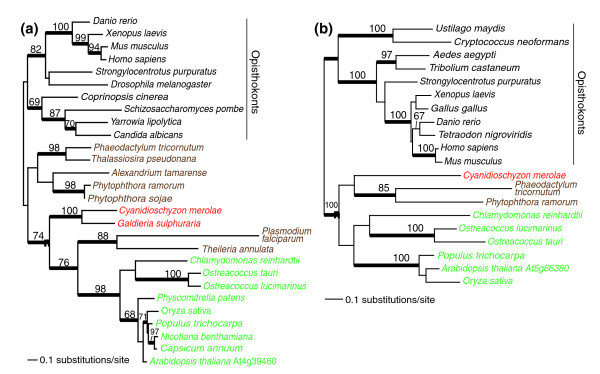
Plastid targeted solute transporters of putative 'Host' origin in Plantae. These are RAxML trees with the numbers above the branches inferred from a RAxML bootstrap analysis and the thick branches showing significant (*P *> 0.95) support from a Bayesian phylogenetic inference. Only bootstrap values ≥ 60% are shown. Branch lengths are proportional to the number of substitutions per site (see scale bars). The filled magenta circle shows the node that unites the Plantae taxa within the eukaryotic domain. The different algal groups are shown in different text colors: red for red algae, green for green algae and land plants, and brown for chromalveolates. The inclusion of chromalveolates within the Plantae is believed to reflect horizontal or endosymbiotic gene transfer events (for example, [50]). The two transporters are: **(a) **SAMT, S-adenosylmethionine carrier 1 protein; and **(b) **AtFOLT1, *Arabidopsis thaliana *folate transporter 1. The name of the *A. thaliana *solute transporter used for the query is indicated for both trees shown in this figure.

Analysis of the phylogenetic data supports the notion that the host drove the integration of plastid and host metabolism. We find that the majority (58%, when considering all 83 genes; Figure 1b and Table 1) of the plastid solute transporters were most likely derived from existing host membrane proteins (see Figure S1 in Additional data file 1 for all trees). These 48 proteins are diverse in nature, including several ABC transporters, nucleotide and amino acid permeases, sulfate, potassium, magnesium, and iron transporters, and cation efflux proteins (see Figure 2 for S-adenosylmethionine carrier 1 (SAMT) and *Arabidopsis thaliana *folate transporter 1 (AtFOLT1) trees). Of particular interest is the finding that in addition to the members of the nucleotide-sugar/triose phosphate translocator gene family previously reported to be of host origin [3], all other carbohydrate transporters included in our analysis were derived from existing host proteins. This result strongly suggests that the host utilized existing eukaryotic transport proteins pre-adapted to this function to 'tap' into the photosynthates produced by the captured cyanobacterium. In addition, the Plantae host also provided transporters to facilitate the movement of valuable nutrients such as magnesium, potassium, iron, and phosphate into the captured prokaryote. The replacement of pre-existing cyanobacterial anion and cation transporters with host derived proteins again suggests that there was strong selection to rapidly establish control over and utilize the endosymbiont. This process was most likely accomplished by using transporters derived from the host vacuolar envelope [3].

### The cyanobacterial contribution

**Figure 3:**
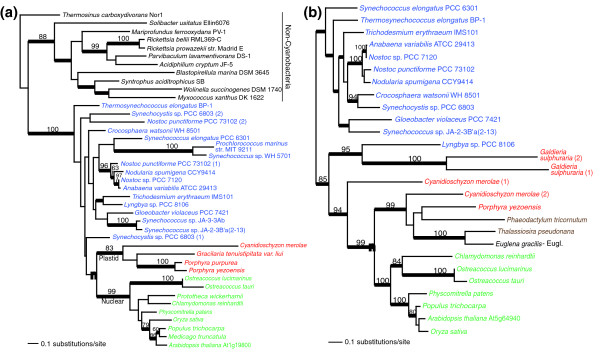
Plastid targeted solute transporters of putative 'Cyanobacterial' (that is, plastid endosymbiont) origin in Plantae. For details of tree building see Figure 2. The filled magenta circle shows the node that unites the Plantae taxa as sister to cyanobacteria. The different photosynthetic groups are shown in different text colors: blue for cyanobacteria, red for red algae, green for green algae and land plants, and brown for chromalveolates. The inclusion of chromalveolates or Euglenozoa (Eugl.) within the Plantae is believed to reflect horizontal or endosymbiotic gene transfer events (for example, [50]). The two transporters are: **(a) **TGD1, trigalactosyldiacylglycerol 1, lipid transporter; and **(b) **ABC1-family transporter protein. The name of the *A. thaliana *solute transporter used for the query is indicated for both trees shown in this figure.

The cyanobacterial endosymbiont putatively contributed ten solute transporters to the plastid transport system (Table 1, Figure S2 in Additional data file 1). These proteins include trigalactosyldiacylglycerol 1 (TGD1; Figure 3a), which is required for integrating the prokaryotic (that is, cyanobacterial) with the eukaryotic (that is, endoplasmic reticulum) pathway for lipid biosynthesis [24-26], the metal-transporting P-type ATPase PAA1 [27,28], and a transporter required for folate/biopterin biosynthesis [29]. The remaining seven proteins of unknown function that are localized to the chloroplast inner membrane were included in the cyanobacterial group. Whereas the predicted secondary structure of most of these proteins indicates they represent transporters (that is, they contain at least four transmembrane domains that are connected by short loops), some, such as the ABC1-family protein At5g64940 (Figure 3b) contain only one or two predicted transmembrane domains and may thus have functions other than metabolite transport. It is also intriguing that with the exception of the PAA1 copper transporter the only cyanobacterial transport proteins apparently retained by *Arabidopsis *are those for which the host lacked a suitable replacement. For example, the initial steps of folic acid biosynthesis in plants are confined to the chloroplast; the final steps are localized in the cytosol and in mitochondria [30-32]. Plastids thus depend on an external folate supply and require an uptake system for this important metabolite. Interestingly, redundant systems for folate uptake exist in *Arabidopsis *chloroplasts, consisting of the cyanobacterial-derived folate transporter FT [29] and the host-derived transporter AtFOLT1 [33].

### '*Chlamydia*-like' transporters

**Figure 4:**
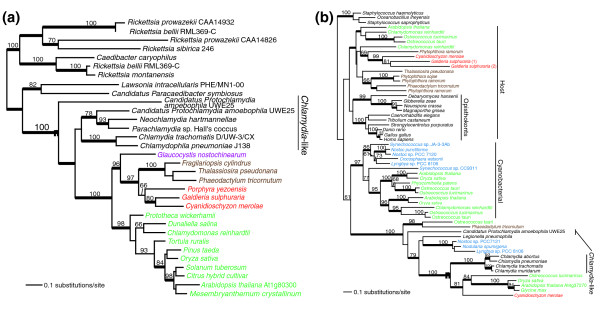
Plastid targeted solute transporters of putative '*Chlamydia*-like' origin in Plantae. For details of tree building see Figure 2. The filled magenta circle shows the node that unites chlamydial taxa with plastid targeted Plantae transporters. The different photosynthetic groups are shown in different text colors: blue for cyanobacteria, red for red algae, green for green algae and land plants, magenta for glaucophytes, and brown for chromalveolates. The inclusion of chromalveolates within the Plantae is believed to reflect horizontal or endosymbiotic gene transfer events (for example, [50]). The two transporters are: **(a) **ADP/ATP translocater; and **(b) **heavy metal ATPase (HMA1) copper transporter. The name of the *A. thaliana *solute transporter used for the query is indicated for both trees shown in this figure.

In addition to the host and cyanobacteria, a third significant contributor to the Plantae plastid solute transport system is the Chlamydiae. A surprisingly high number (four) of plastid envelope membrane transporters have been contributed by these prokaryotes. The presence of plant-like genes in *Chlamydia *has been noted in the past, sparking debate over whether their presence indicated a transfer from the ancestral plant to *Chlamydia*, an evolutionary relationship between cyanobacteria and *Chlamydia*, or a horizontal gene transfer (HGT) from a chlamydial parasite to the plant ancestor [34-36]. Phylogenetic analysis of plastid, Chlamydiae, and Rickettsiae ADP/ATP translocases [36] supports an ancient *Chlamydia*-to-Plantae direction of transfer. This explanation for the origin of the ADP/ATP translocase gene (and other *Chlamydial*-like genes) in Plantae was strongly supported by the phylogenomic analysis of Huang and Gogarten [21]. We found a monophyletic relationship between the AtNTT1 and AtNTT2 (the *Arabidopsis *plastid ADP/ATP translocases) and Chlamydiae ADP/ATP translocases (Figure 4a) [37,38]. In addition, the copper transporter heavy metal ATPase 1 (HMA1; Figure 4b), the dicarboxylate translocators (DiTs) DiT1, DiT2.1, and DiT2.2, and the low affinity phosphate transporter PHT2;1 (see Figure S3 in Additional data file 1 and [21]) apparently has a chlamydial origin in Plantae. All of these trees provide bootstrap (except for the DiT tree) support for the monophyly of the '*Chlamydia*-like' and plastid transporters. In the case of HMA1 there are two ancient paralogs in plants, one of cyanobacterial likely endosymbiotic origin and one from a *Chlamydia*-like source that is shared with red and green algae. The DiTs [39] are present only in green algae, plants, and bacteria (that is, not in red algae). Whereas genomic data for glaucophytes are not yet available, transport experiments using isolated *Cyanophora *cyanelles showed that this glaucophyte uses a transport system for glutamine and 2-oxoglutarate that is distinct from green plant DiTs [40]. Taken together, these data indicate that '*Chlamydia*-like' dicarboxylate translocators have likely been lost from red algae and glaucophytes. An alternative explanation is that the gene was acquired by the green lineage after the split of Chlorophyta and Rhodophyta. A DiT2 gene was also found in the dinoflagellates *Amphidinium carterae *and *Heterocapsa triquetra*, which likely originated from an independent HGT. Several 'green' genes have been found in dinoflagellates and other chromalveolates that could have either originated from multiple independent HGTs or an ancient green algal endosymbiosis (for discussion, see [41]).

In summary, it is surprising that bacteria not putatively involved in the endosymbiosis contributed 8% of the transporters that we have identified. When one considers the functions of these transporters, the chlamydial contribution becomes more important. HMA1 increases copper and/or zinc transport into the plastid under conditions of high light, facilitating the production of copper/zinc superoxide dismutase (CuZnSOD), which protects the plant from superoxide radicals produced under high light conditions [42,43]. PHT2;1, a phosphate transporter, controls phosphate allocation under conditions of phosphate-starvation [44]. The DiT transporters are involved in assimilating nitrogen and recovering carbon lost to photorespiration, a process that is initiated by the oxygenation reaction of Rubisco that primarily occurs under conditions when a high O_2_:CO_2 _ratio is present in the vicinity of Rubisco. Mutants lacking these transporters are unable to survive in ambient CO_2 _concentrations [17,45,46]. Finally, the AtNTT1 and AtNTT2 transporters are required for ATP import into the plastid during the dark (that is, in the absence of photosynthetic ATP production), particularly during lipid and chlorophyll biosynthesis. Although AtNTT2 mutants are still capable of producing lipids, indicating that the plastid has an alternative method for generating the ATP required for lipid biosynthesis, the production is significantly reduced and mutant plants have a sharply reduced growth rate [16]. *Arabidopsis *mutants deficient in both AtNTT1 and AtNTT2 develop necrotic lesions when grown under short days, accumulate H_2_O_2_, and, strikingly, show constitutive expression of CuZnSOD2 and ascorbate peroxidase [47]. The phenotype of the mutant was linked to reduced magnesium chelatase activity and it was concluded that ATP import into plastids in the dark is required for chlorophyll biosynthesis and for preventing photooxidative damage [47]. The import of ATP into plastids in the dark is thus clearly a case in which the endosymbiont benefits from host metabolism. The ancient origin of these transporters in the tree of photosynthetic eukaryotes (Figure 4a) is indicative of an essential role of this uptake system in the formation of the endosymbiosis. With the exception of the DiT translocators, each of these transporters appear to perform somewhat redundant functions (that is, copper and phosphate transport) but in a way that permits the plant to adapt to stresses involved in life on the land (that is, high light and O_2 _levels or low phosphate availability). This may explain why the genes encoding these four plastid transporters have been retained in the *Arabidopsis *genome.

How the '*Chlamydia*-like' genes entered into the Plantae ancestor is unclear but it is possible that both the cyanobacterial endosymbiont and chlamydial parasites may have co-existed in the cell. Many environmental *Chlamydia *are known today that are broadly distributed in animals and protists [48]. The co-existence of these two distinct prokaryotes may have provided the genetic 'toolkit' to make permanent the endosymbiosis with gene transfer from each cell providing essential functions for endosymbiont utilization. An alternative explanation is that the cyanobacterial endosymbiont was itself highly chimeric (that is, the 'fluid chromosome model') [49] and contained genes of chlamydial origin that had been gathered through HGT. Although possible, this scenario seems less plausible because it invokes, for example, the presence of an ADP/ATP translocator (a gene typical for 'energy parasites' such as Rickettsiae) in the genome of an oxygenic photosynthetic cell that is unlikely to encounter high concentrations of ATP in the surrounding environment; that is, it is absent from all studied cyanobacteria. Additional discussion of these issues can be found in Huang and Gogarten [21].

### 'Other' and 'Plantae-specific' transporters

**Figure 5:**
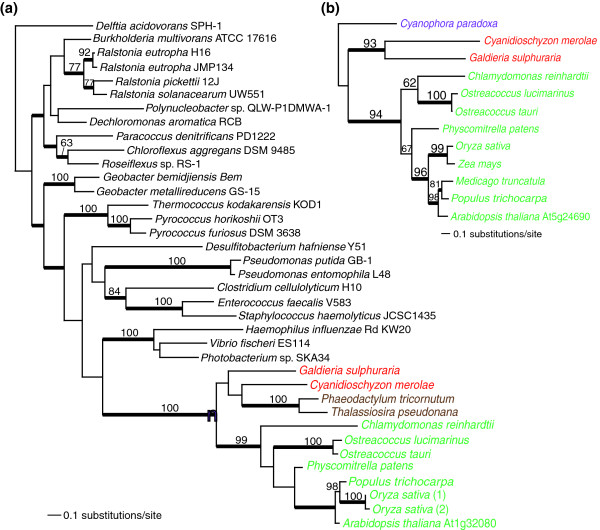
Plastid targeted solute transporters of 'Other' or 'Plantae-specific' origin in Plantae. For details of tree building see Figure 2. The filled magenta circle shows the node that unites the Plantae taxa. The different algal groups are shown in different text colors: red for red algae, green for green algae and land plants, magenta for glaucophytes, and brown for chromalveolates. The inclusion of chromalveolates within the Plantae is believed to reflect horizontal or endosymbiotic gene transfer events (for example, [50]). The different transporters are: **(a) **transporter in the 'Other' category: putative membrane protein; and **(b) **transporter in the '*Plantae*-specific' category: hypothetical expressed protein. The name of the *A. thaliana *solute transporter used for the query is indicated for both trees shown in this figure.

We were unable to conclusively determine the origin of 18 transport proteins. Fourteen of these data sets resulted in PHYML trees in which the Plantae transporters were rooted within prokaryotes but without bootstrap support for a specific affiliation. An excellent example is provided by At1g32080 (Figure 5a), which is a putative membrane protein conserved among Plantae, chromalveolates, and a diverse set of Eubacteria and Archaea (that is, the *Thermococcus *and *Pyrococcus *clade). Although the prokaryotic source of this gene in Plantae is unclear with the available data, the eukaryotic clade is clearly monophyletic, which is consistent with a single gene origin in the Plantae ancestor and, thereafter, transfer to chromalveolates (for example, diatoms in this tree) via secondary endosymbiotic gene transfer [50]. The unresolved provenance of At1g32080 and the 'Other' set of transporters in Plantae can be explained by pervasive HGT followed by full or partial gene replacement or differential gene loss among prokaryotes that has erased the ancient phylogenetic signal. Alternatively, these results may indicate erratic rates of sequence divergence that make it impossible to model protein evolution for these sequences. Given the growing evidence, however, for recurring HGT among bacteria [51], it is likely that genes in the 'Other' category have reticulate evolutionary histories. In this regard it is noteworthy that the likely frequent HGTs seen in Figure 5a among prokaryotes and other genes in the 'Other' category contrasts starkly with the apparent single origin and vertical inheritance in Plantae (for example, At4g30580, At5g13720, At5g52540, At5g62720; Figure S4 in Additional data file 1). This result suggests a clear difference in rates of HGT for these genes with elevated rates in prokaryotes relative to eukaryotes.

Of the remaining transporters, four fell in the 'Plantae-specific' category because they lacked identifiable homologs outside of this supergroup and may simply be too divergent to determine their origin. This includes At5g24690 (Figure 5b, a hypothetical expressed protein) and the plastidic maltose exporter MEX1. The latter is required for export of maltose resulting from starch breakdown from plastids at night in green plants (Figure S5 in Additional data file 1). Storage of starch inside the chloroplast is exclusively found in the green linage. Therefore, MEX1 has likely co-evolved with plastid-based starch biosynthesis and breakdown since it can be detected only in members of the Viridiplantae with one gene found in the dinoflagellate *Karlodinium micrum*, which, as described above for Dit2, likely has resulted from a HGT.

## Conclusion

Here we determined the phylogeny of 83 *Arabidopsis *plastid solute transporters to determine whether they are of endosymbiotic origin from the captured cyanobacterium, of host origin, or of a 'mixed' origin from both of these sources. Our analysis has afforded a rare look at early, critical events in primary plastid evolution and support the notion that integration of plastid-host metabolism was primarily driven by host-derived transporters with important contributions coming from the cyanobacterial endosymbiont and *Chlamydia*-like bacteria. Another class of proteins of currently unknown origin included plant specific transporters such as MEX1. Despite the power of our comparative approach, our work has some important limitations. One is that because we used the *Arabidopsis *transporter set, we most certainly have missed a number of Plantae transporters that are specific to red or green algae and have been lost from the *Arabidopsis *genome. In addition, we lack significant data from glaucophytes, but the upcoming *Cyanophora paradoxa *(glaucophyte) nuclear genome sequence [52] will allow us to incorporate this lineage into future inferences about transporter evolution. It is reasonable to assume, however, given the wealth of data supporting Plantae monophyly [2-4,7], that our inferences regarding the red and green lineages also apply to their glaucophyte sisters. Despite these limitations and the fact that phylogenetic signal is imperfectly maintained over a billion years of evolution, our comprehensive analysis of the chloroplast solute transport system will likely hold up and can be further tested as other genome sequences become available.

## Materials and methods

### Initial transporter analyses

As a starting point for the compilation of a conservative set of predicted or confirmed plastid envelope membrane transporters, we used a previously published list of 137 plastid-targeted membrane proteins that was based on predicted plastid localization and classification by the transporter classification system [10]. This list was manually curated to remove proteins from the list if published evidence indicated that they were localized to a cellular location other than chloroplasts, if they represented membrane-bound enzymes, or if they were annotated as components of the TIC/TOC protein import apparatus, the photosynthetic machinery of the thylakoid membrane, or the Sec or Tat protein targeting pathways. This curated list of candidate genes was updated and amended with recently published chloroplast envelope membrane transporters, such as AtFOLT1, a plastid localized transporter belonging to the mitochondrial carrier family that does not contain a plastid targeting signal [33] and was thus not included in previous lists. The final list contained 83 *A. thaliana *predicted or confirmed chloroplast solute transporters.

The sequence for each protein was obtained from The *Arabidopsis *Information Resource website [53]. These protein sequences were used as queries in blastp and tblastn searches of the NCBI Database [54], the plant and algal genomes available through the Joint Genome Institute [55], the *Cyanidioschyzon merolae *Genome Project website [56], the *Galdieria sulphuraria *Genome Project website [57], and Dragonblast V2.1 (SE Ruemmele, unpublished data), a web based database in the DB lab that contains EST datasets for several chromalveolates, Plantae, excavates, Rhizaria, and Amoebozoa. We used the predicted protein sequences for the following species for our analysis whenever available: *Arabidopsis thaliana*, *Oryza sativa*, *Physcomitrella patens*, *Chlamydomonas reinhardtii*, *Ostreococcus tauri*, *Ostreococcus lucimarinus*, *Cyanidioschyzon merolae*, *Galdieria sulphuraria*, *Cyanophora paradoxa*, *Dictyostelium discoideum*, *Strongylocentrotus purpuratus*, *Xenopus laevis*, *Danio rerio*, *Mus musculus*, *Canis familiaris*, and *Homo sapiens*. In addition, we included at least one insect, three fungal species, and a broad range of Bacteria and Archaea in our analysis. The BLAST searches used an e-value cut-off < 10^-5^. If a translated EST sequence was not available, the nucleotide sequence was translated over six frames using the ExPASy translate tool [58]. The resulting protein sequences were used in a BLAST search against the NCBI protein database to ensure the correct translation was obtained.

We used the ClustalW feature included with BioEdit V7.0.5.3 to generate protein alignments [59]. Alignments were visually inspected and manually corrected if necessary. Trees were generated under maximum likelihood using PHYML V2.4.4 utilizing the WAG model of amino acid substitution and estimating both the proportion of invariable sites and the alpha parameter (that is, WAG + I + Γ)[60]. We performed non-parametric bootstrap analysis with 100 replicates for each PHYML analysis. The resulting trees were analyzed to determine the origin of the transporter in *Arabidopsis *and other Plantae.

The designation of gene origin was done as follows. When the Plantae solute transporter formed a well-supported (usually > 70% bootstrap support) monophyletic group with homologs in opisthokonts (that is, animals and fungi) and secondarily with other eukaryotes such as excavates and chromalveolates (if present), then it was classified as having a 'Host' origin. Under this scheme, no bacterial sequences interrupted the eukaryotic domain. 'Cyanobacterial' or '*Chlamydia*-like' origin was inferred if the Plantae sequence formed a monophyletic group with protein sequences from either of these lineages with strong bootstrap support. Other bacterial or eukaryotic sequences could (not necessarily) be in these trees but there had to be a robust separation of the Plantae + cyanobacteria or *Chlamydia*-like clade from all other homologs. We had two other categories of gene origin that likely reflected a lack of phylogenetic resolution or pervasive HGT among taxa that defied a clear inference of origin. The first was the 'Other' category in which the Plantae transporter formed a well-supported monophyletic clade but its position relative to available bacterial data was unresolved, thereby not allowing us to identify the donor taxon. The second, 'Plantae-specific', was for transporters that had no significant hits to sequences in GenBank or other databases and appeared to be limited to the Plantae. All of the transporter PHYML bootstrap trees are available in Additional data file 1. The protein alignments are available in the download section of the Bhattacharya Lab website [61].

### Detailed phylogenetic analyses

For eight representative transporters from the five categories described above we inferred a maximum likelihood phylogeny using RAxML (RAxML-VI-HPC, v2.2.1) [62] and the WAG + Γ evolutionary model. The specific transporters were: 'Host' - At4g39460 (SAMT, S-adenosylmethionine carrier 1 protein, 236 amino acids), At5g66380 (AtFOLT1, *Arabidopsis thaliana *folate transporter 1, 268 amino acids); 'Cyanobacterial' - At1g19800 (TGD1, trigalactosyldiacylglycerol 1, lipid transporter, 234 amino acids), At5g64940 (ABC transporter protein, 487 amino acids); '*Chlamydia*-like' - At1g15500 (chloroplast ADP, ATP carrier protein 2, 436 amino acids), At4g37270 (HMA1, copper exporting ATPase, 444 amino acids); 'Other' - At1g32080 (putative membrane protein, 211 amino acids); 'Plantae-specific' - At5g24690 (hypothetical expressed protein, 274 amino acids). These detailed analyses used a random starting tree (one round of taxon addition) and the rapid hill-climbing algorithm (that is, option -f d in RAxML). To generate bootstrap values for these phylogenies, we used RAxML with the same settings and 100 replications. In addition, we used Bayesian inference (MrBayes V3.0b4) [63] with each of the eight data sets using the WAG + I + Γ model to calculate posterior probabilities for nodes in the RAxML trees. Metropolis-coupled Markov chain Monte Carlo from a random starting tree was used in this analysis with two independent runs (that is, nrun = 2 command) and 1 cold and 3 heated chains. The Bayesian analyses were run for two million generations each with trees sampled every 100th generation. To increase the probability of chain convergence, we sampled trees after the standard deviation values of the two runs were < 0.01 to calculate the posterior probabilities. We also ran the Bayesian analysis for the remaining two putative '*Chlamydia*-like' genes in Plantae (dicarboxylate translocators DiT1, DiT2.1, and DiT2.2, and the phosphate transporter PHT2;1) to assess the topologies. We incorporated a representative diversity of available sequences in all of these trees.

## Abbreviations

AtFOLT, *A. thaliana *folate transporter; AtNTT, *A. thaliana *ADP/ATP translocase; DiT, dicarboxylate translocator; EST, expressed sequence tag; HGT, horizontal gene transfer; HMA, heavy metal ATPase; TGD, trigalactosyldiacylglycerol.

## Authors' contributions

APMW and ML gathered and prepared the *Arabidopsis *plastid transporter data for downstream bioinformatic analyses. HMT did the subsequent database searches and built the initial phylogenetic trees. DB was responsible for the final phylogenetic trees presented in the manuscript figures. HMT wrote the initial draft of the manuscript. APMW and DB conceived of and supervised this study and prepared the final manuscript. All authors read and approved the final manuscript.

## Additional data files

The following additional data are available with the online version of this paper. Additional data file 1 shows trees of all remaining plastid transporters analyzed in this study

## Supplementary Material

Additional data file 1 PHYML bootstrap trees of plastid targeted solute transporters of putative 'Host', 'Cyanobacterial', '*Chlamydia*-like', 'Other', and 'Plantae-specific' origin found in our study. Click here for file
