# Supplementary material for: Host origin of plastid solute transporters in the first photosynthetic eukaryotes
Source: Genome Biol. 2007 Oct 5;8(10):R212. doi: 10.1186/gb-2007-8-10-r212 (PMC2246286; doi:10.1186/gb-2007-8-10-r212)

Supplementary Material:

Host Origin of Plastid Solute Transporters in the First Photosynthetic Eukaryotes

Heather M Tyra, Mark Linka, Andreas P.M. Weber, and Debashish Bhattacharya

Figure S1. Plastid targeted solute transporters of putative “Host” origin in Plantae. Thirty-one unique genes fall in this category, of which 30 are shown here. A detailed analysis of the 31st gene (the nucleotide-sugar/triose phosphate translocator gene family has been previously published, Weber et al. 2006). These are PHYML trees with the numbers above the branches inferred from a PHYML bootstrap analysis. Only bootstrap values ≥ 60% are shown. Branch lengths are proportional to the number of substitutions per site (see scale bars). The different photosynthetic groups are shown in different text colors: blue for cyanobacteria, red for red algae, green for green algae and land plants, magenta for glaucophytes, and brown for chromalveolates. In addition, the fungi are shown in pink text. The inclusion of chromalveolates within the Plantae is believed to reflect horizontal or endosymbiotic gene transfer events (e.g., Li et al. 2006).

At2g13100: MSF2 Carbohydrate Transporter

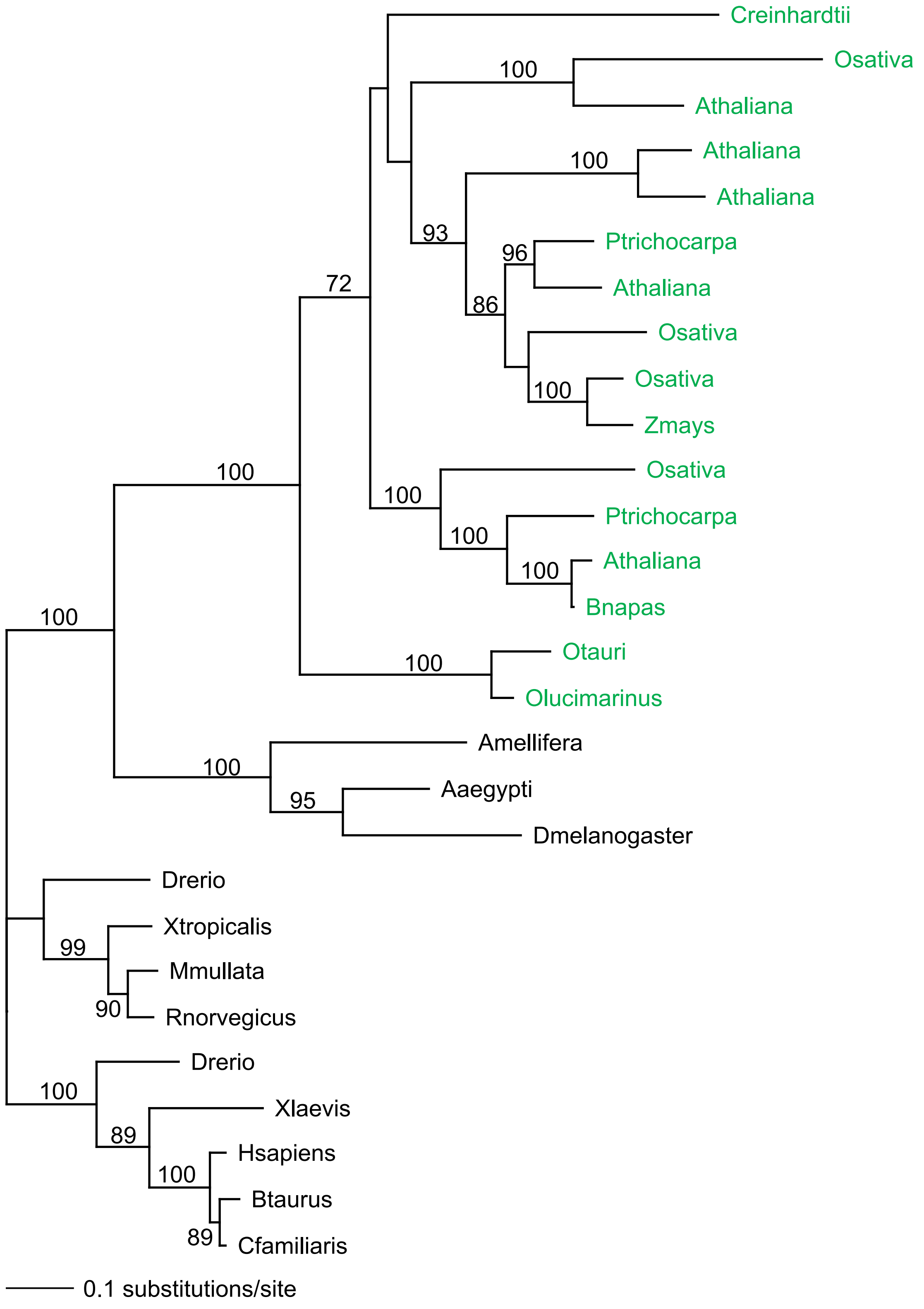

At3g17690, At3g17700: Cyclic Nucleotide-Binding Transporter

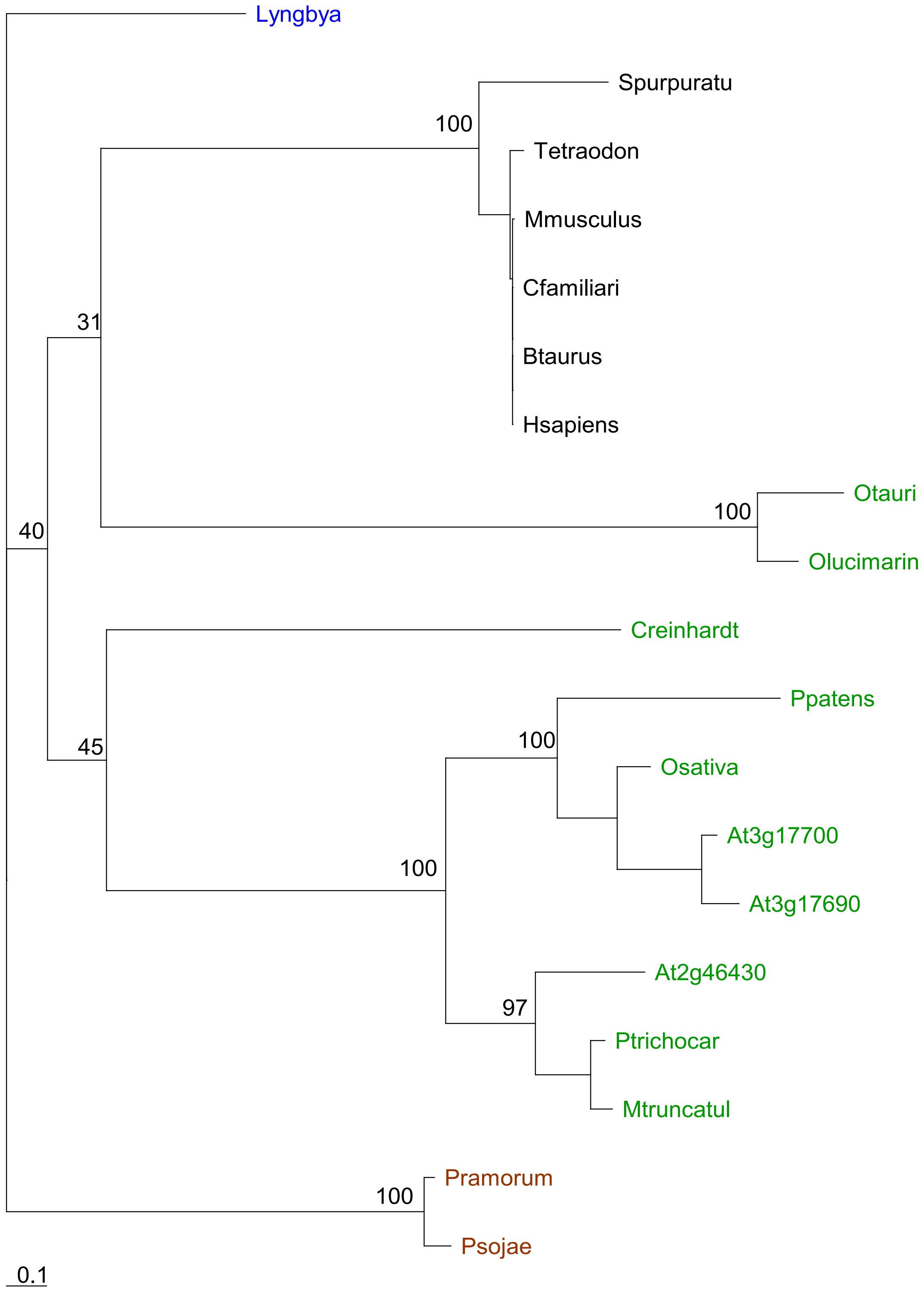

At4g32400: Adenine Nucleotide Antiporter

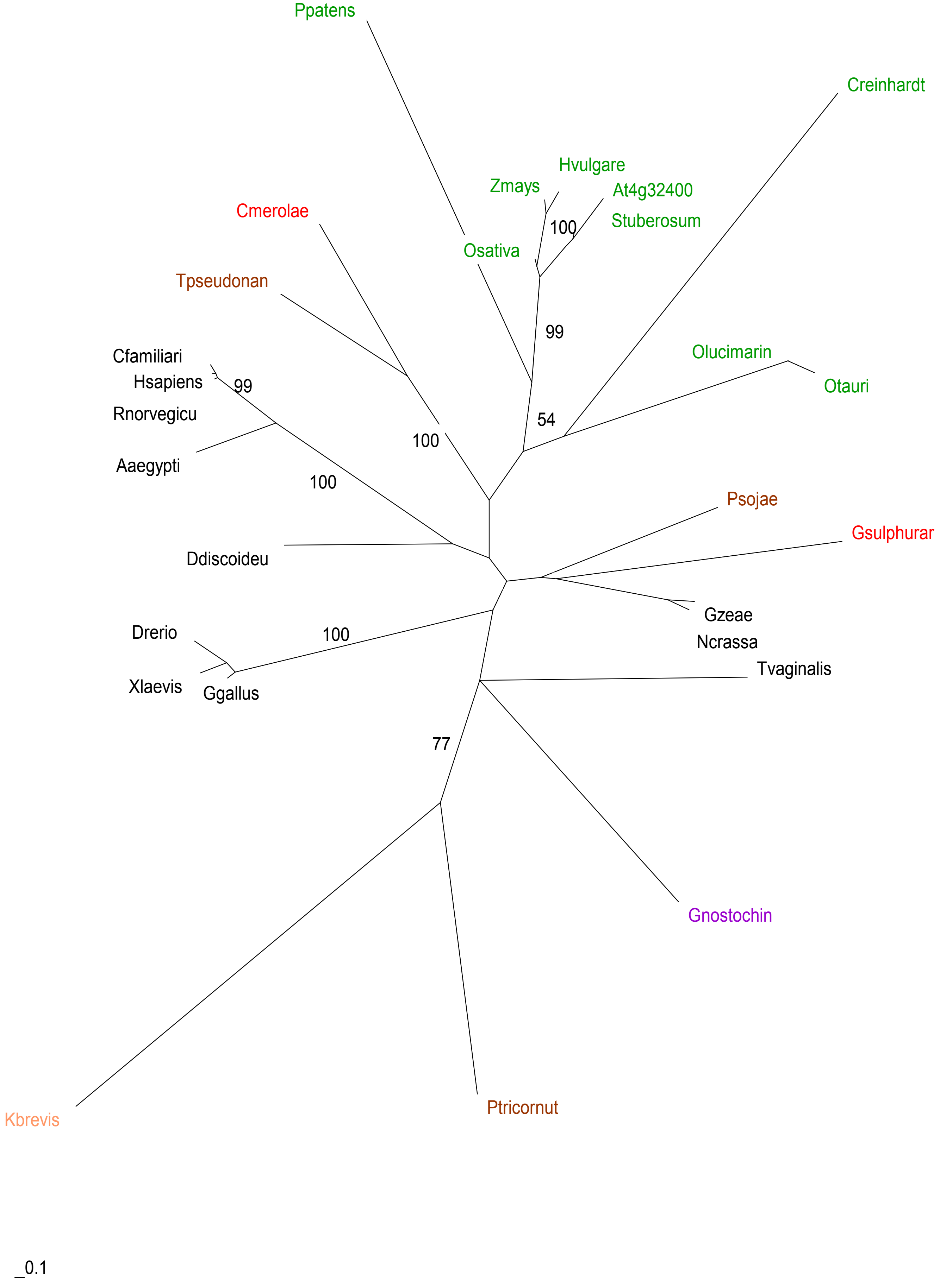

AT5G04770: Amino Acid Permease

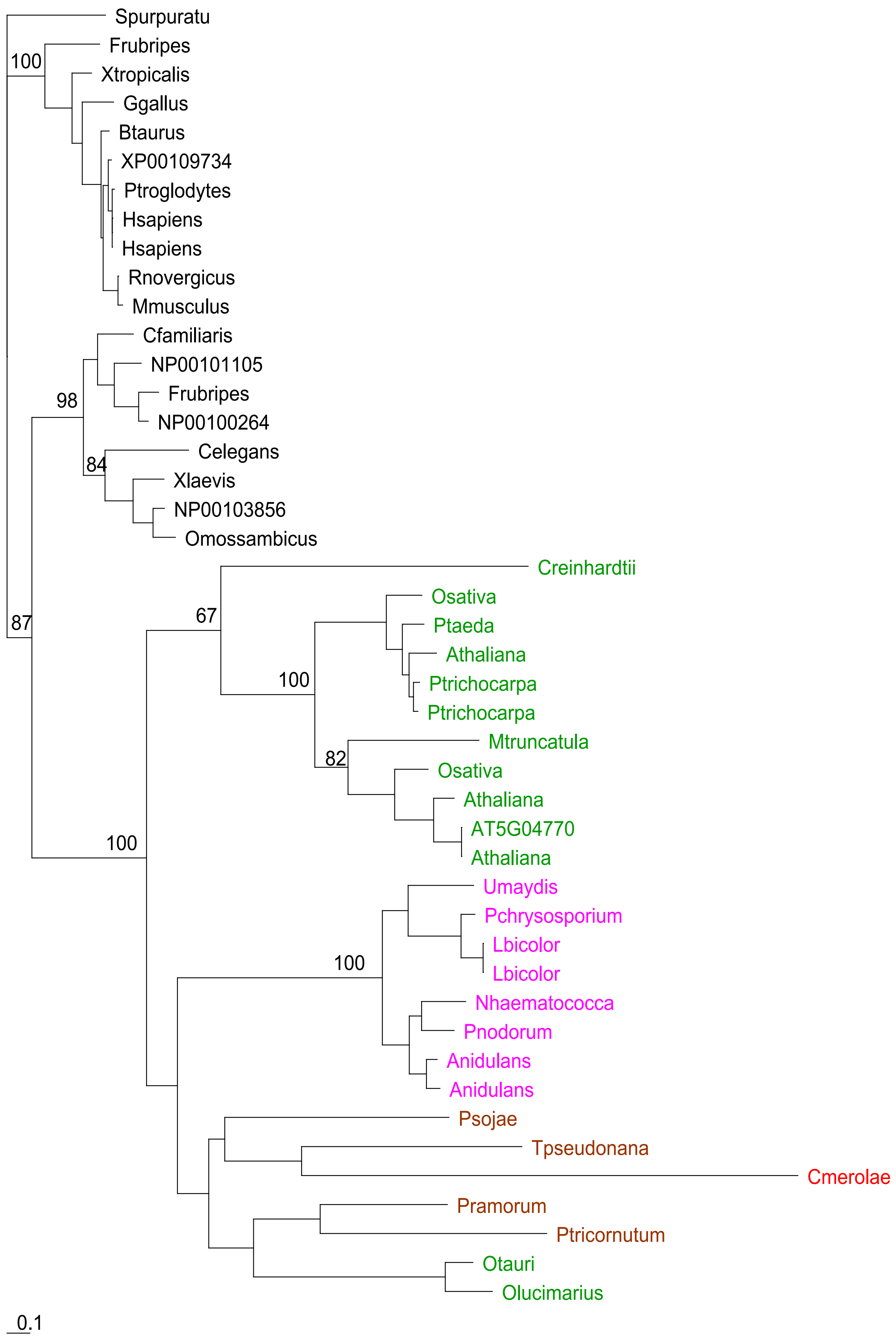

AT2G28070, AT3G52310: ABC Transporter

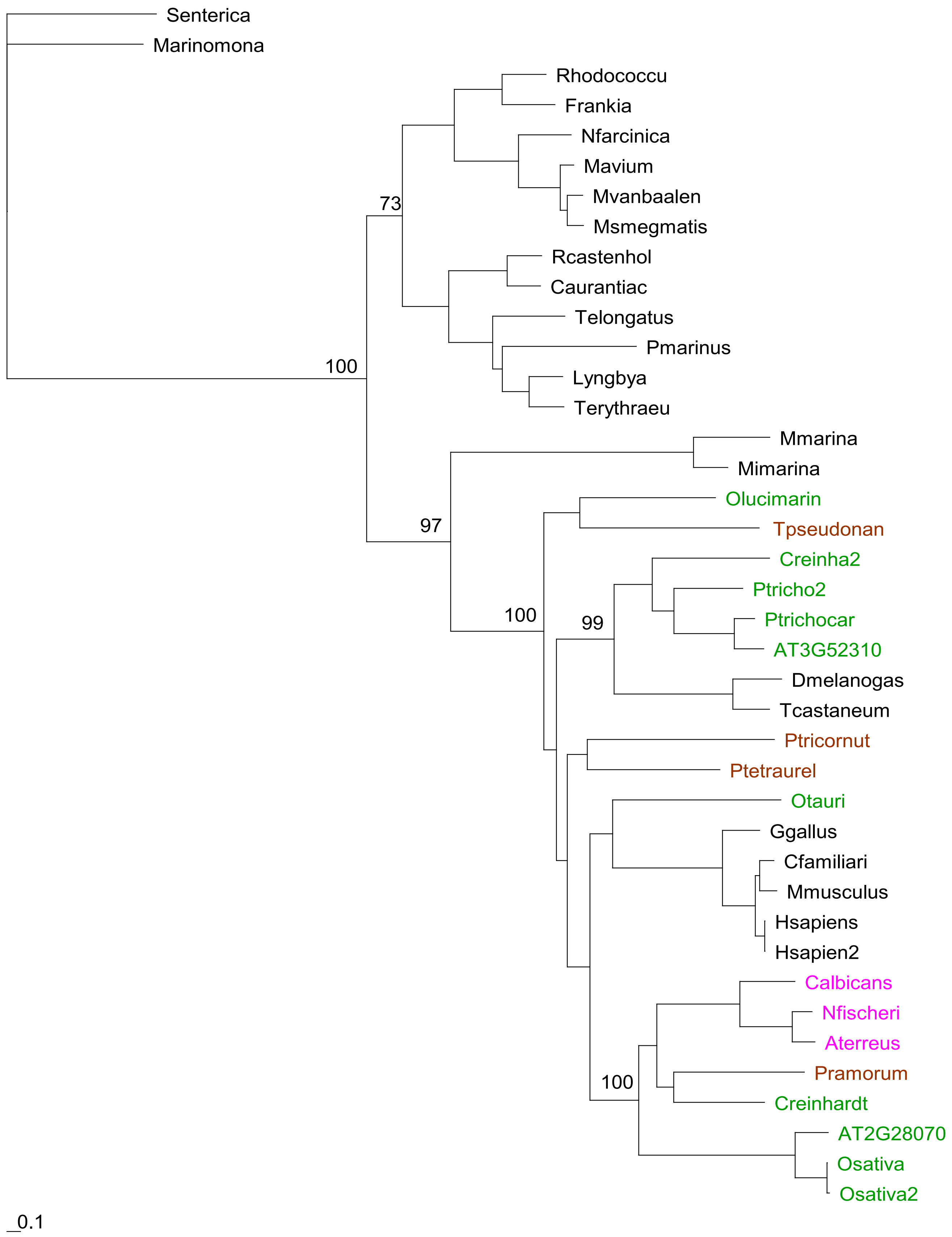

AT1G59870, AT1G66950: ABC Transporter (PDR)

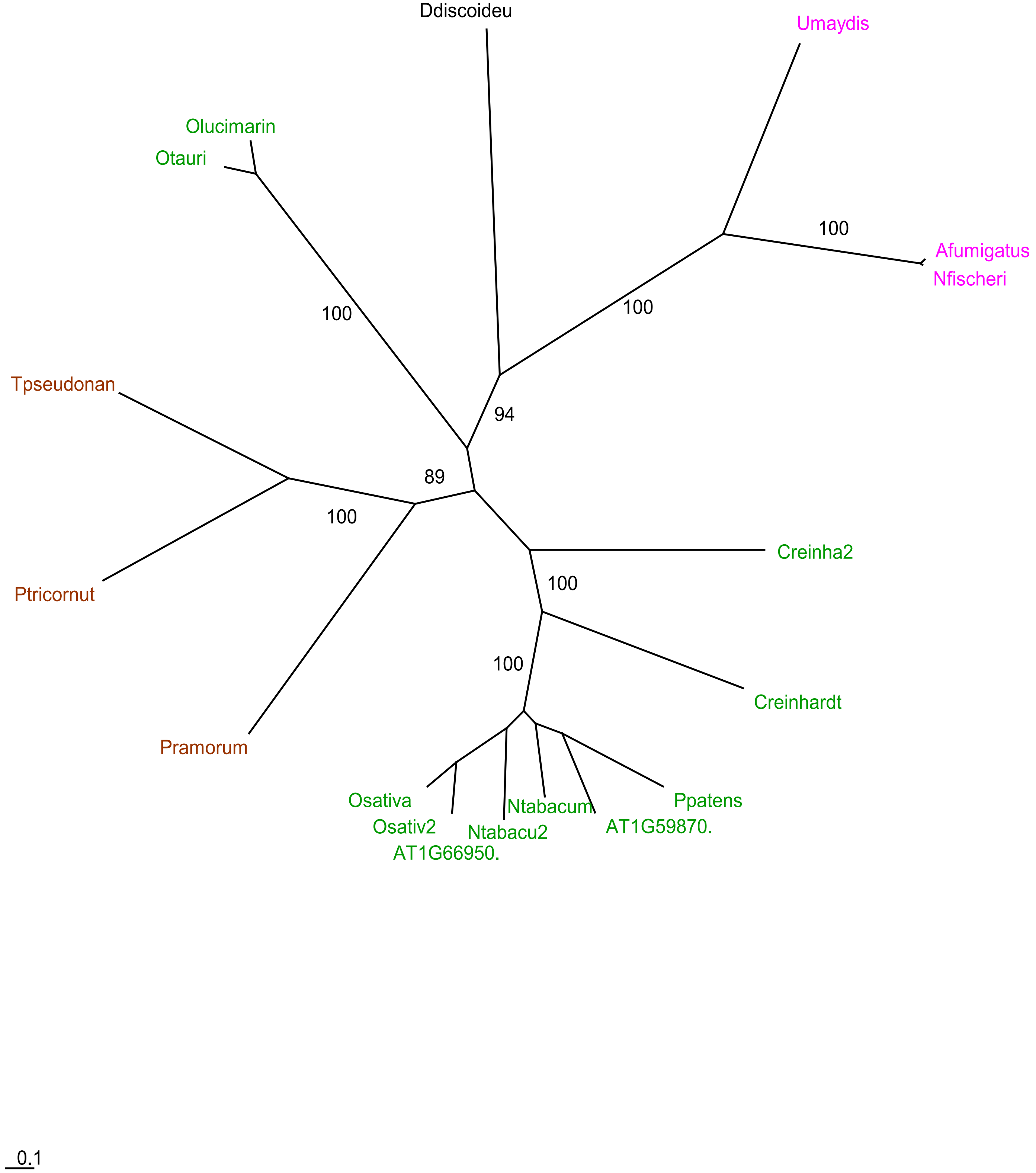

AT5G19410, AT5G52860, AT4G25750: ABC Transporter (White)

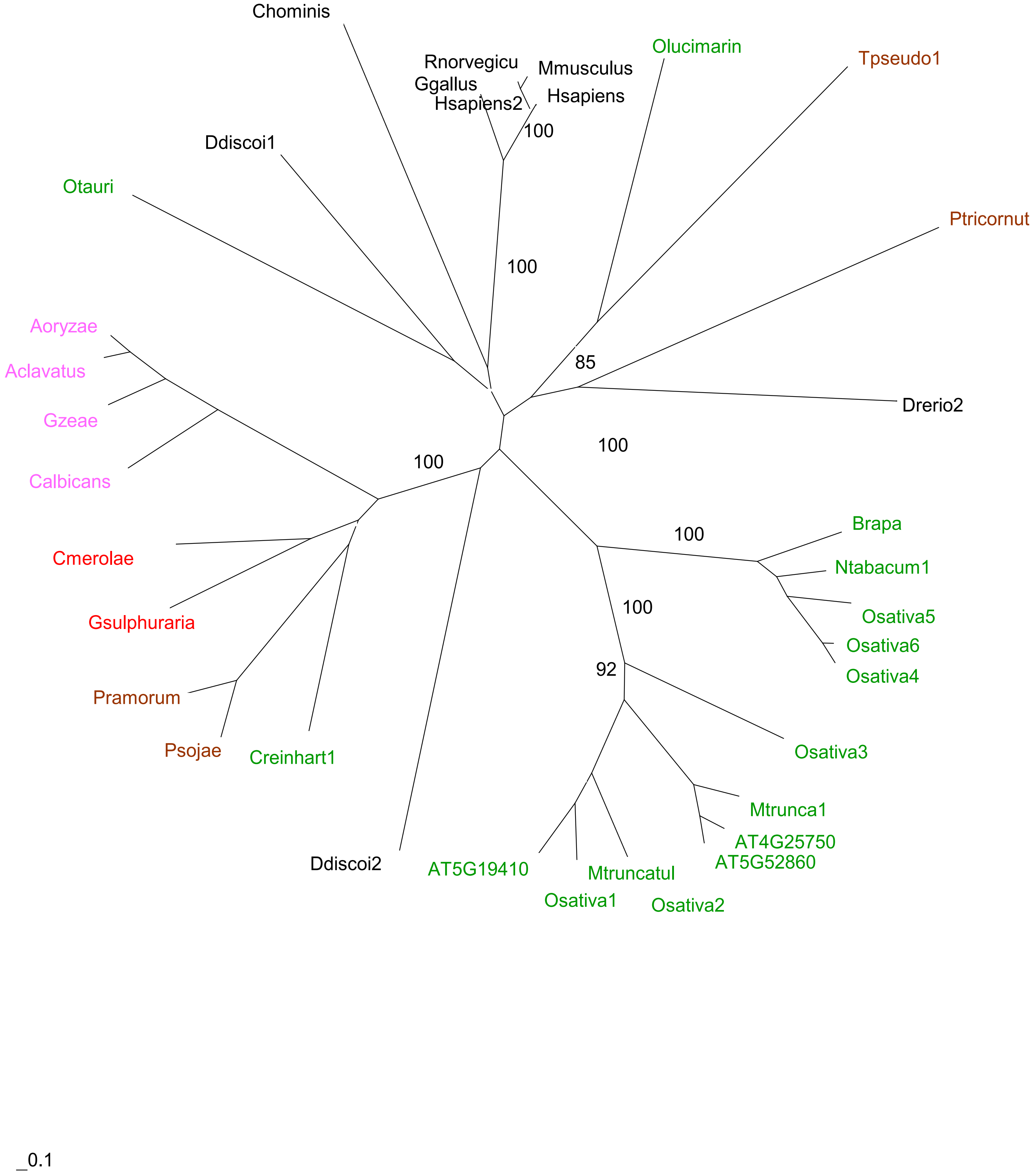

AT1G64150, AT1G13590: Expressed Protein

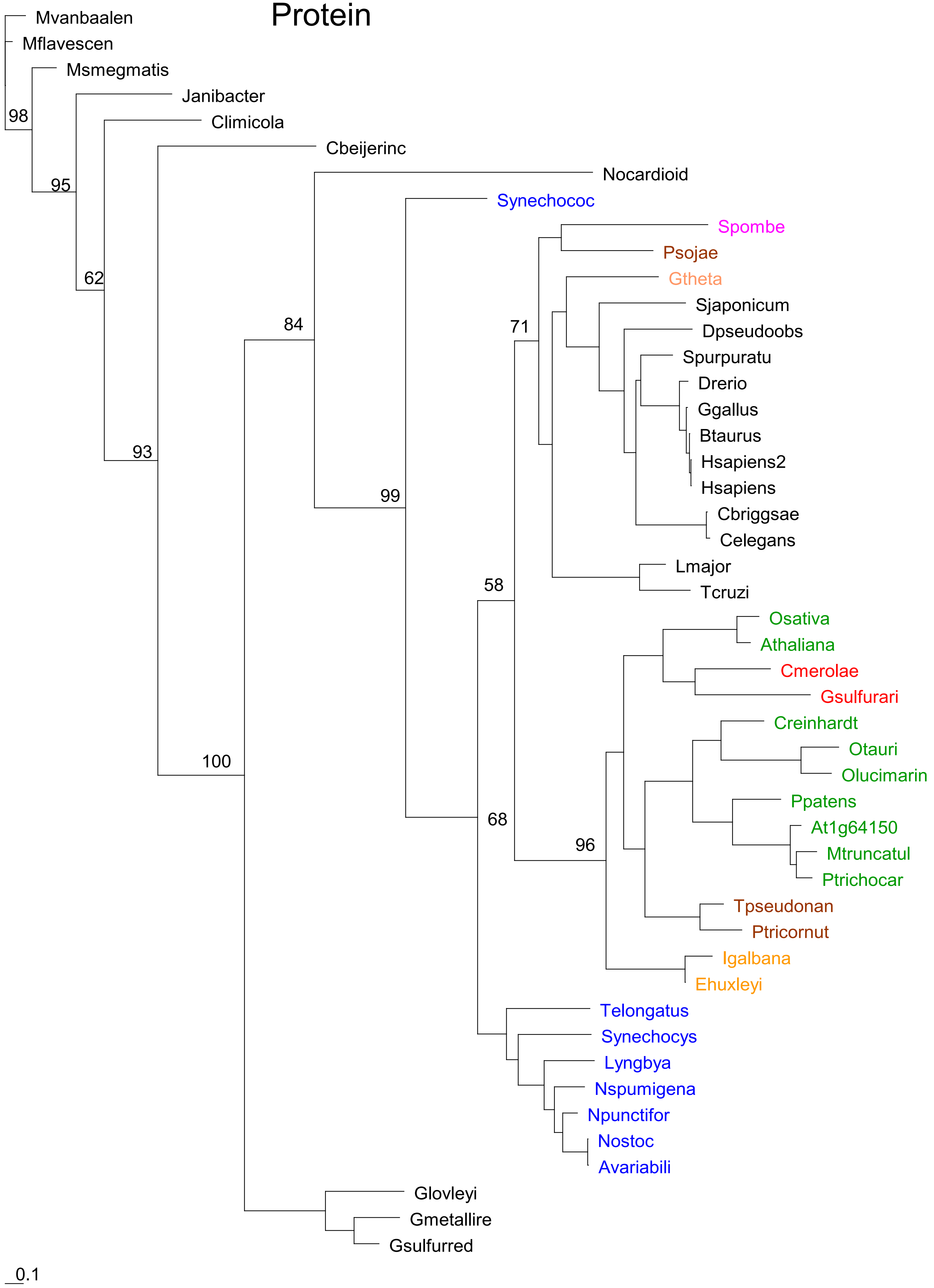

AT1G70610: ABC Transporter (TAP1)

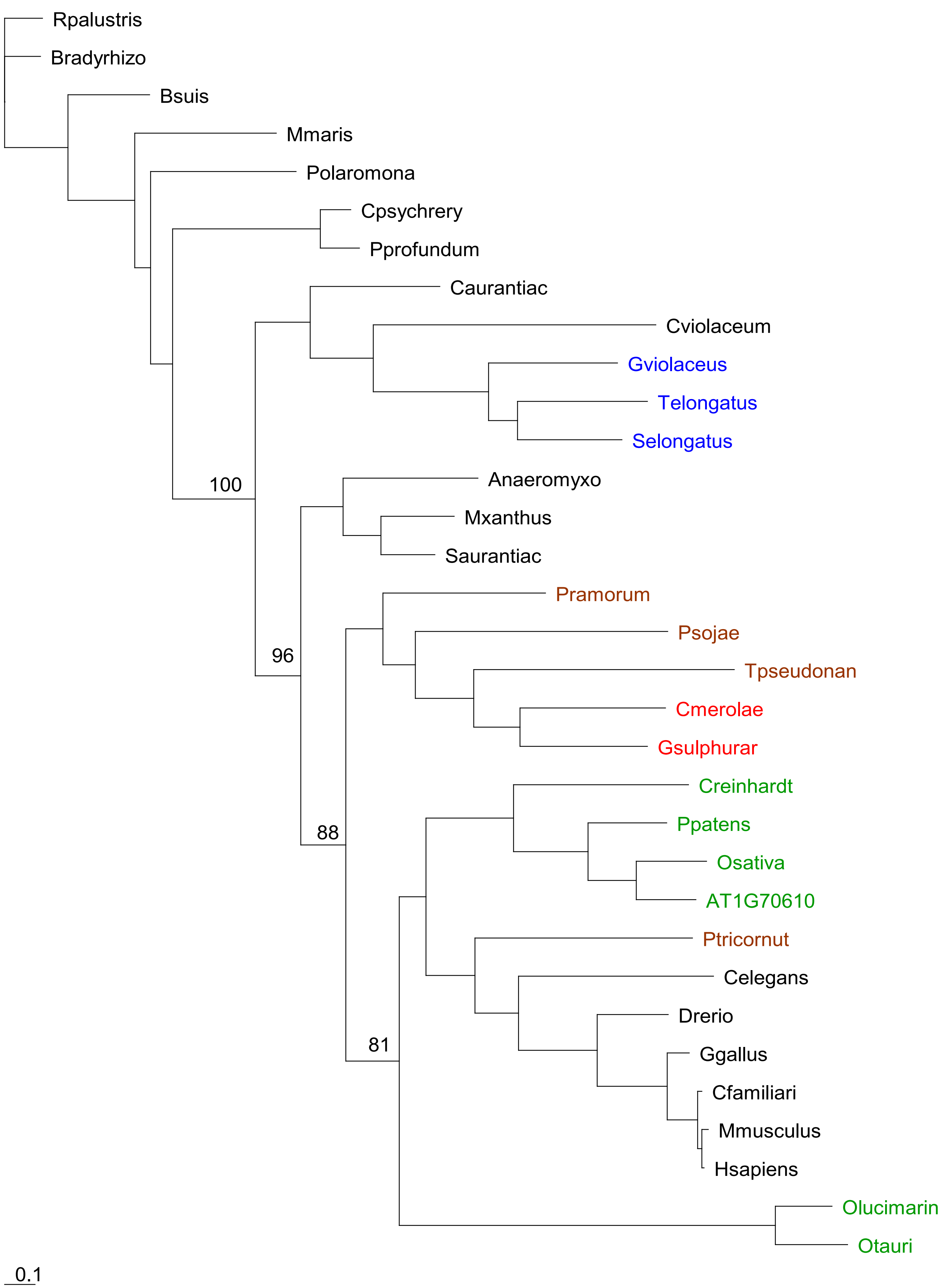

AT2G04620: Cation Efflux Family Protein

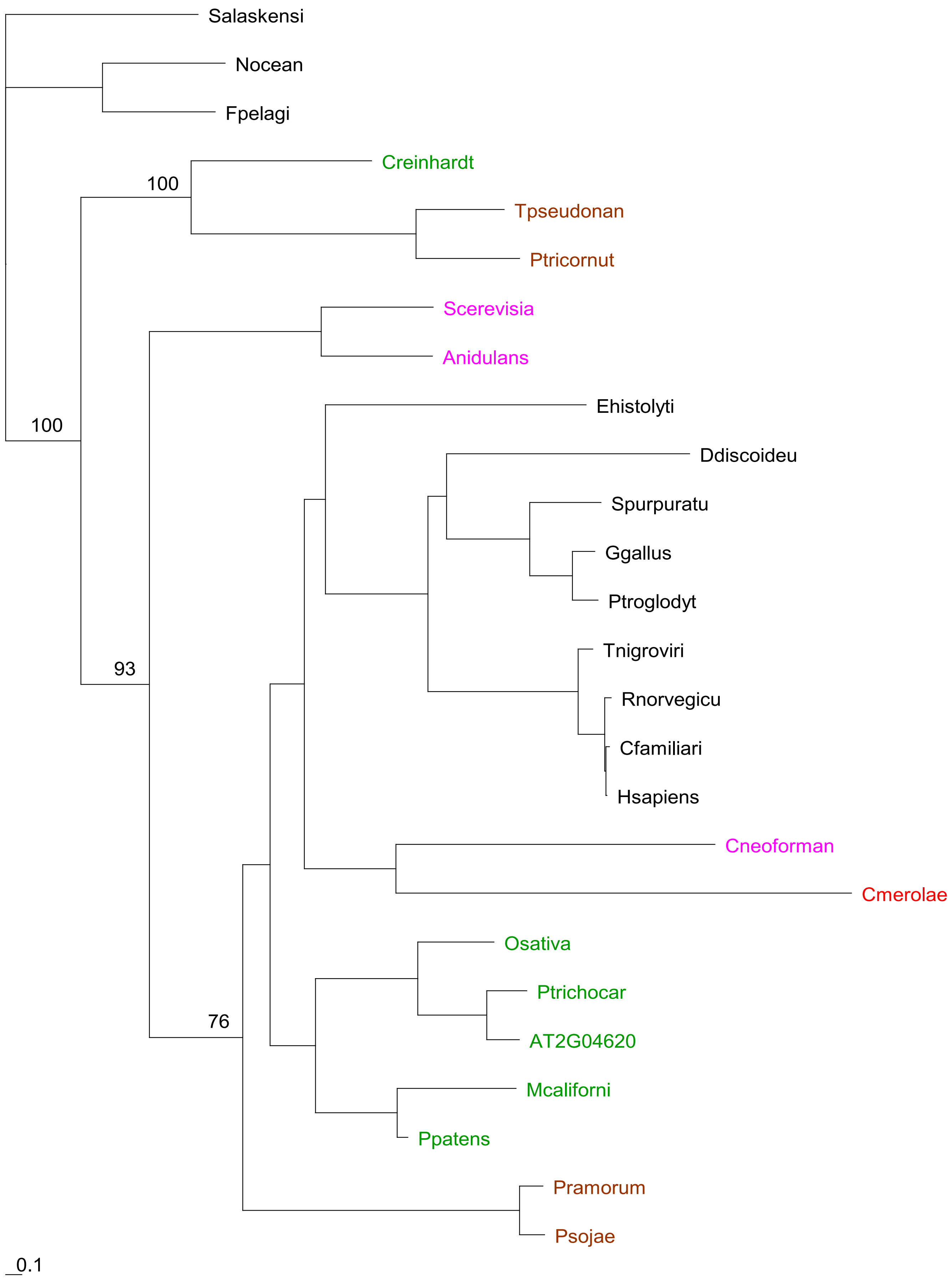

AT2G27810: Xanthine/Uracil Permease

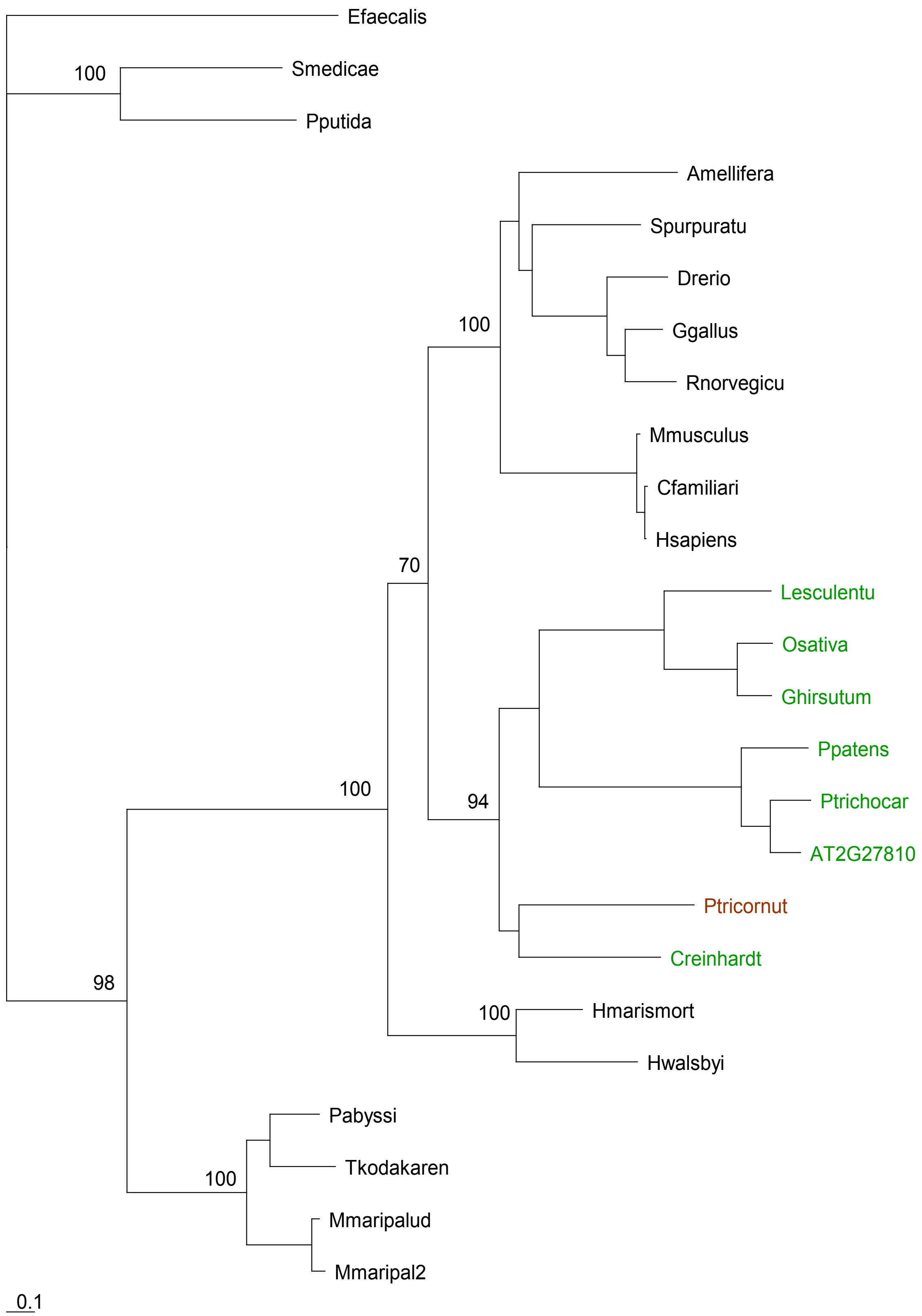

AT2G29650: Na+-dependent Inorganic Phosphate Cotransporter

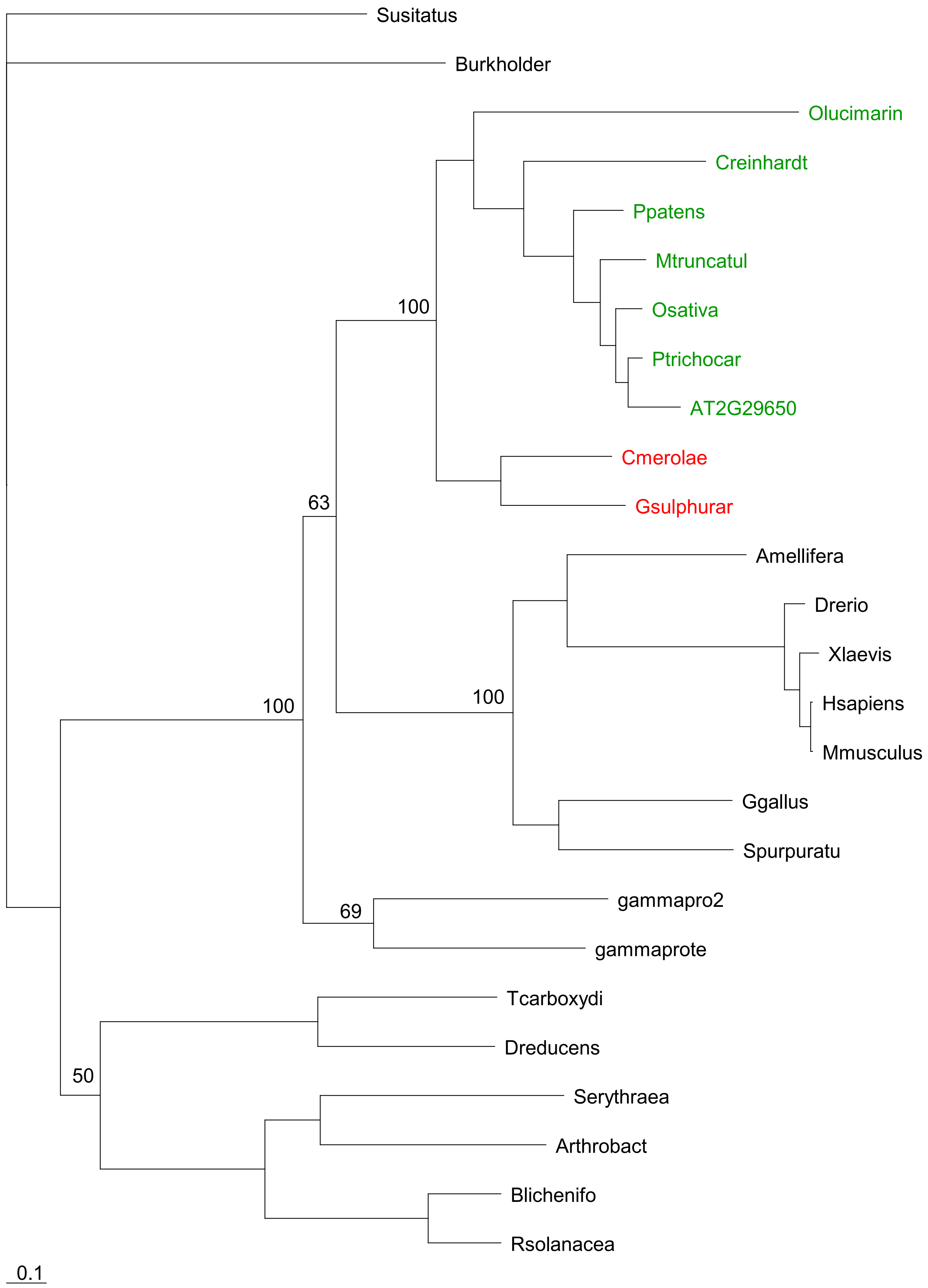

AT2G40420: Amino Acid Transporter

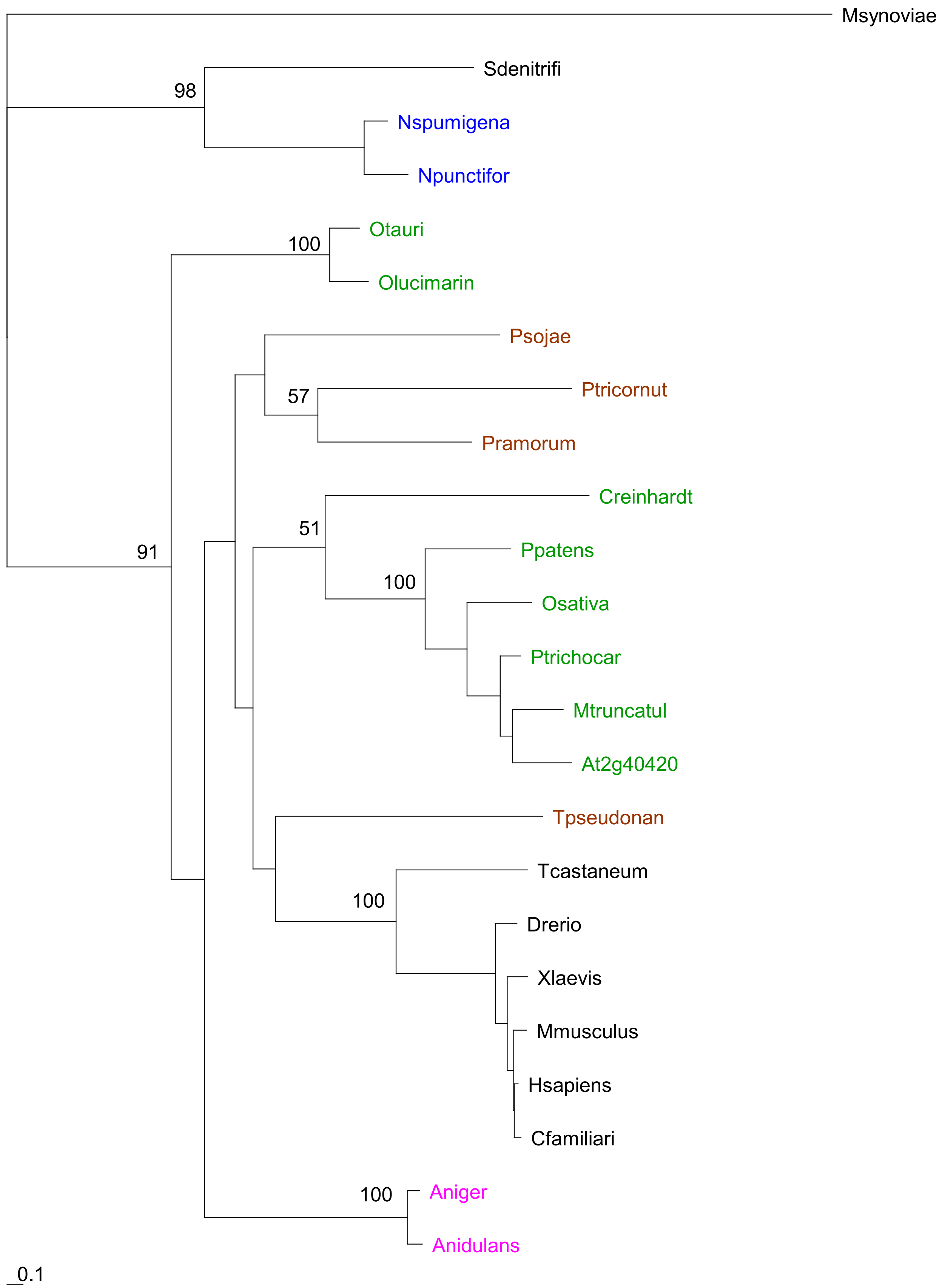

AT3G45890: Expressed Protein

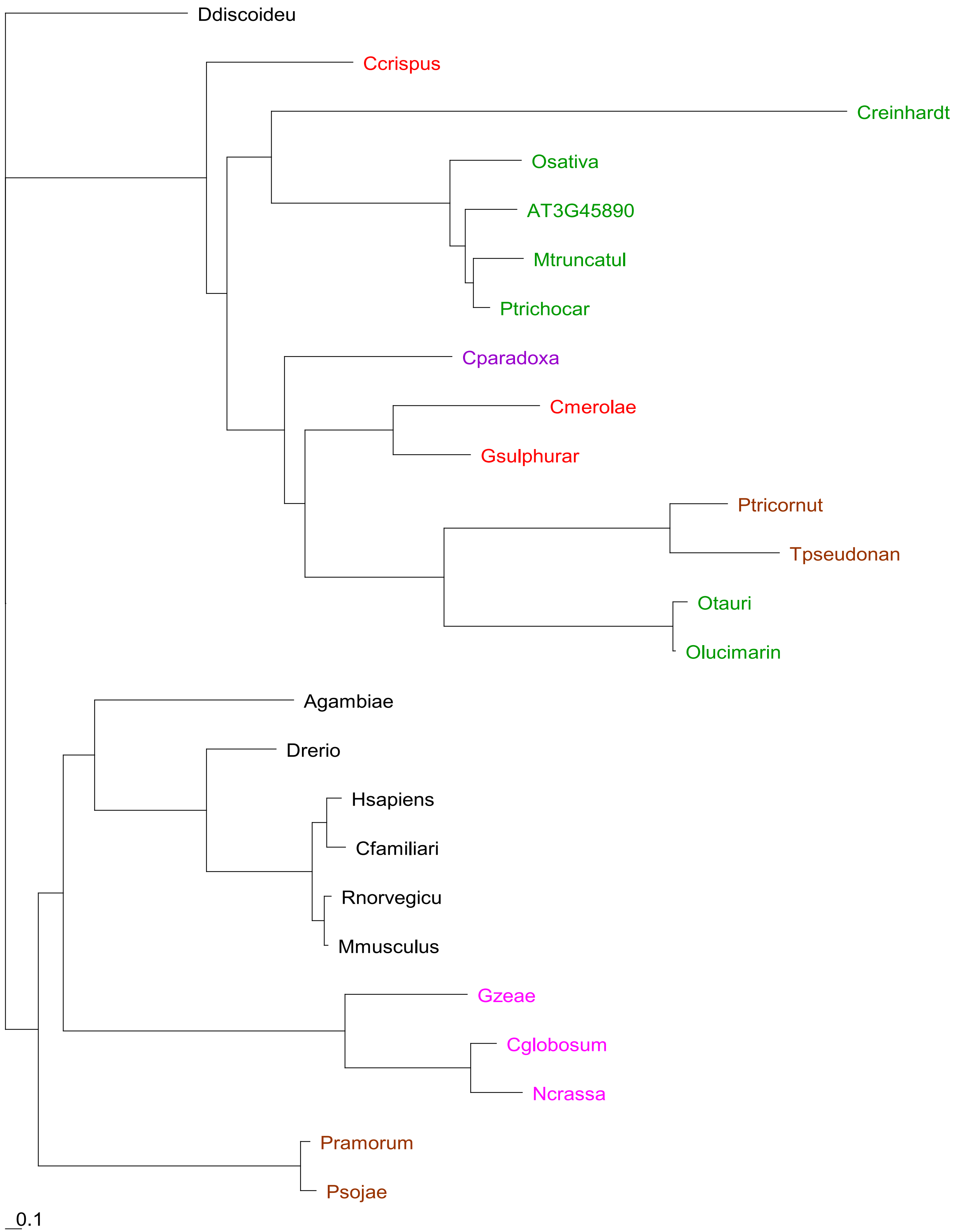

AT4G00370: AtNTR2

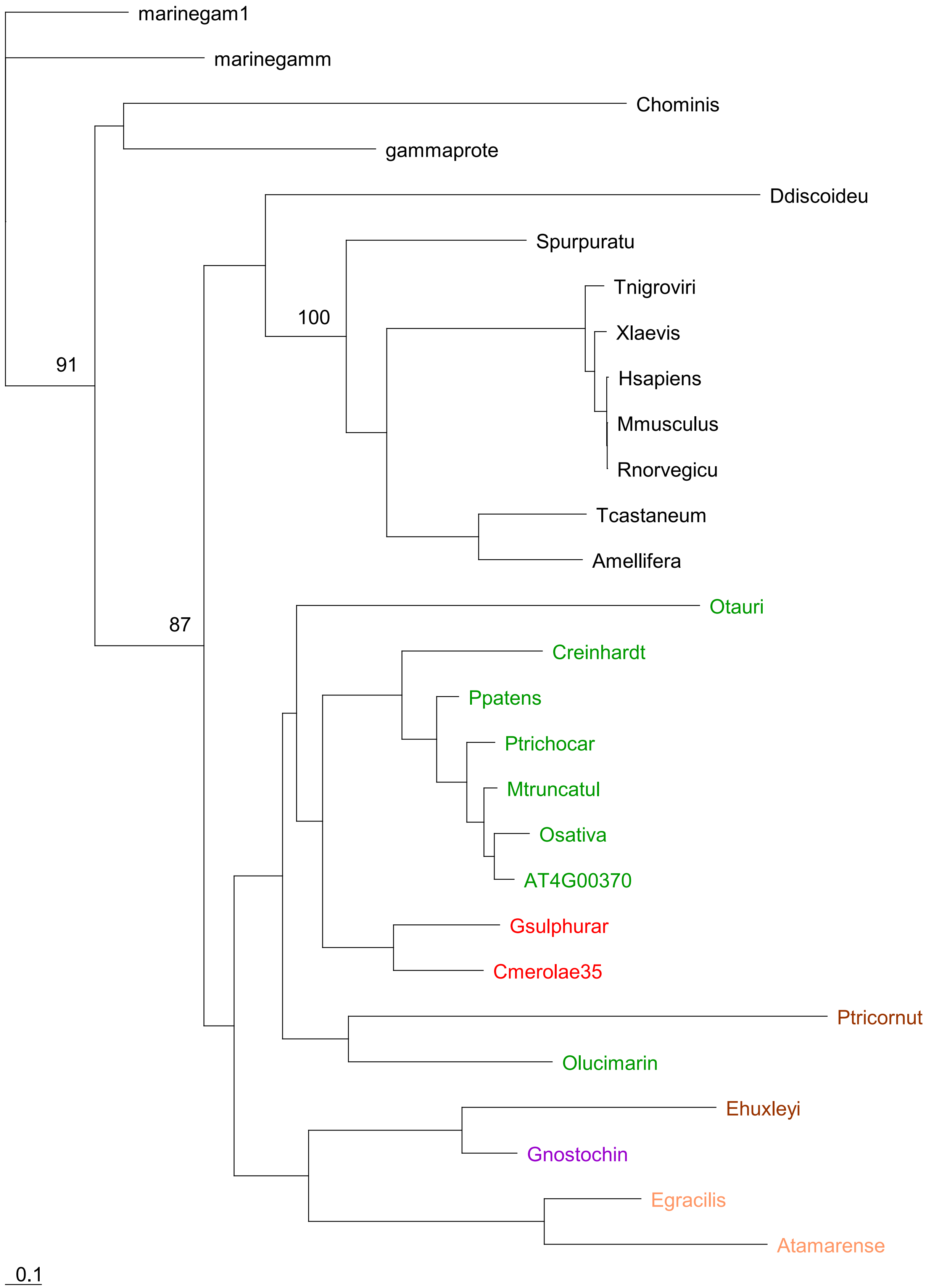

AT4G17340: Major Intrinsic Family Protein

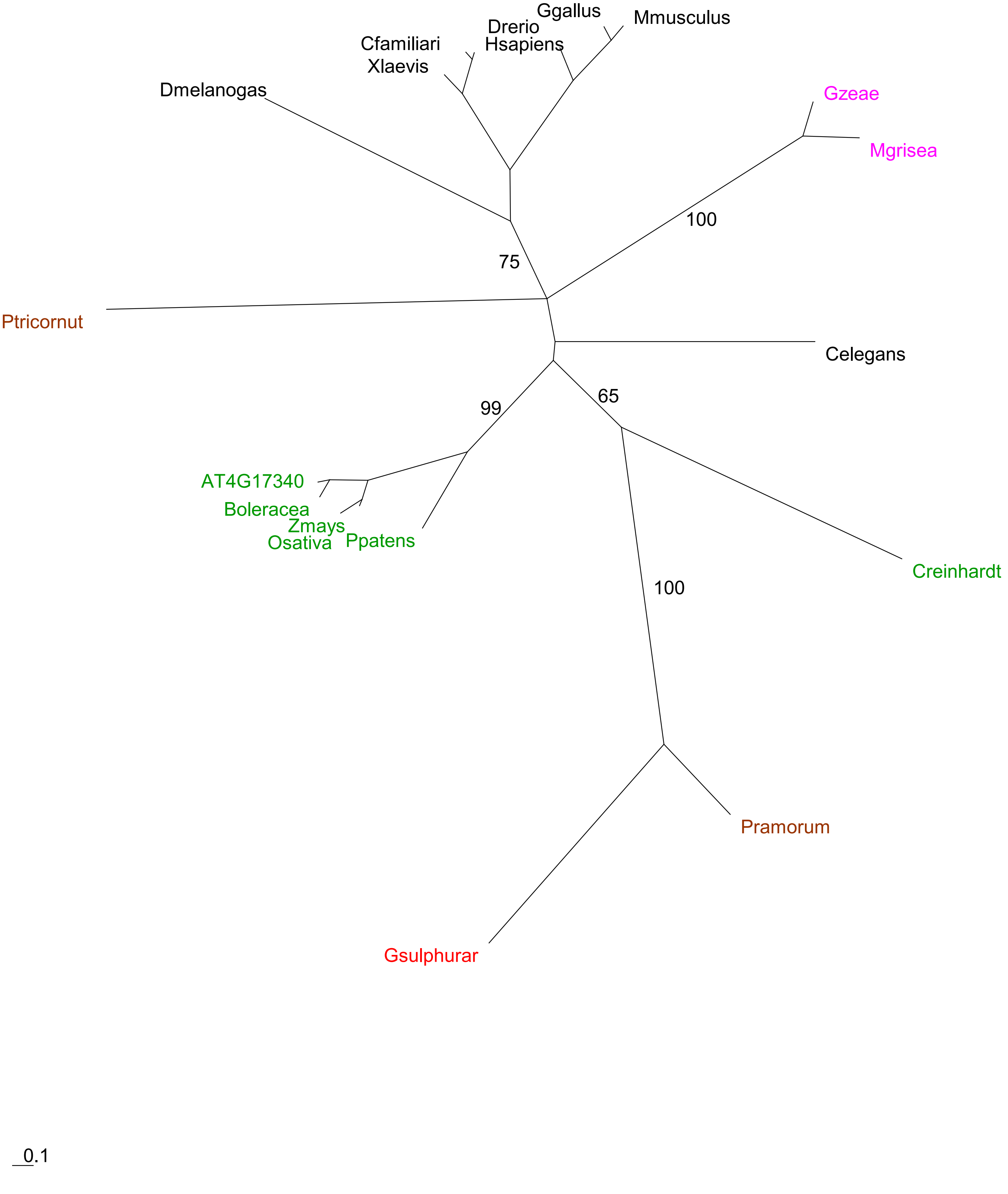

AT4G32650: KAT3

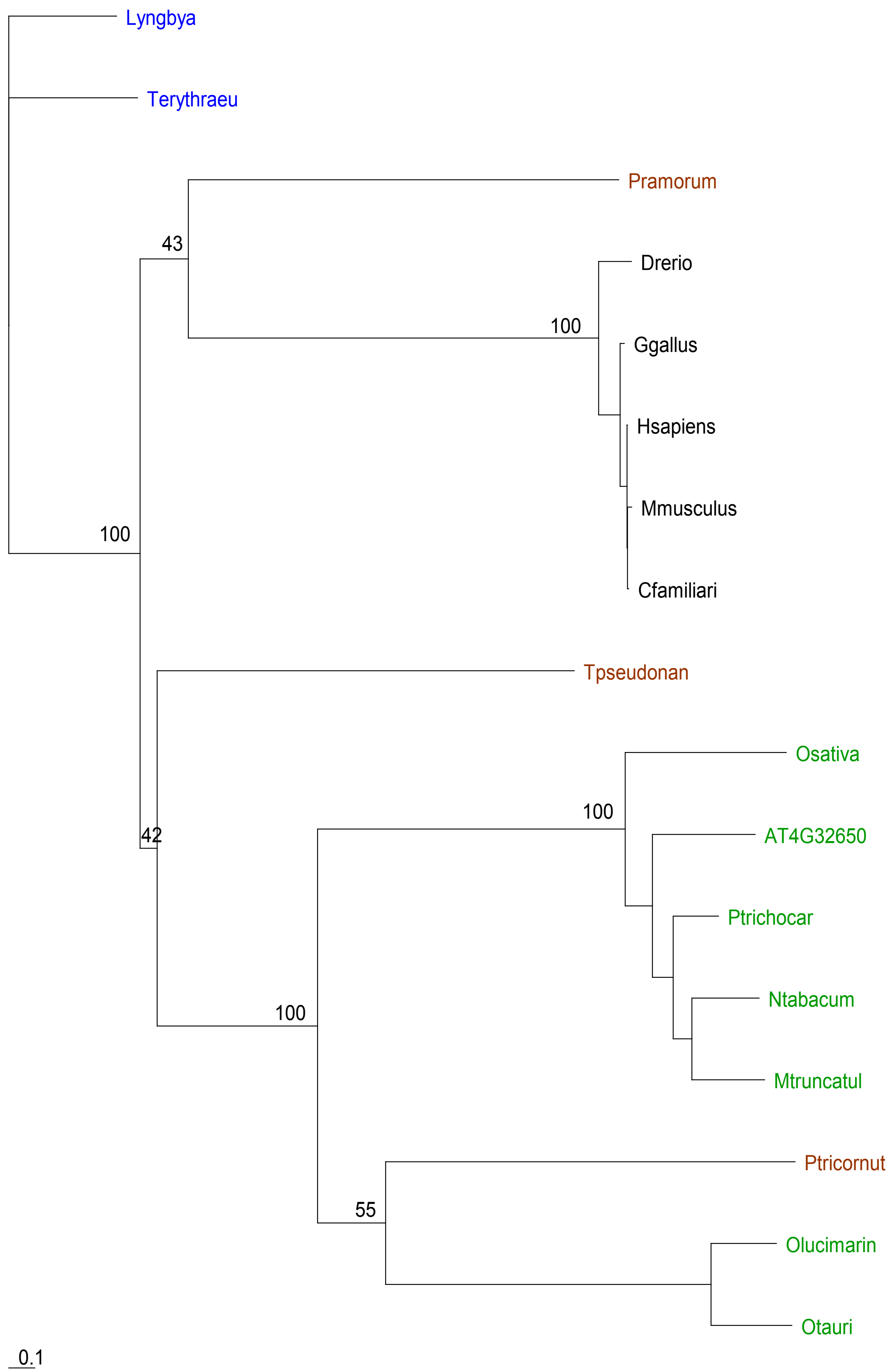

At4g39460: SAMT

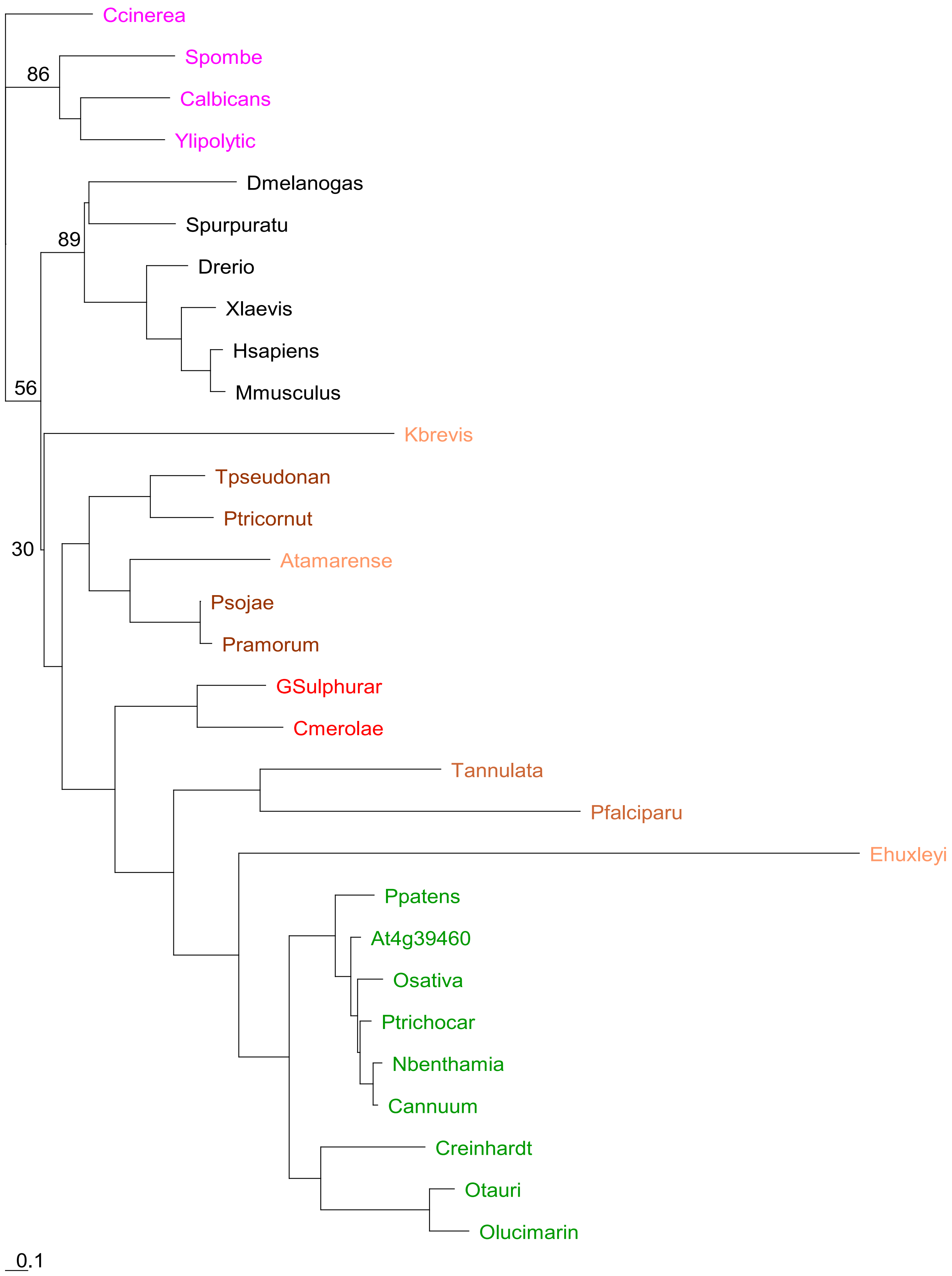

AT5G13550: Sulfate Transporter

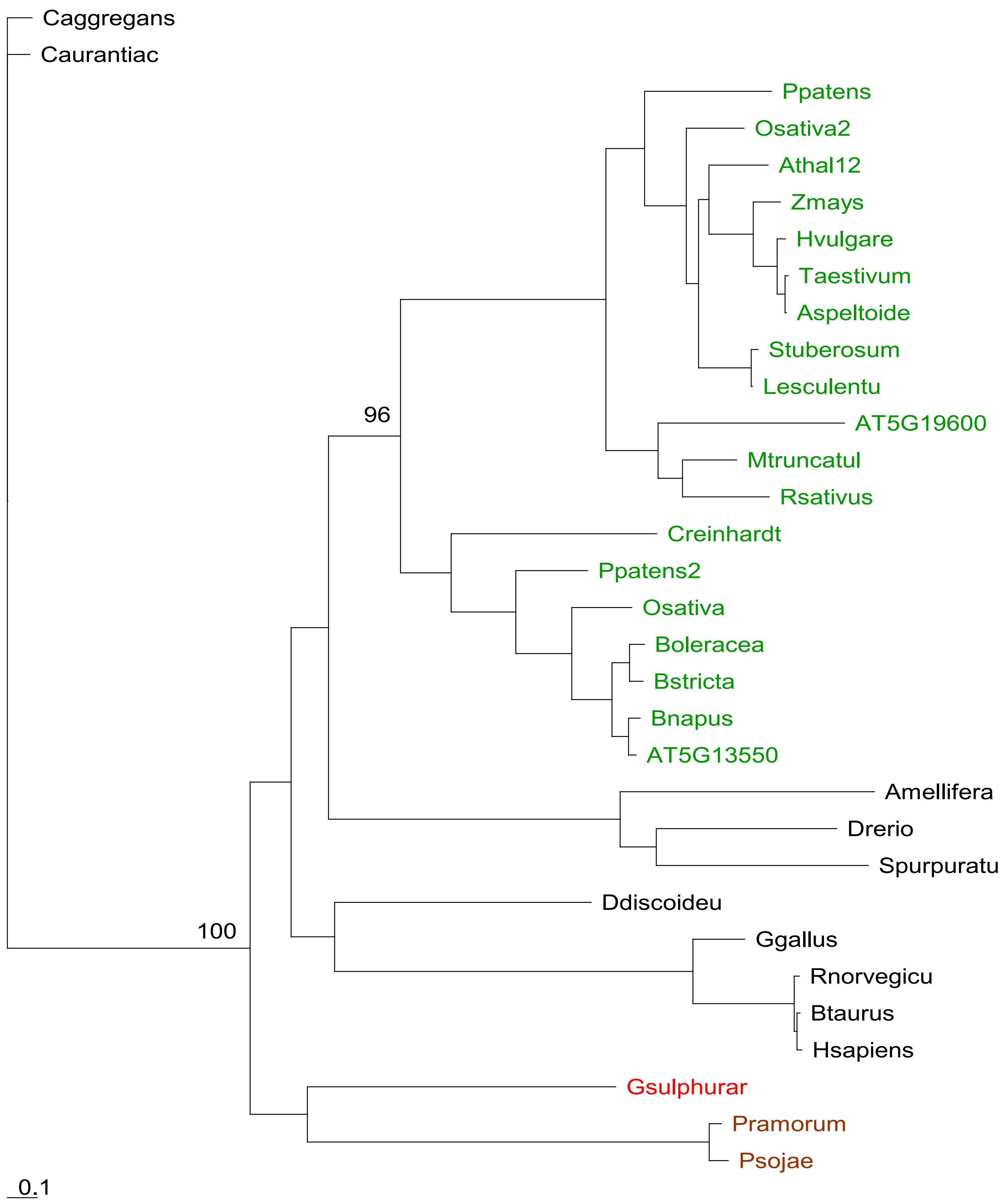

AT5G14040: Mitochondrial Phosphate Transporter

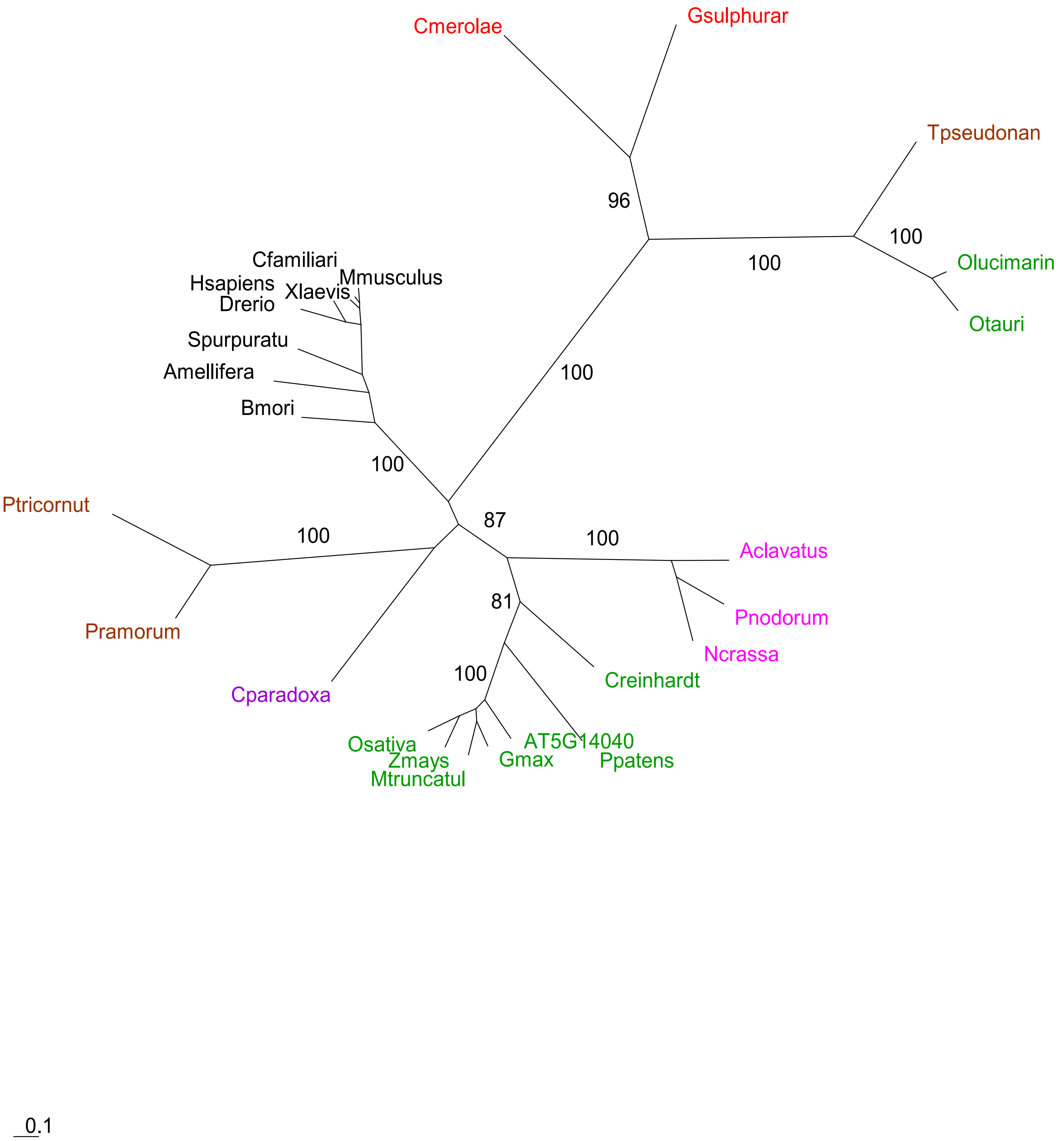

AT5G22830: CorA-like Magnesium Transporter

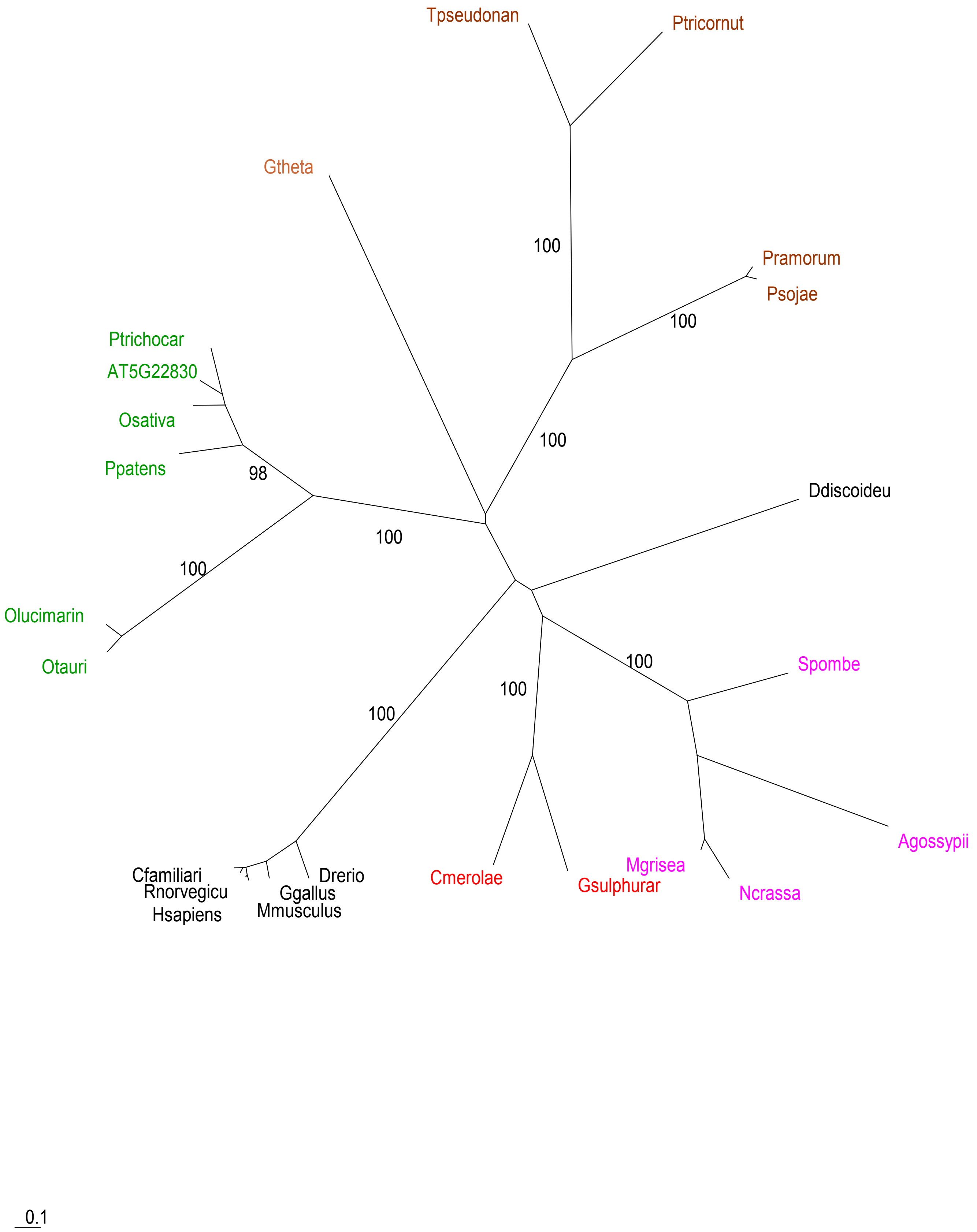

AT5G26820: Ferroportin-Related

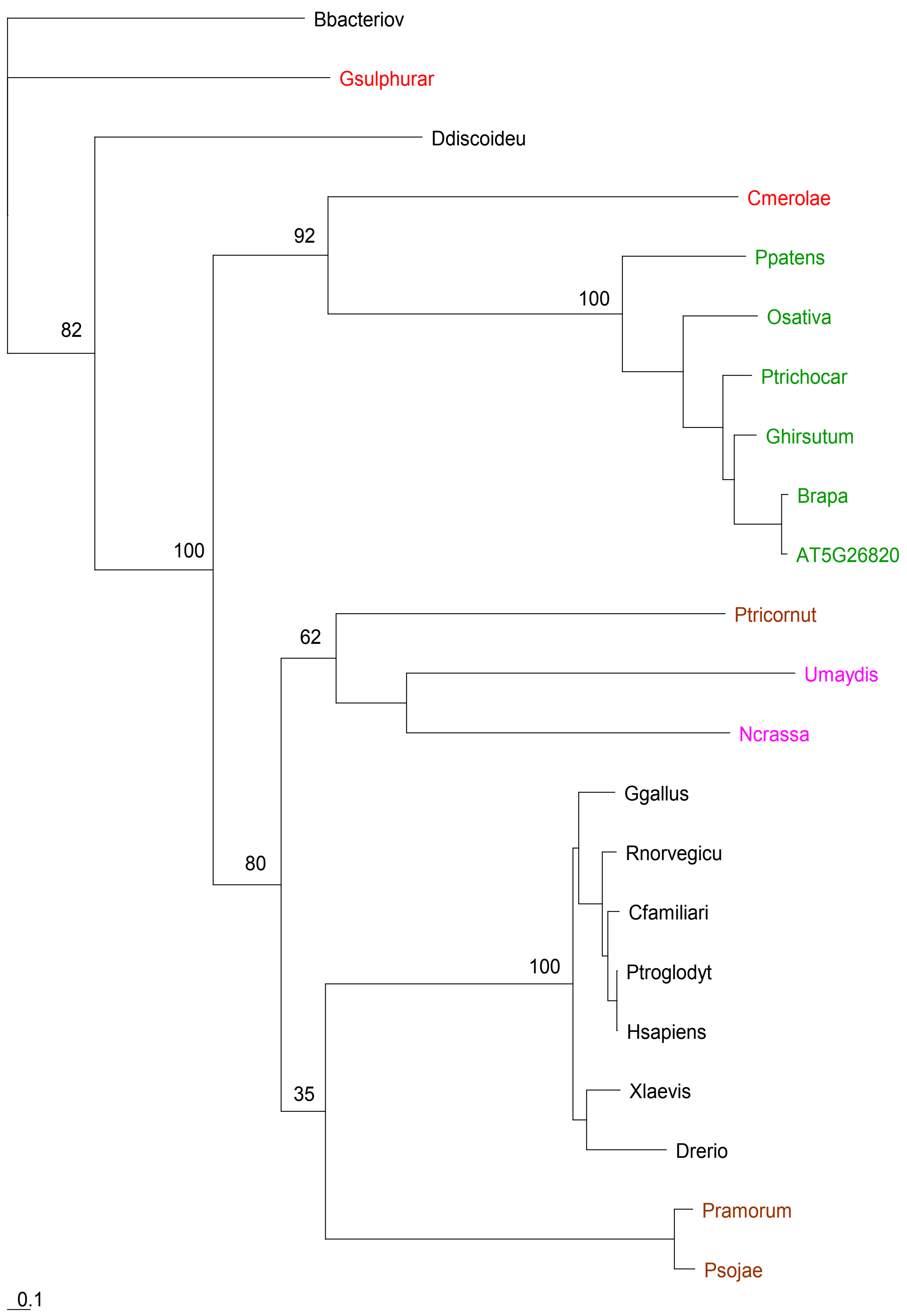

AT5G42130: Mitochondrial Substrate Carrier Family

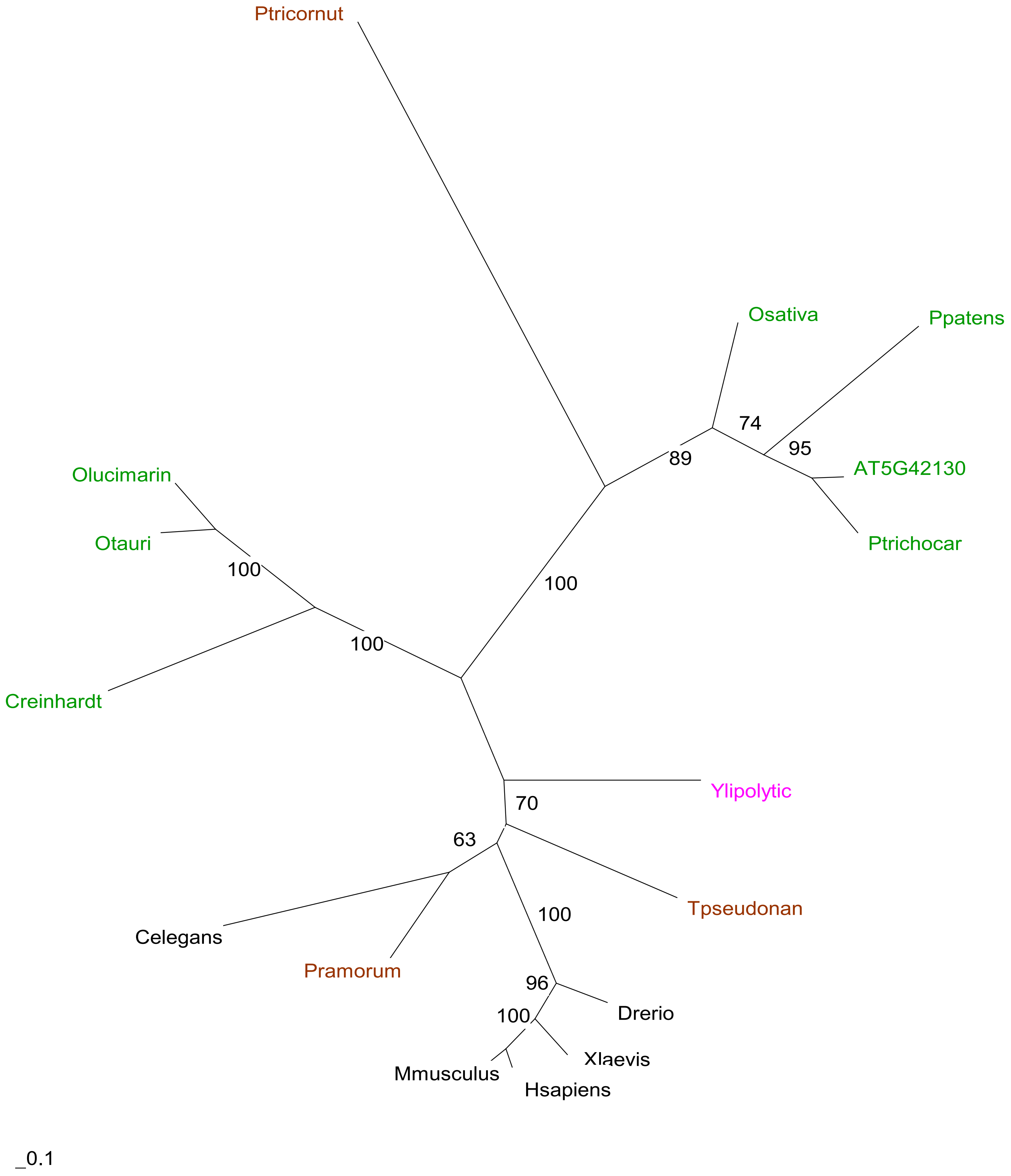

AT5G45450: Iron Transporter

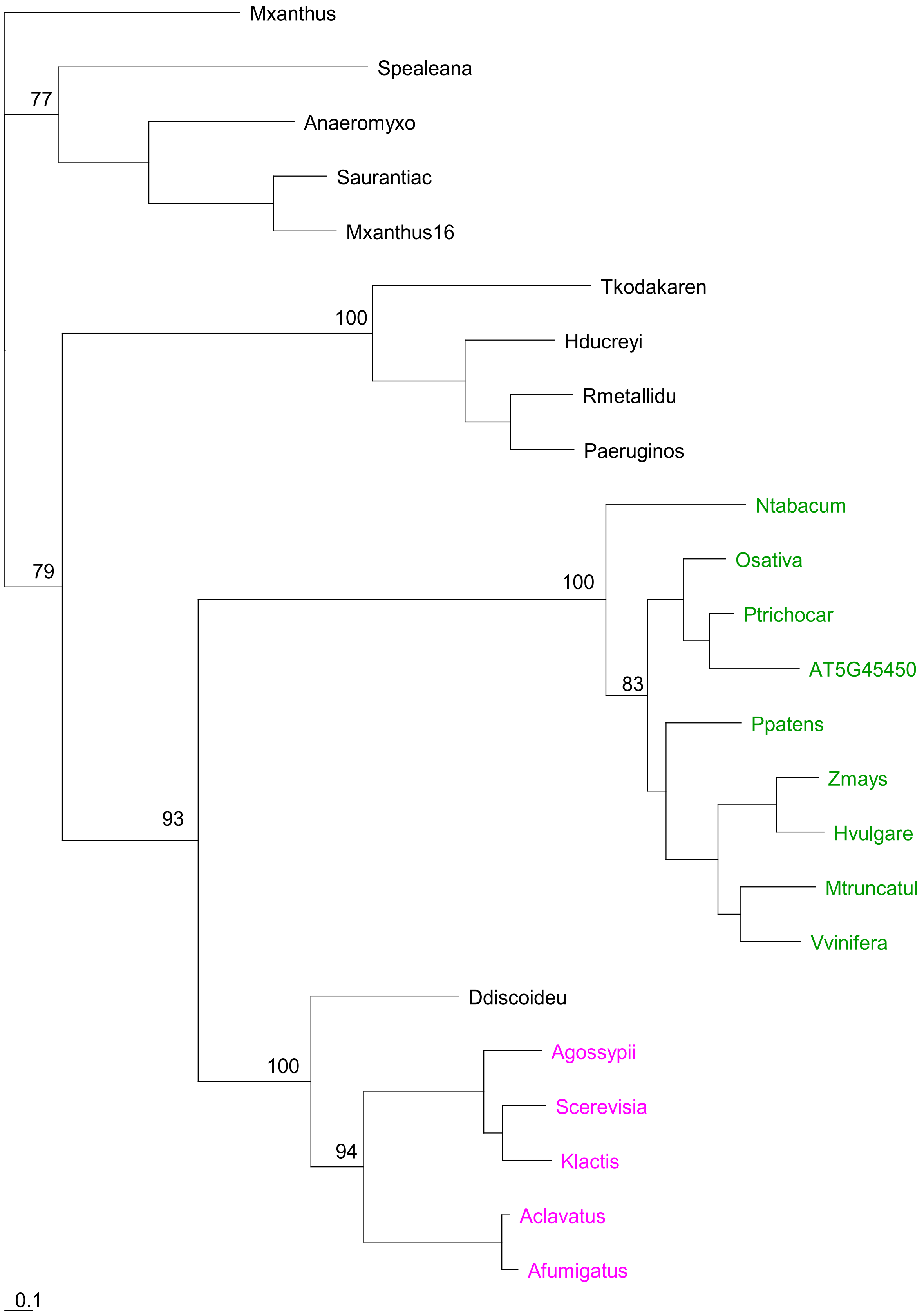

AT5G59250: Sugar Transporter

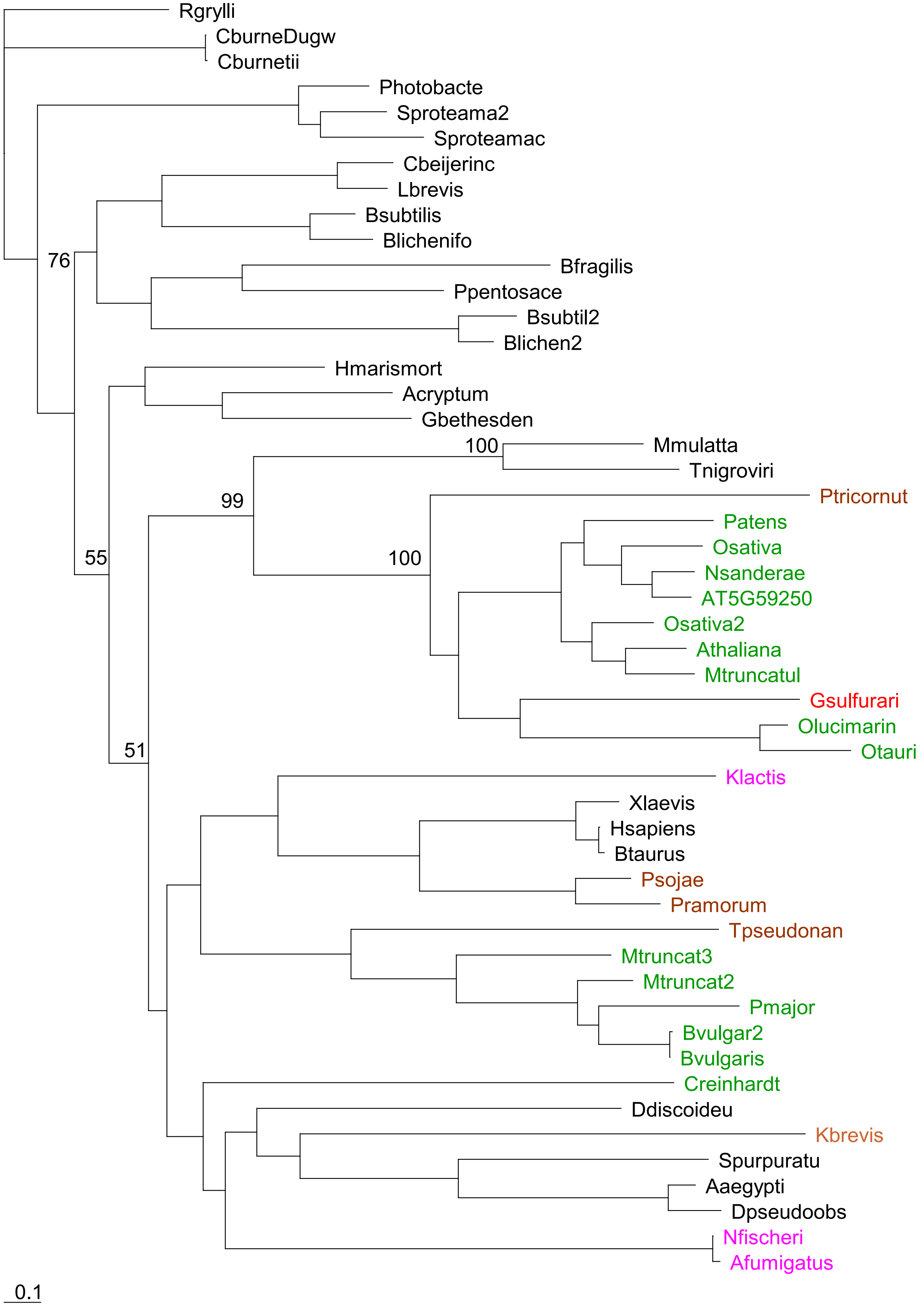

AT5G66380: Folate Carrier

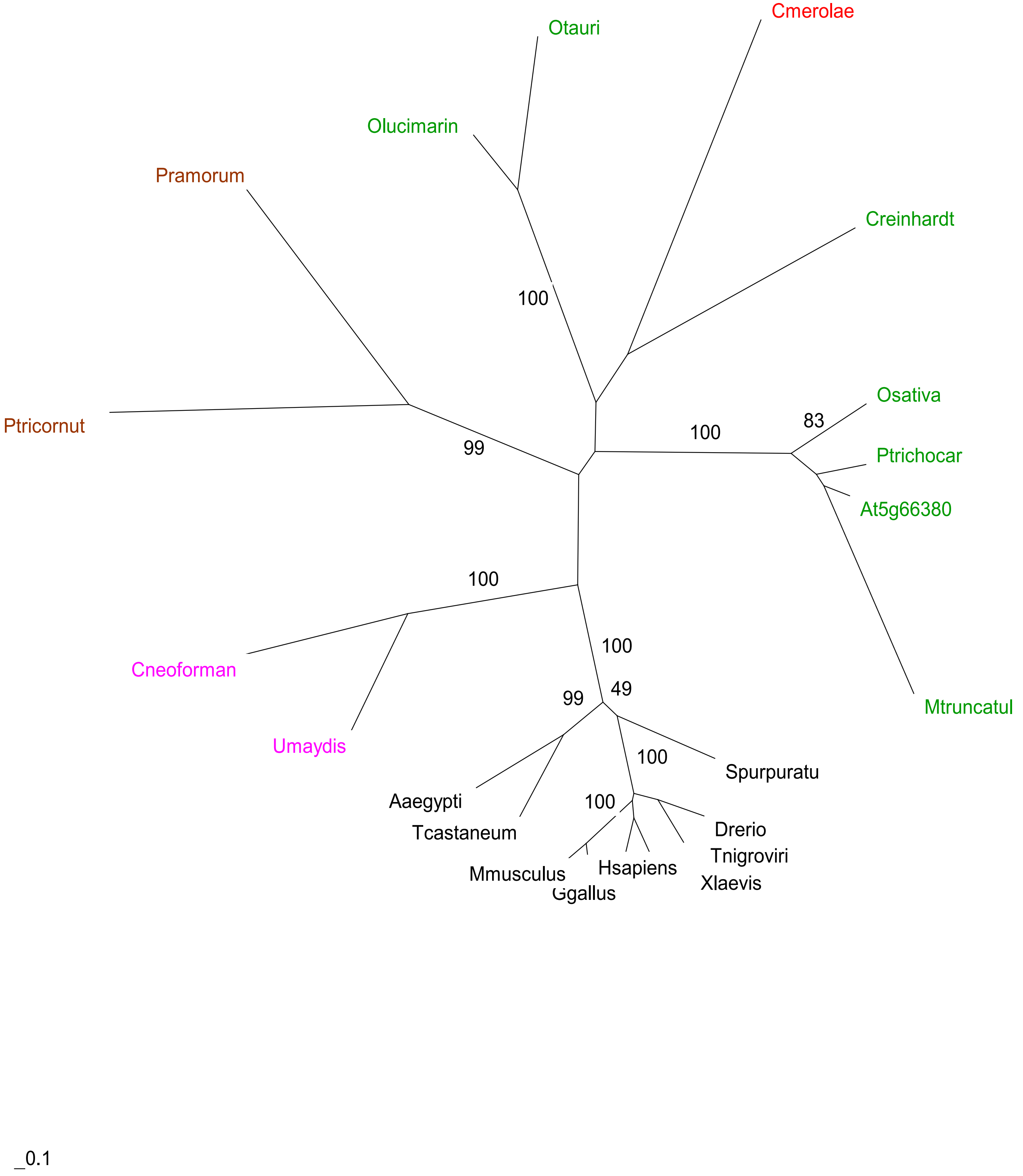

AT5G16150: Hexose Transporter

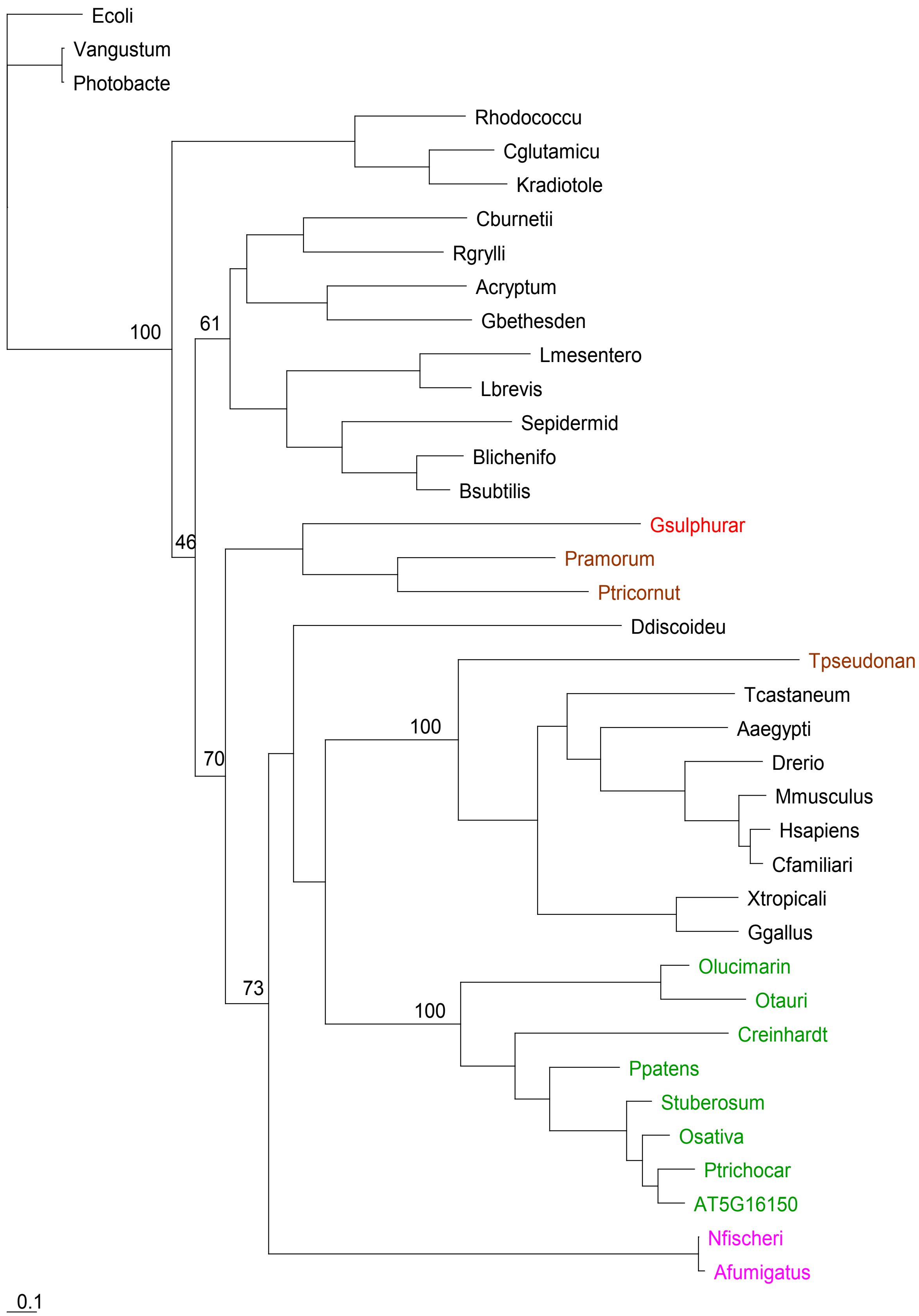

AT1G79450, AT3G12740, AT1G54320: LEM3 Family Proteins

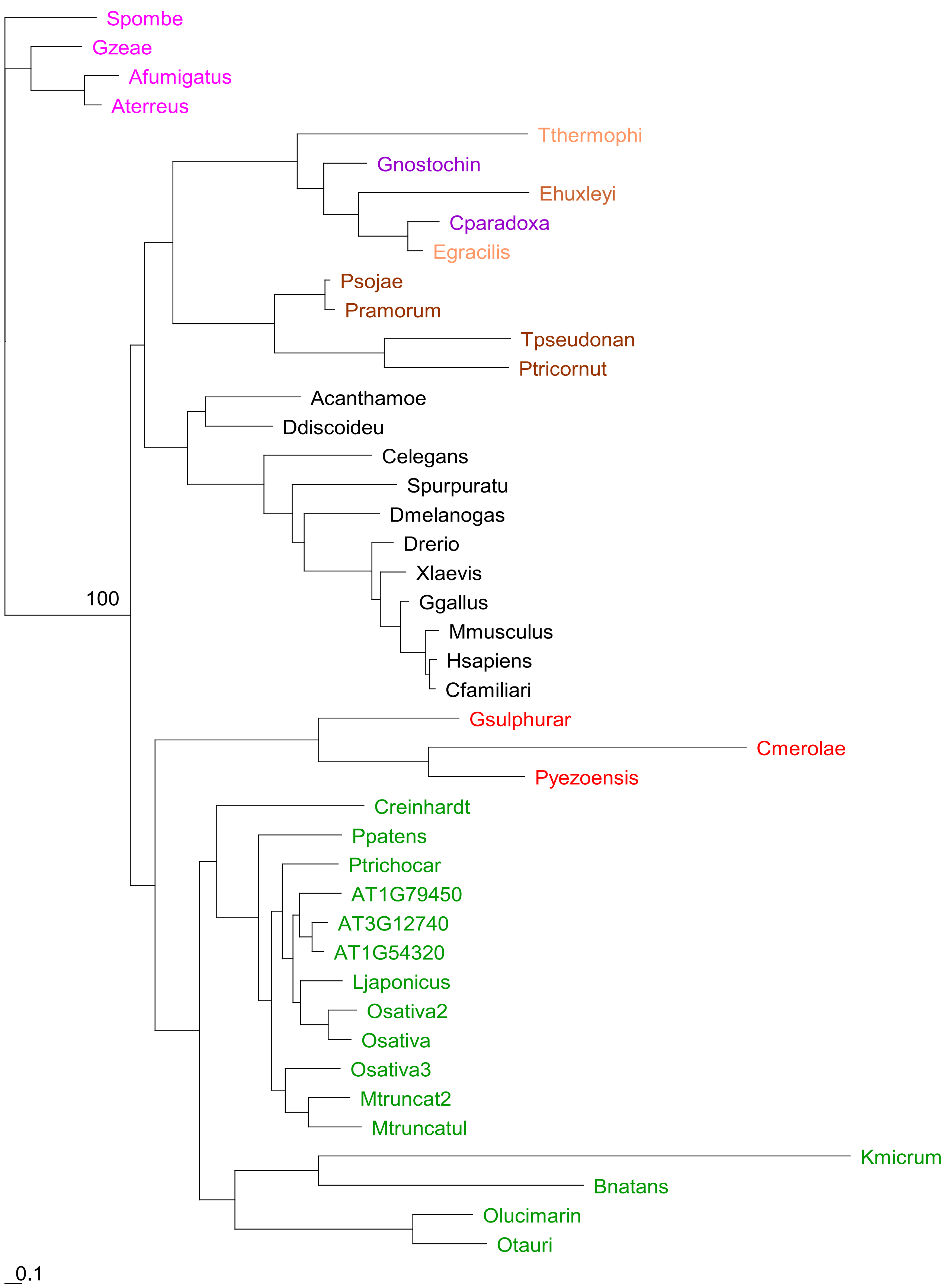

AT2G38330, AT4G38380: MATE Efflux Family

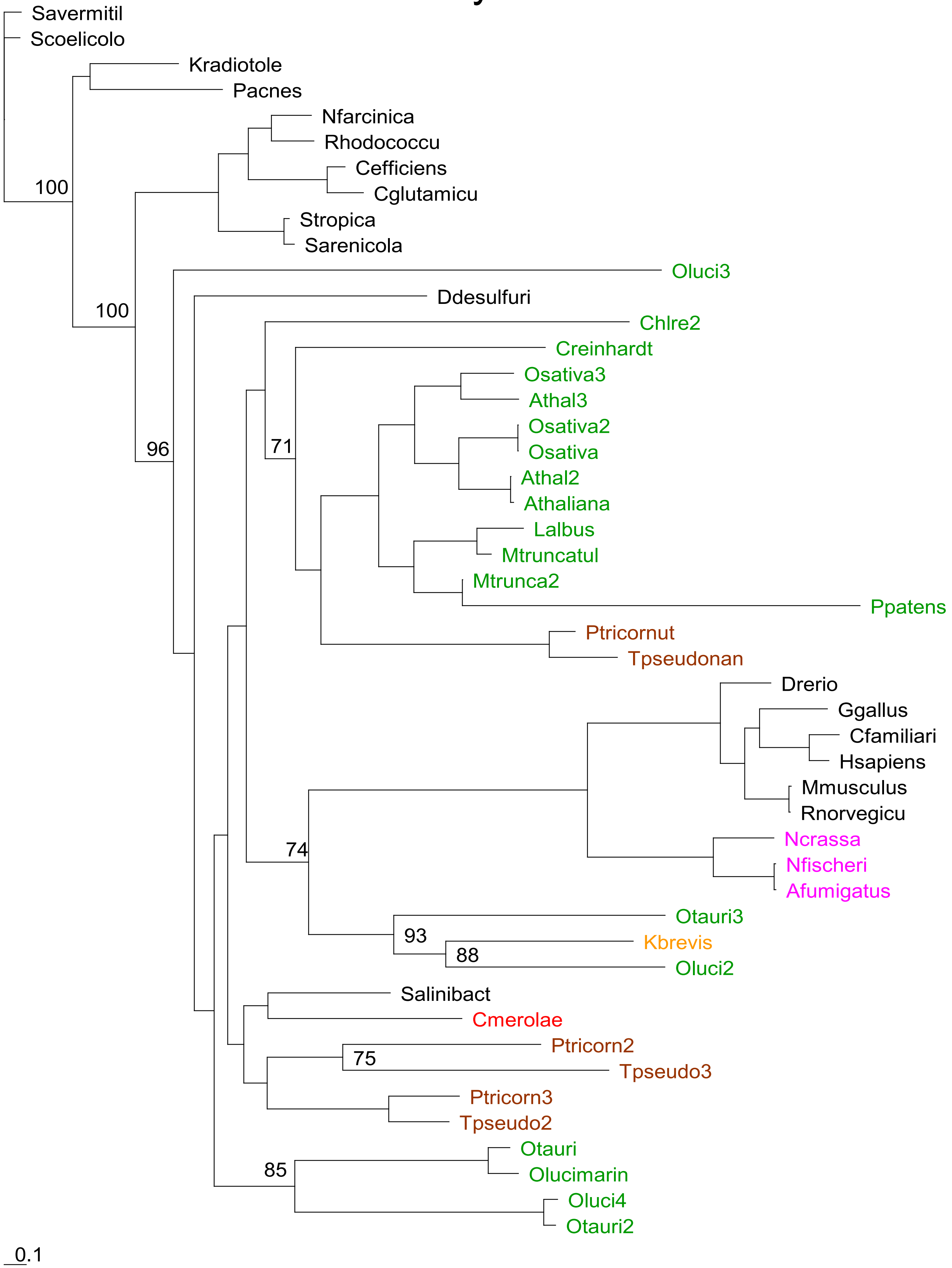

AT1G05580: Sodium/Hydrogen Antiporter

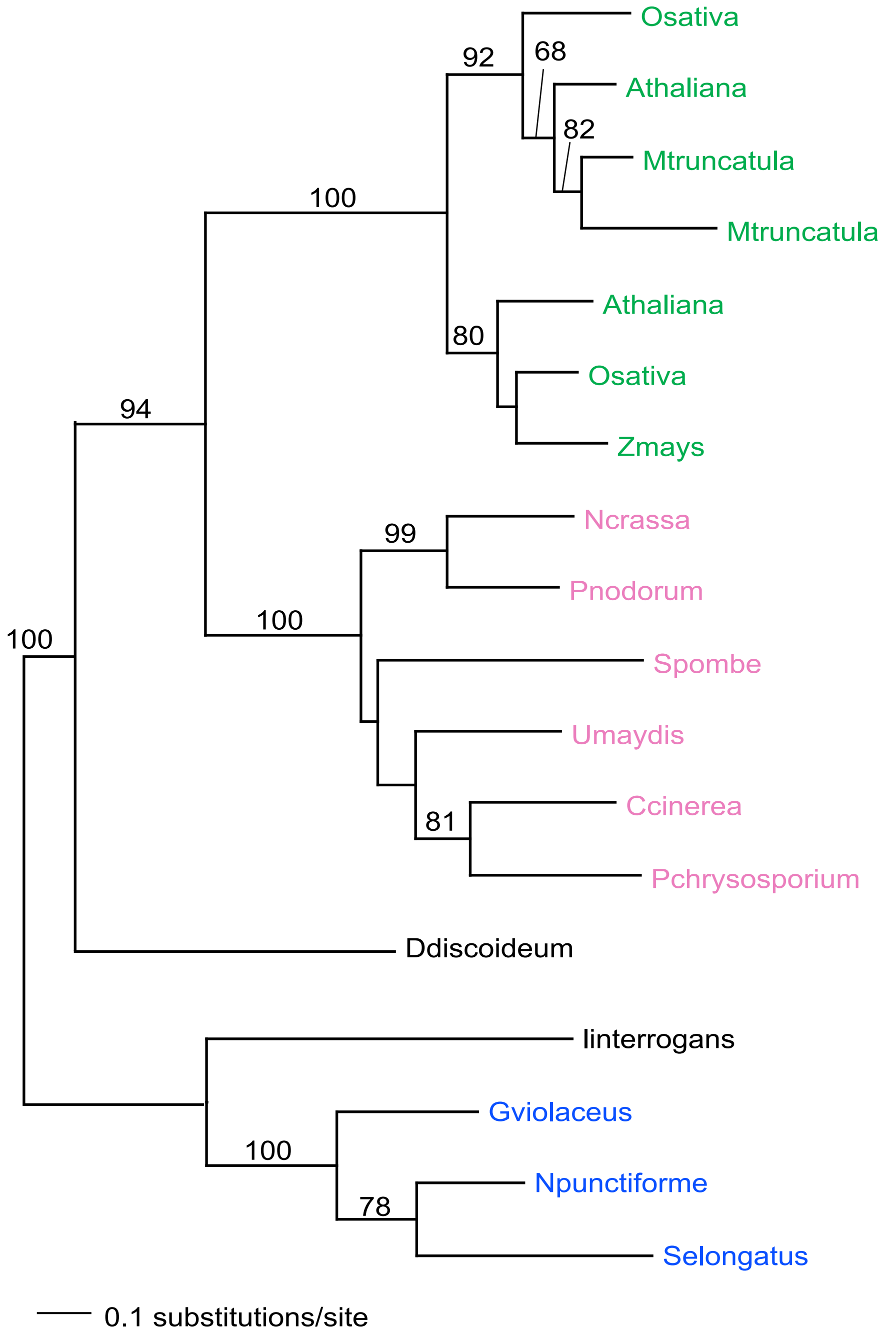

Supplementary Material:

Host Origin of Plastid Solute Transporters in the First Photosynthetic Eukaryotes

Heather M Tyra, Mark Linka, Andreas P.M. Weber, and Debashish Bhattacharya

Figure S2. Plastid targeted solute transporters of putative “Cyanobacterial” (i.e., endosymbiont) origin in Plantae. Ten unique genes fall in this category. These are PHYML trees with the numbers above the branches inferred from a PHYML bootstrap analysis. Only bootstrap values ≥ 60% are shown. Branch lengths are proportional to the number of substitutions per site (see scale bars). The different photosynthetic groups are shown in different text colors: blue for cyanobacteria, red for red algae, green for green algae and land plants, magenta for glaucophytes, and brown for chromalveolates. The inclusion of chromalveolates within the Plantae is believed to reflect horizontal or endosymbiotic gene transfer events (e.g., Li et al. 2006).

AT5G64940: ABC Transporter

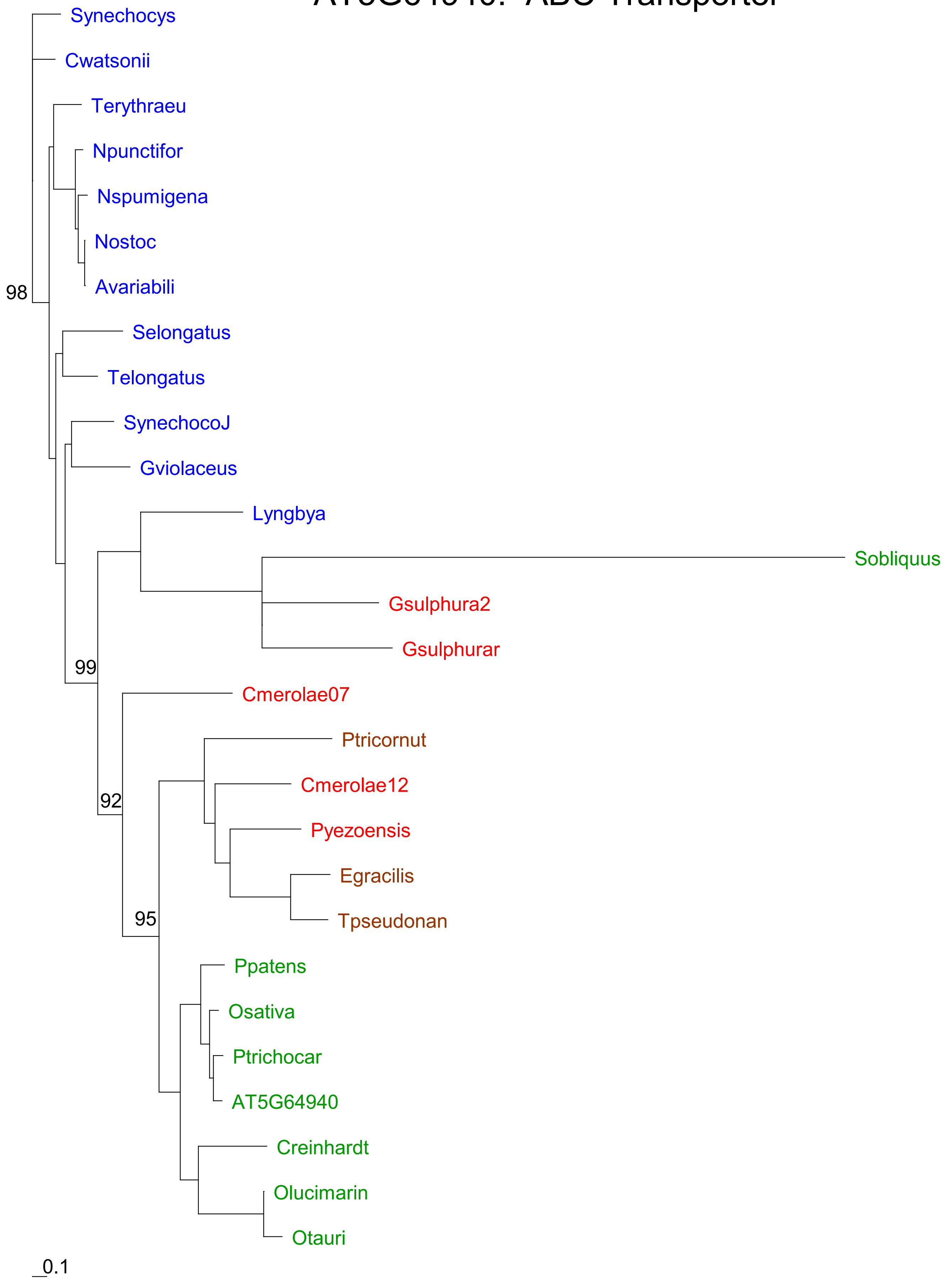

AT1G08640: Expressed Protein

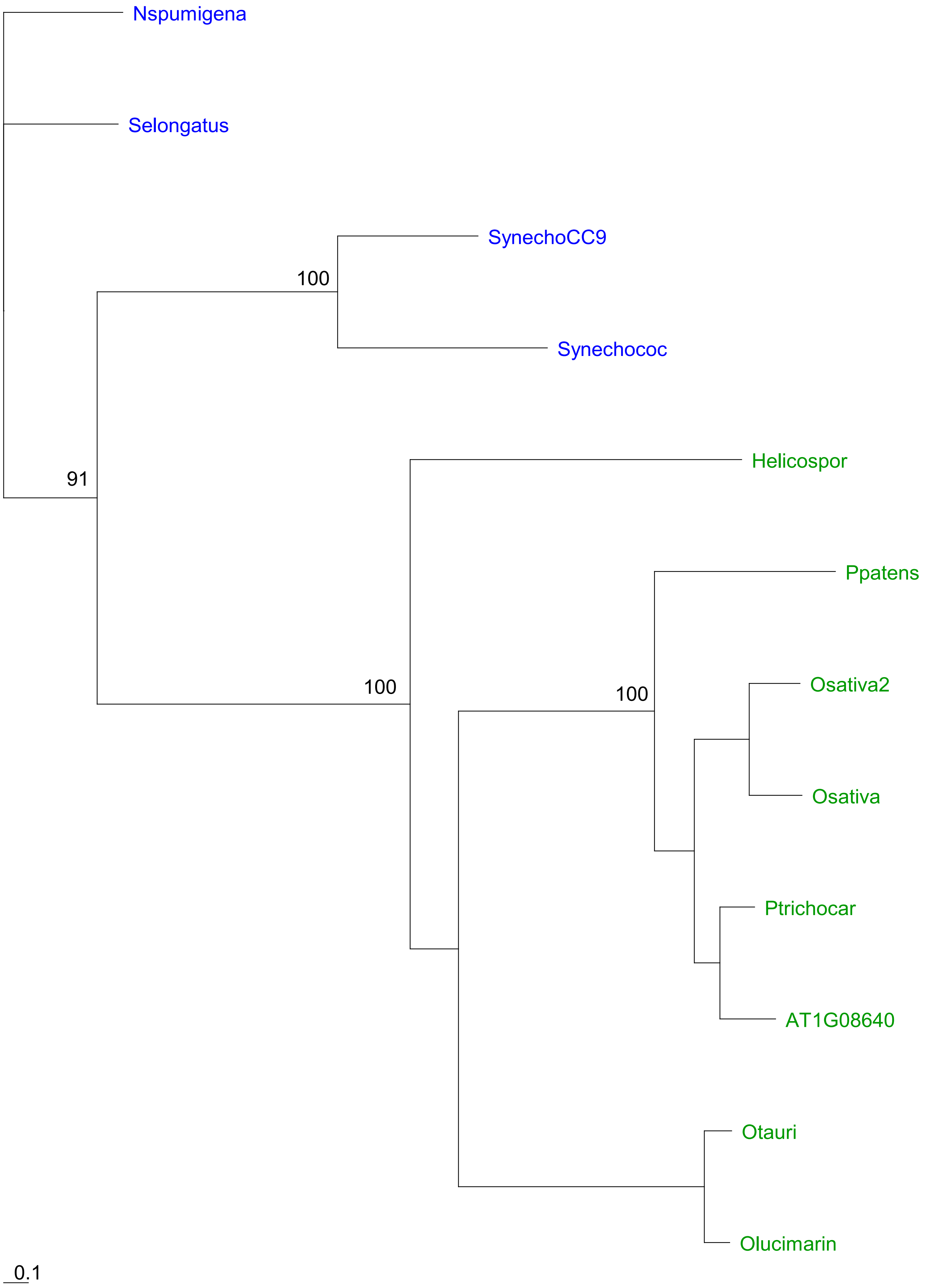

AT1G19800: TGD1

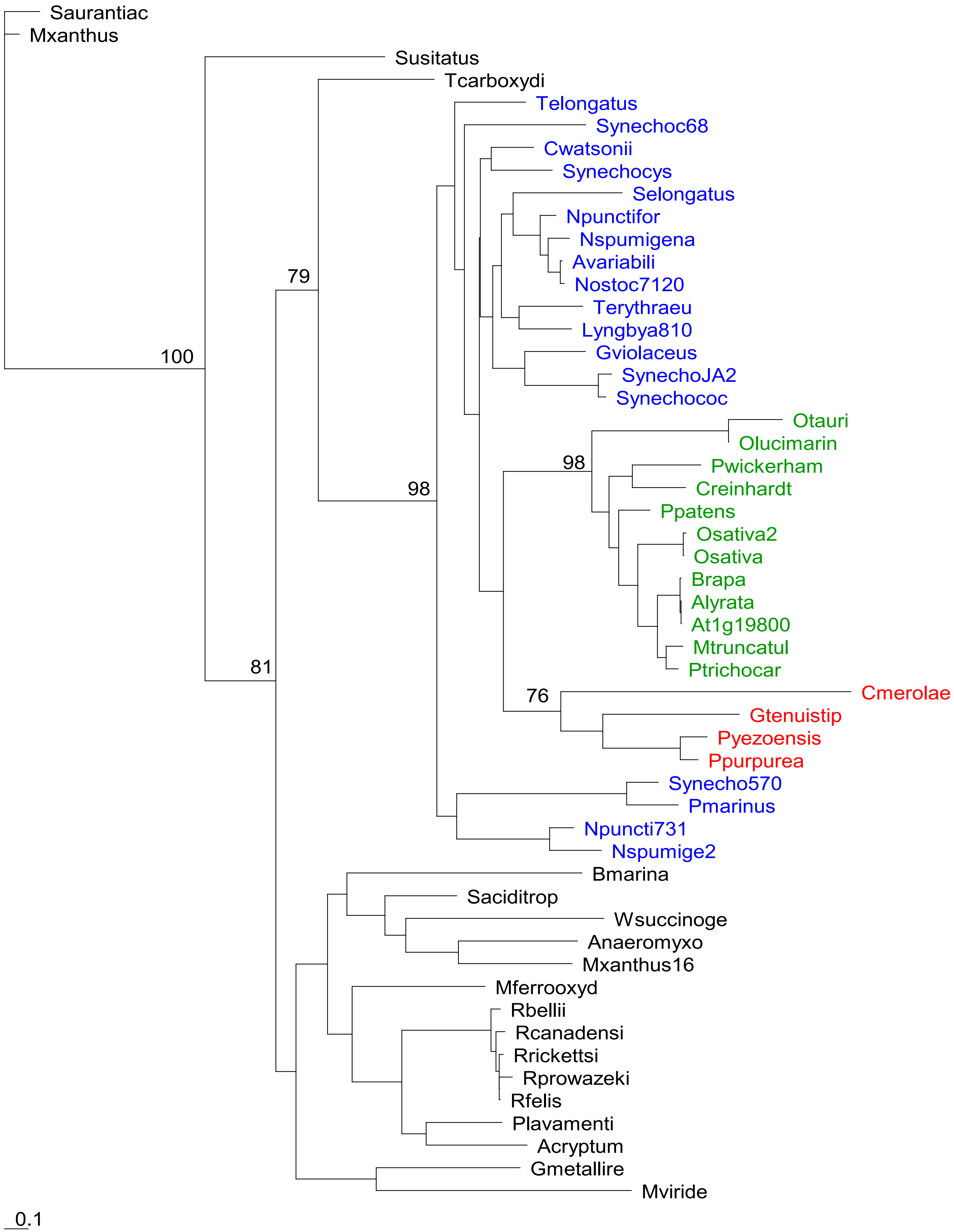

AT1G78620: Integral Membrane Family Protein

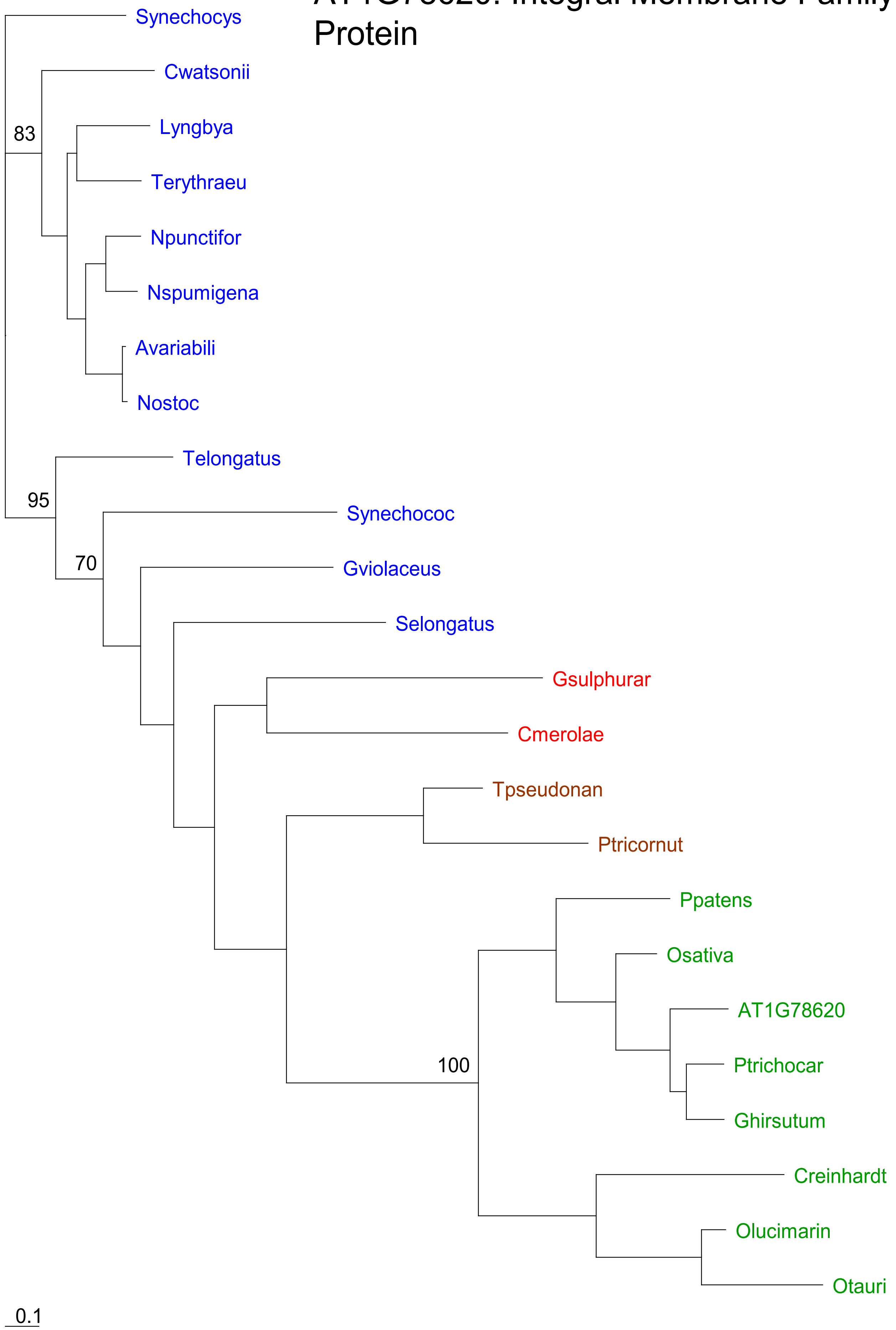

AT2G32040: Folate Monoglutamate Transporter

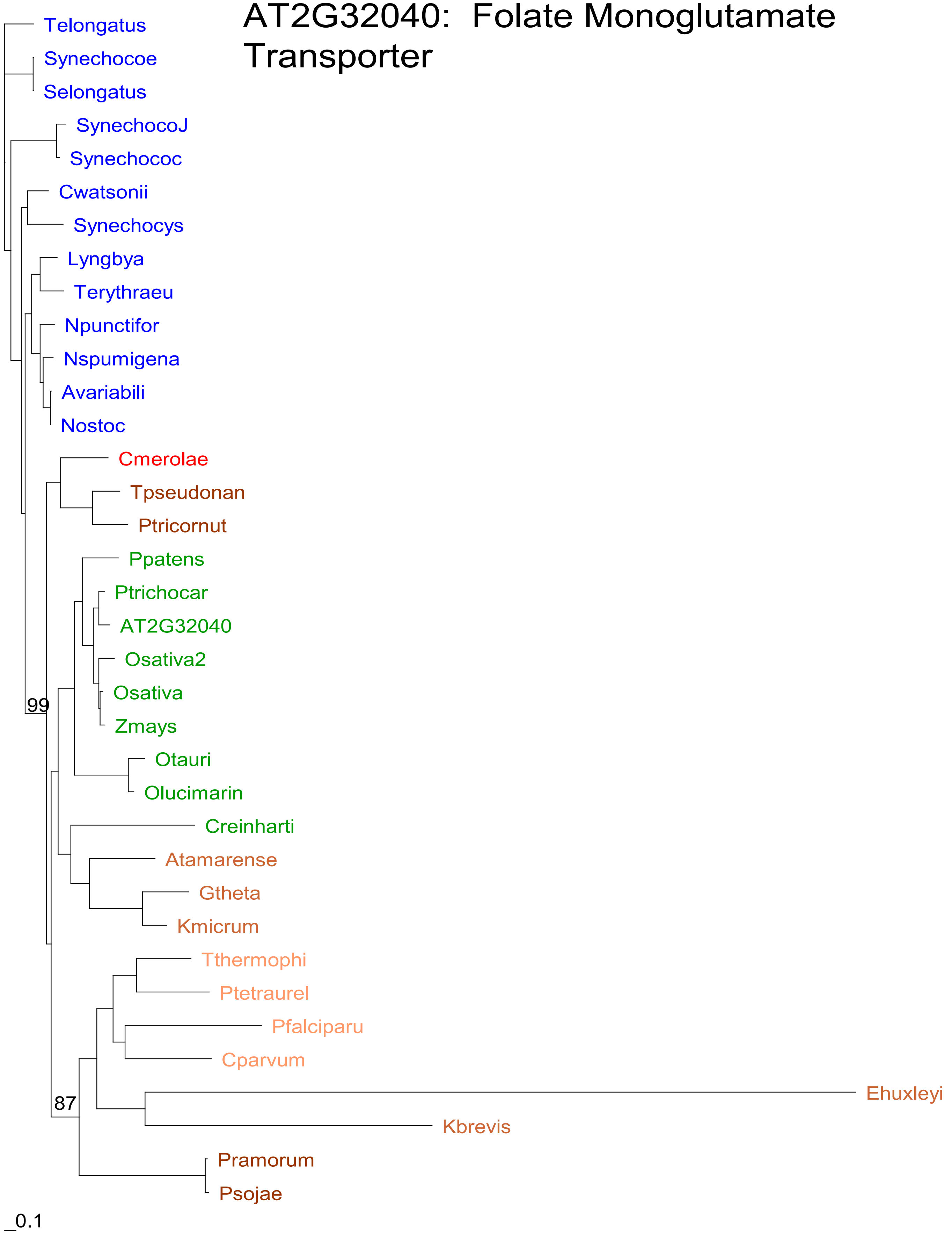

AT3G51140: Expressed Protein

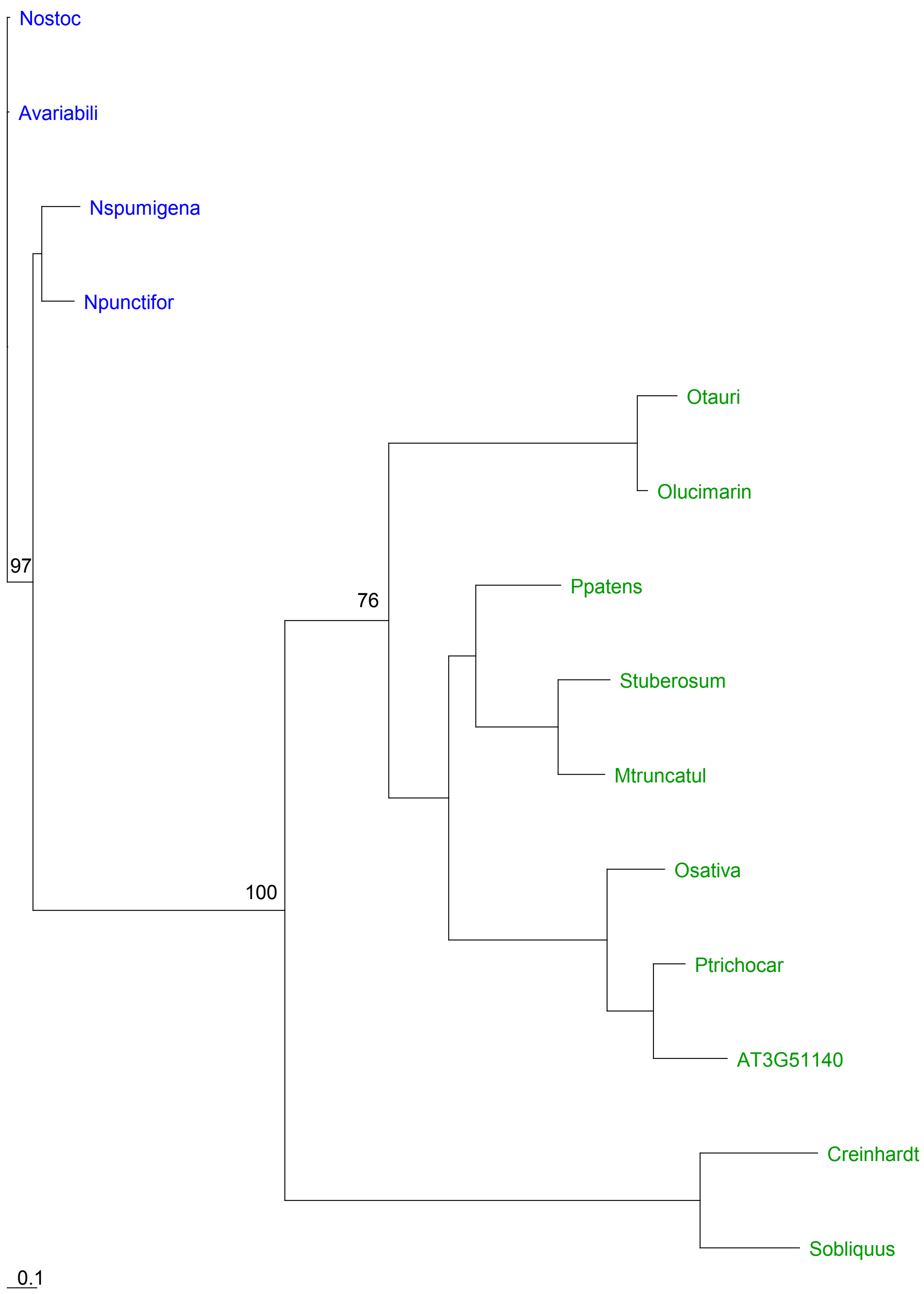

AT3G60590: Expressed Protein

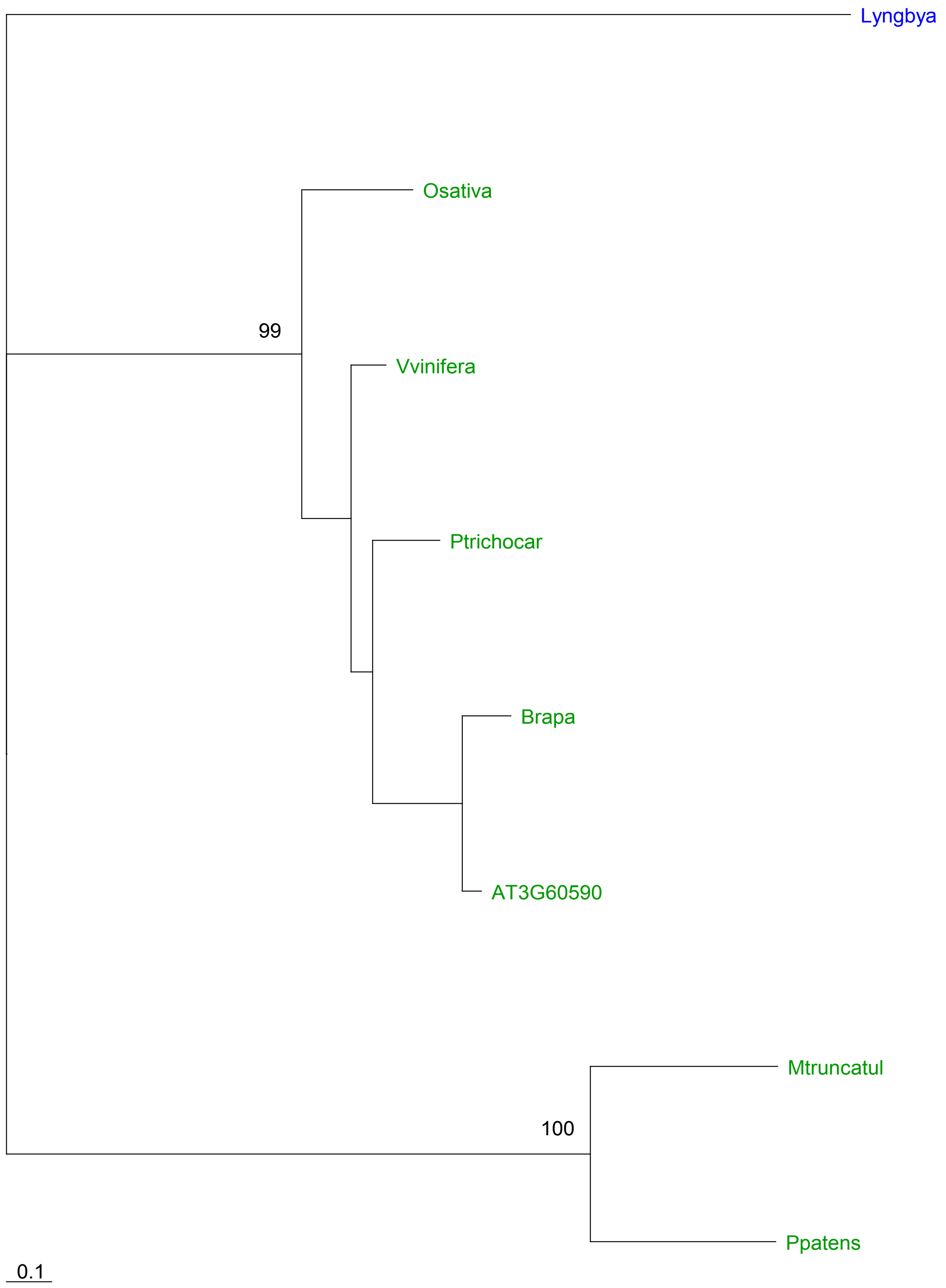

At4g33520: PAA1 and PAA2

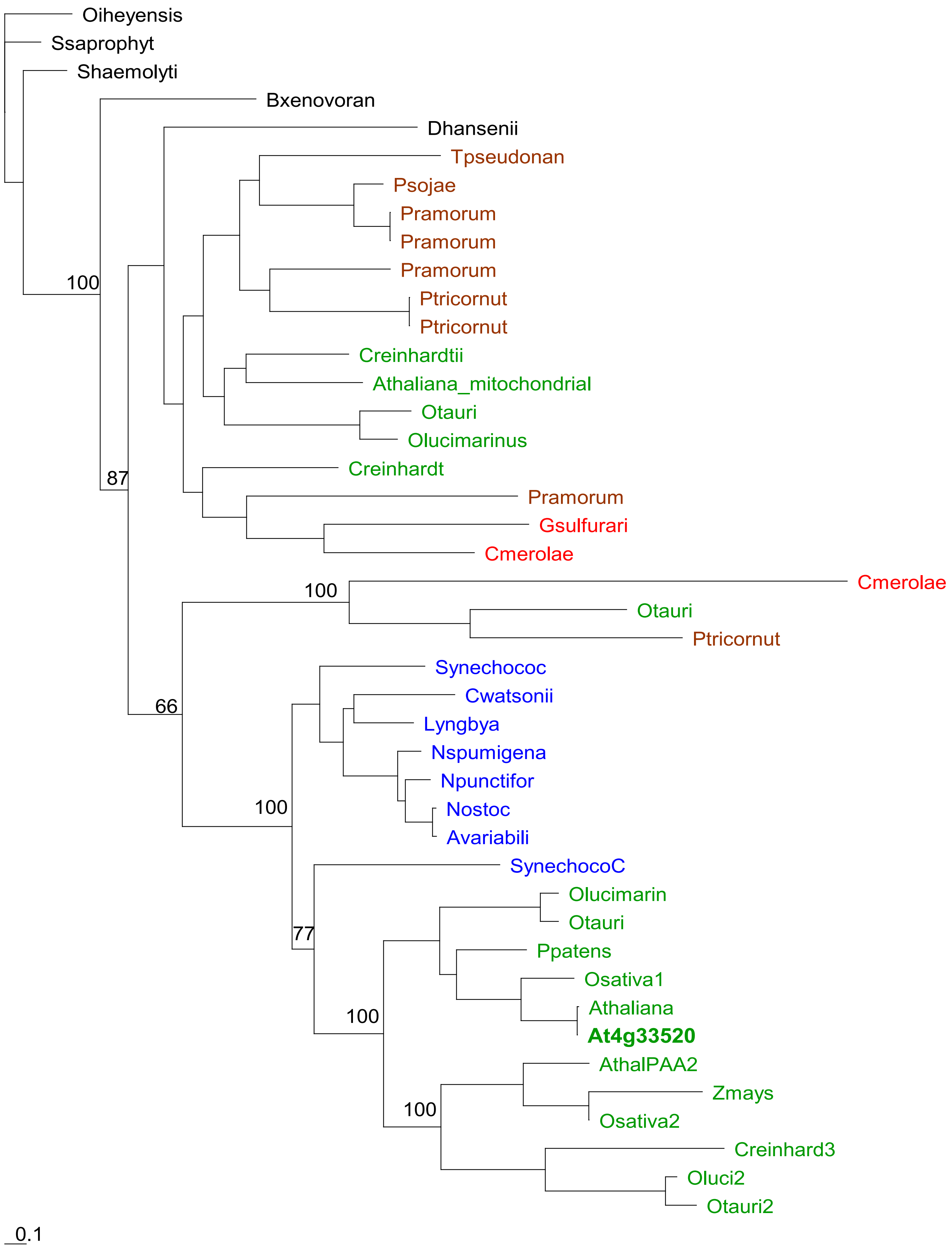

AT5G12470: Expressed Protein

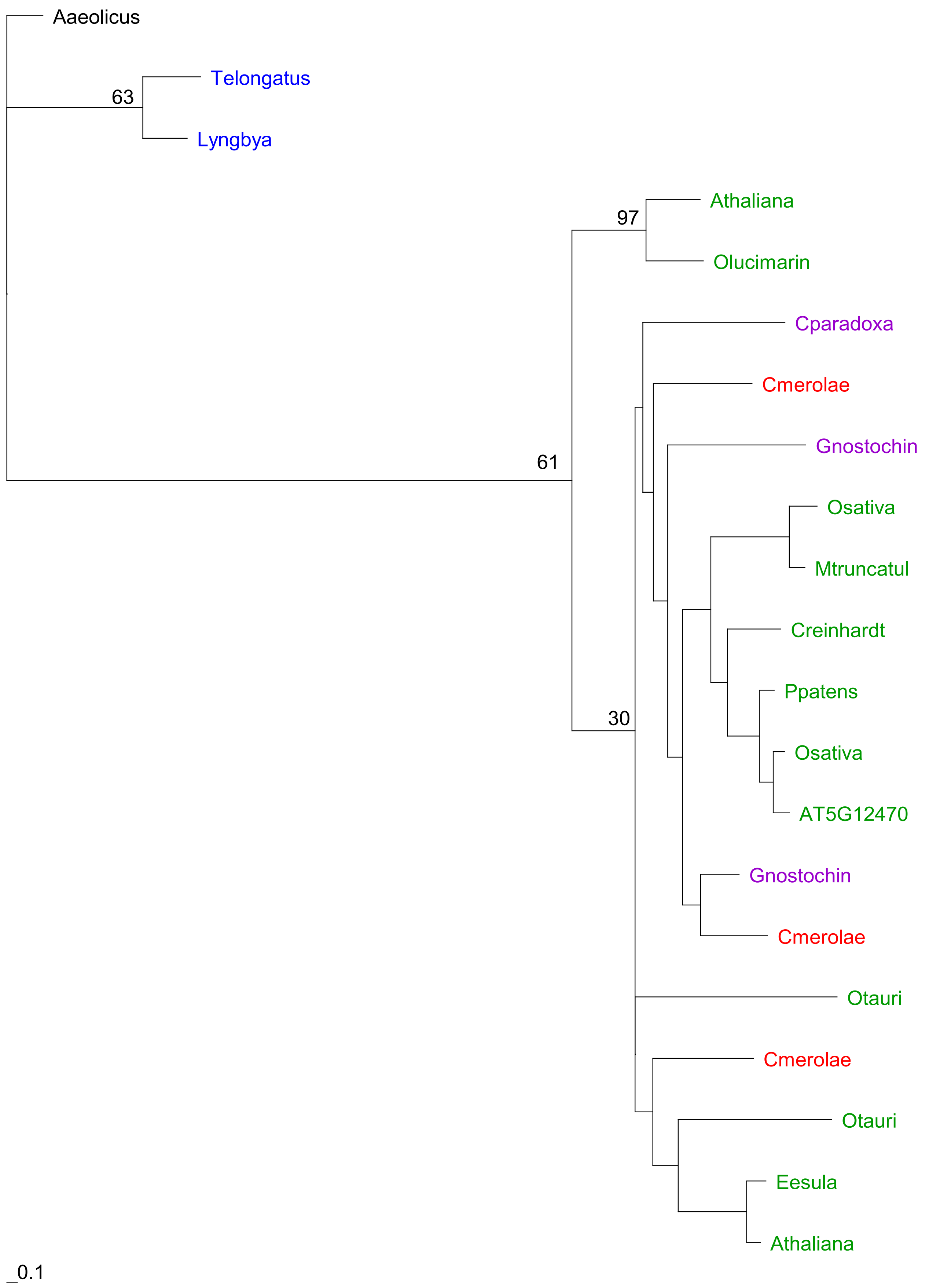

AT1G04570: Integral Membrane Family Protein

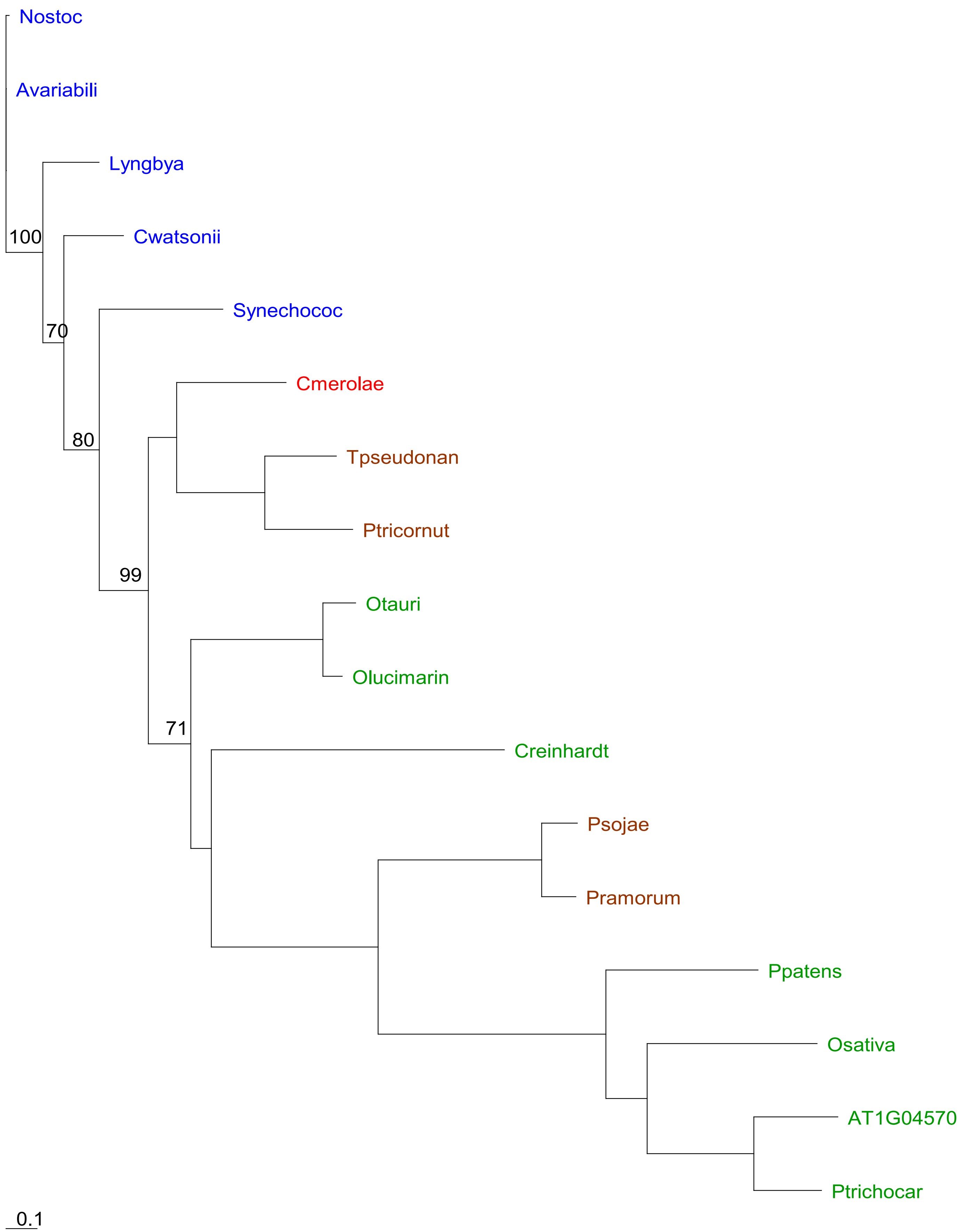

Supplementary Material:

Host Origin of Plastid Solute Transporters in the First Photosynthetic Eukaryotes

Heather M Tyra, Mark Linka, Andreas P.M. Weber, and Debashish Bhattacharya

Figure S3. Plastid targeted solute transporters of putative "*Chlamydia*-like" origin in Plantae. Four unique genes fall in this category of which two are shown here, the other two are in the main text: (A) Dicarboxylate translocators DiT1, DiT2.1, and DiT2.2 and (B) Phosphate transporter PHT2;1. These are PHYML trees with the numbers above the branches inferred from a PHYML bootstrap analysis. Only bootstrap values ≥ 60% are shown. Branch lengths are proportional to the number of substitutions per site (see scale bars). The thick branches show significant (P > 0.95) support from a Bayesian phylogenetic inference. The filled magenta circle shows the node that unites chlamydial taxa with plastid targeted Plantae transporters. The different photosynthetic groups are shown in different text colors: blue for cyanobacteria, red for red algae, green for green algae and land plants, magenta for glaucophytes, and brown for chromalveolates. The inclusion of chromalveolates within the Plantae is believed to reflect horizontal or endosymbiotic gene transfer events (e.g., Li et al. 2006).

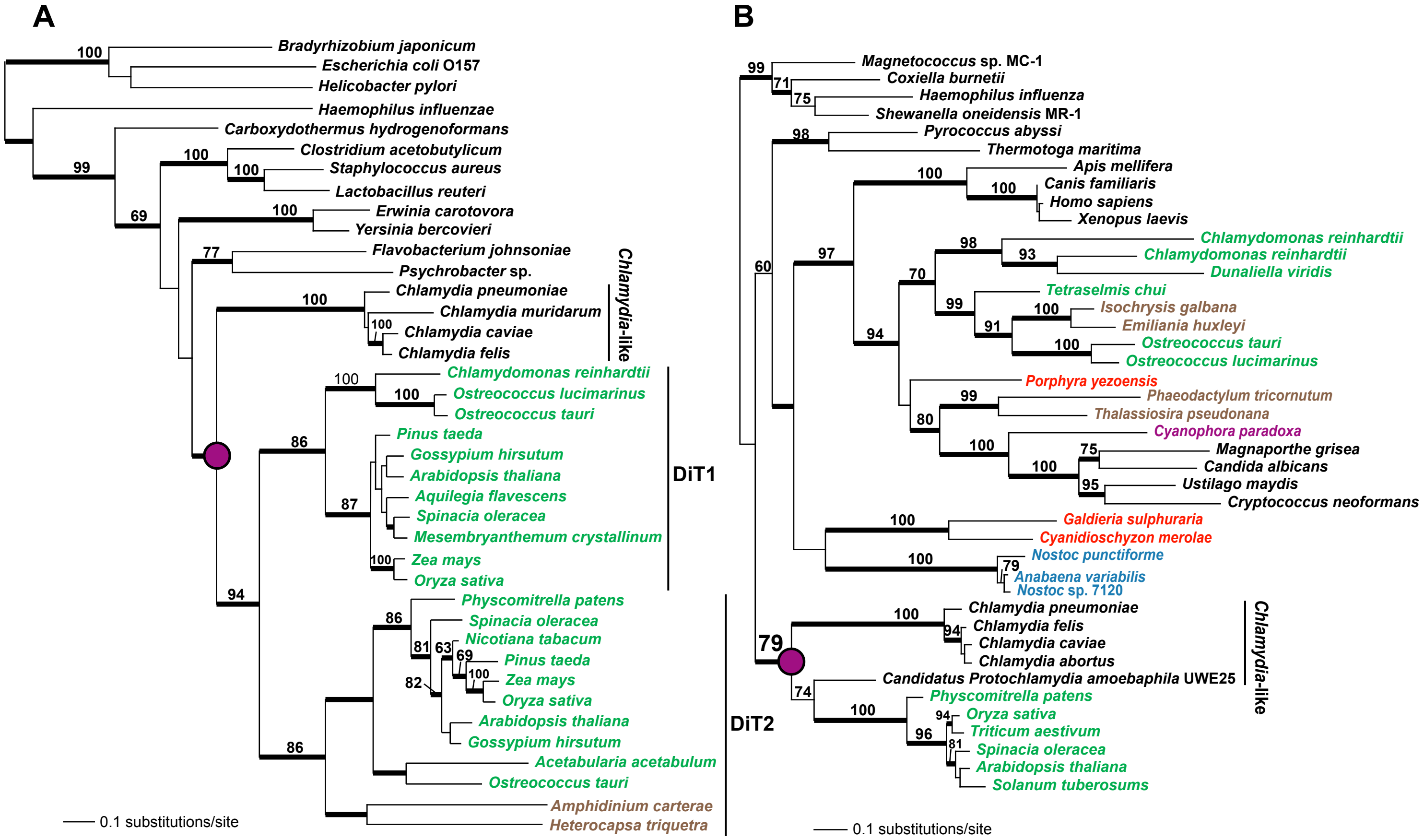
A
Bradyrhizobium japonicum
Escherichia coli O157
Helicobacter pylori
Haemophilus influenzae
Carboxydothermus hydrogenoformans
Clostridium acetobutylicum
Staphylococcus aureus
Lactobacillus reuteri
Erwinia carotovora
Yersinia bercovieri
Flavobacterium johnsoniae
Psychrobacter sp.
Chlamydia pneumoniae
Chlamydia muridarum
Chlamydia caviae
Chlamydia felis
Chlamydia-like
Chlamydomonas reinhardtii
Ostreococcus lucimarinus
Ostreococcus tauri
Pinus taeda
Gossypium hirsutum
Arabidopsis thaliana
Aquilegia flavescens
Spinacia oleracea
Mesembryanthemum crystallinum
Zea mays
Oryza sativa
DiT1
Physcomitrella patens
Spinacia oleracea
Nicotiana tabacum
Pinus taeda
Zea mays
Oryza sativa
Arabidopsis thaliana
Gossypium hirsutum
Acetabularia acetabulum
Ostreococcus tauri
Amphidinium carterae
Heterocapsa triquetra
DiT2
0.1 substitutions/site
B
Magnetococcus sp. MC-1
Coxiella burnetii
Haemophilus influenza
Shewanella oneidensis MR-1
Pyrococcus abyssi
Thermotoga maritima
Apis mellifera
Canis familiaris
Homo sapiens
Xenopus laevis
Chlamydomonas reinhardtii
Chlamydomonas reinhardtii
Dunaliella viridis
Tetraselmis chui
Isochrysis galbana
Emiliania huxleyi
Ostreococcus tauri
Ostreococcus lucimarinus
Porphyra yezoensis
Phaeodactylum tricornutum
Thalassiosira pseudonana
Cyanophora paradoxa
Magnaporthe grisea
Candida albicans
Ustilago maydis
Cryptococcus neoformans
Galdieria sulphuraria
Cyanidioschyzon merolae
Nostoc punctiforme
Anabaena variabilis
Nostoc sp. 7120
Chlamydia pneumoniae
Chlamydia felis
Chlamydia caviae
Chlamydia abortus
Candidatus Protochlamydia amoebaphila UWE25
Chlamydia-like
Physcomitrella patens
Oryza sativa
Triticum aestivum
Spinacia oleracea
Arabidopsis thaliana
Solanum tuberosums
0.1 substitutions/site

Supplementary Material:

Host Origin of Plastid Solute Transporters in the First Photosynthetic Eukaryotes

Heather M Tyra, Mark Linka, Andreas P.M. Weber, and Debashish Bhattacharya

Figure S4. Plastid targeted solute transporters of putative “Other” origin in Plantae. Twelve unique genes fall in this category. These are PHYML trees with the numbers above the branches inferred from a PHYML bootstrap analysis. Only bootstrap values ≥ 60% are shown. Branch lengths are proportional to the number of substitutions per site (see scale bars). The different photosynthetic groups are shown in different text colors: blue for cyanobacteria, red for red algae, green for green algae and land plants, and brown for chromalveolates. In addition, the fungi are shown in pink text and *Chlamydia*-like taxa in magenta. The inclusion of chromalveolates within the Plantae is believed to reflect horizontal or endosymbiotic gene transfer events (e.g., Li et al. 2006).

AT5G52540: Expressed Protein

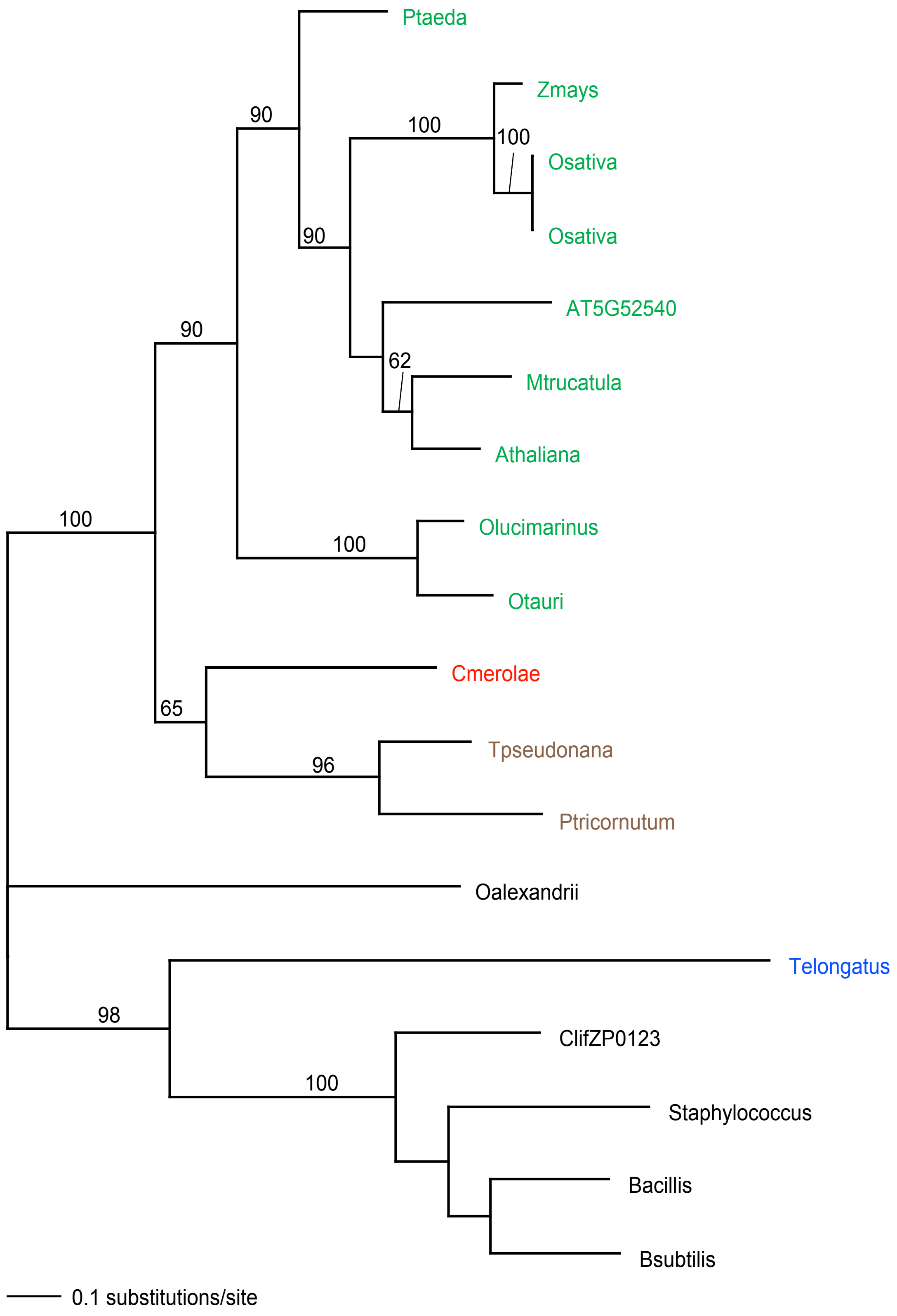

AT1G01790: Potassium Antiporter

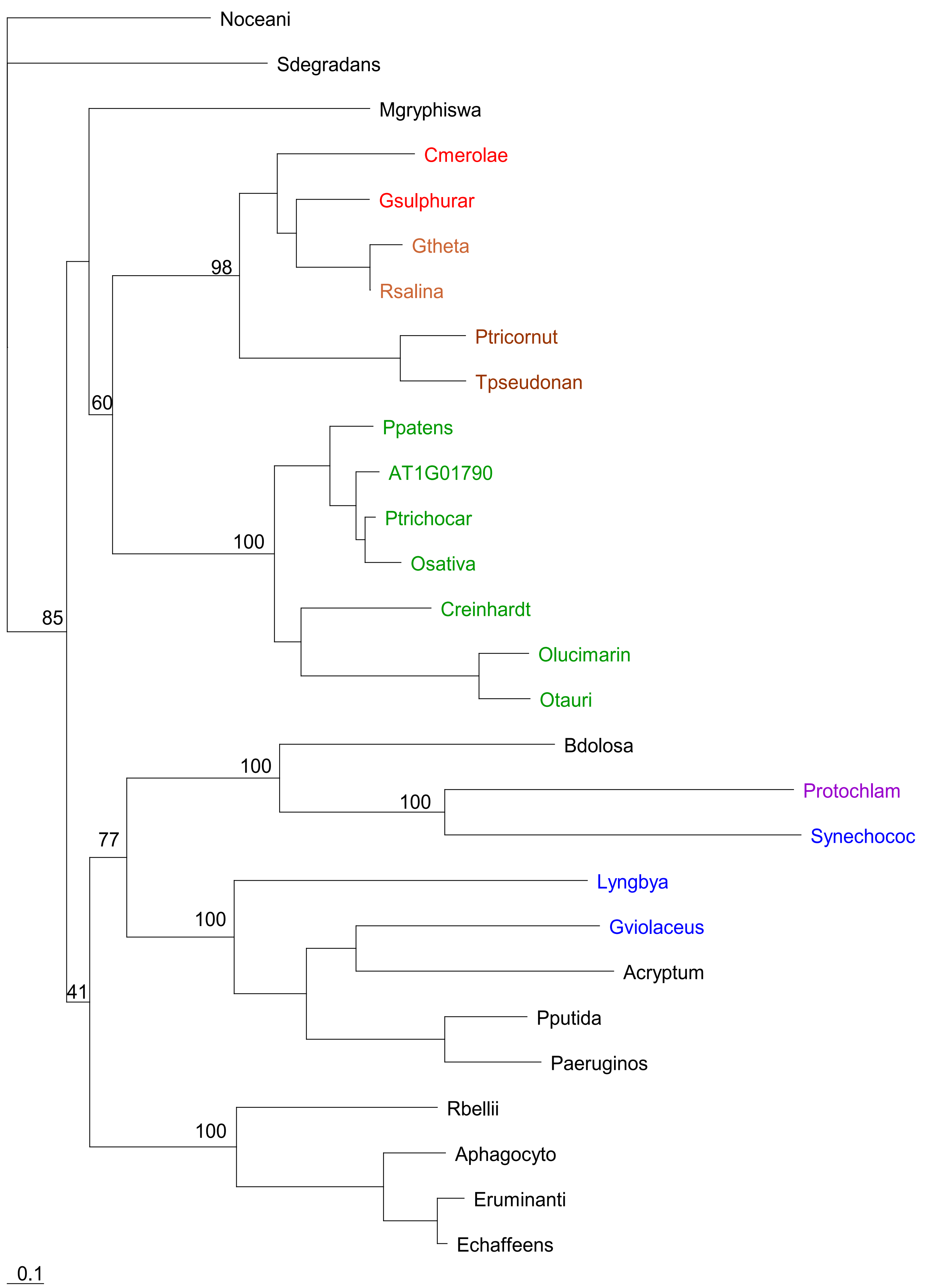

AT1G32080: Membrane Protein

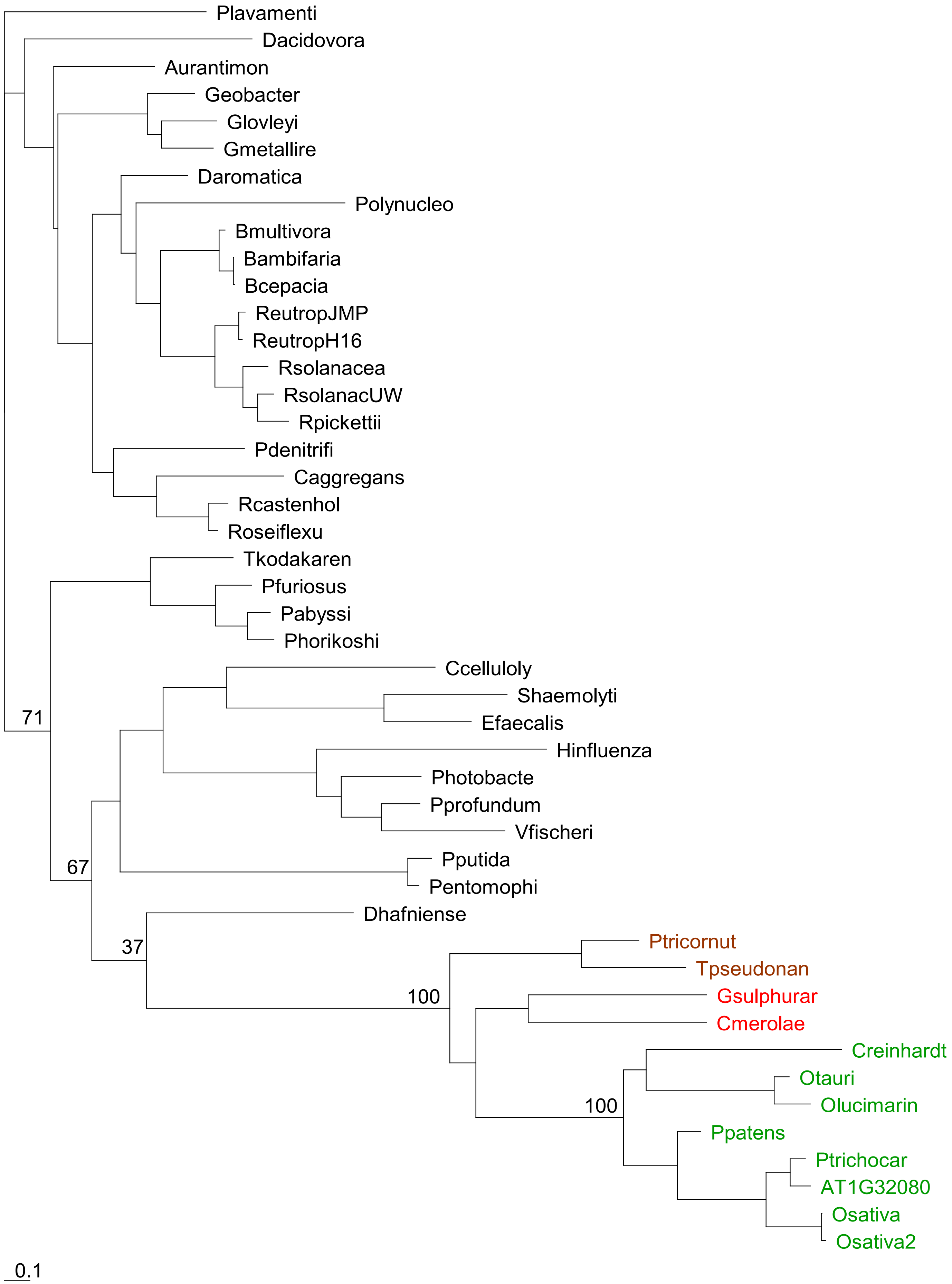

AT1G44920: Expressed Protein

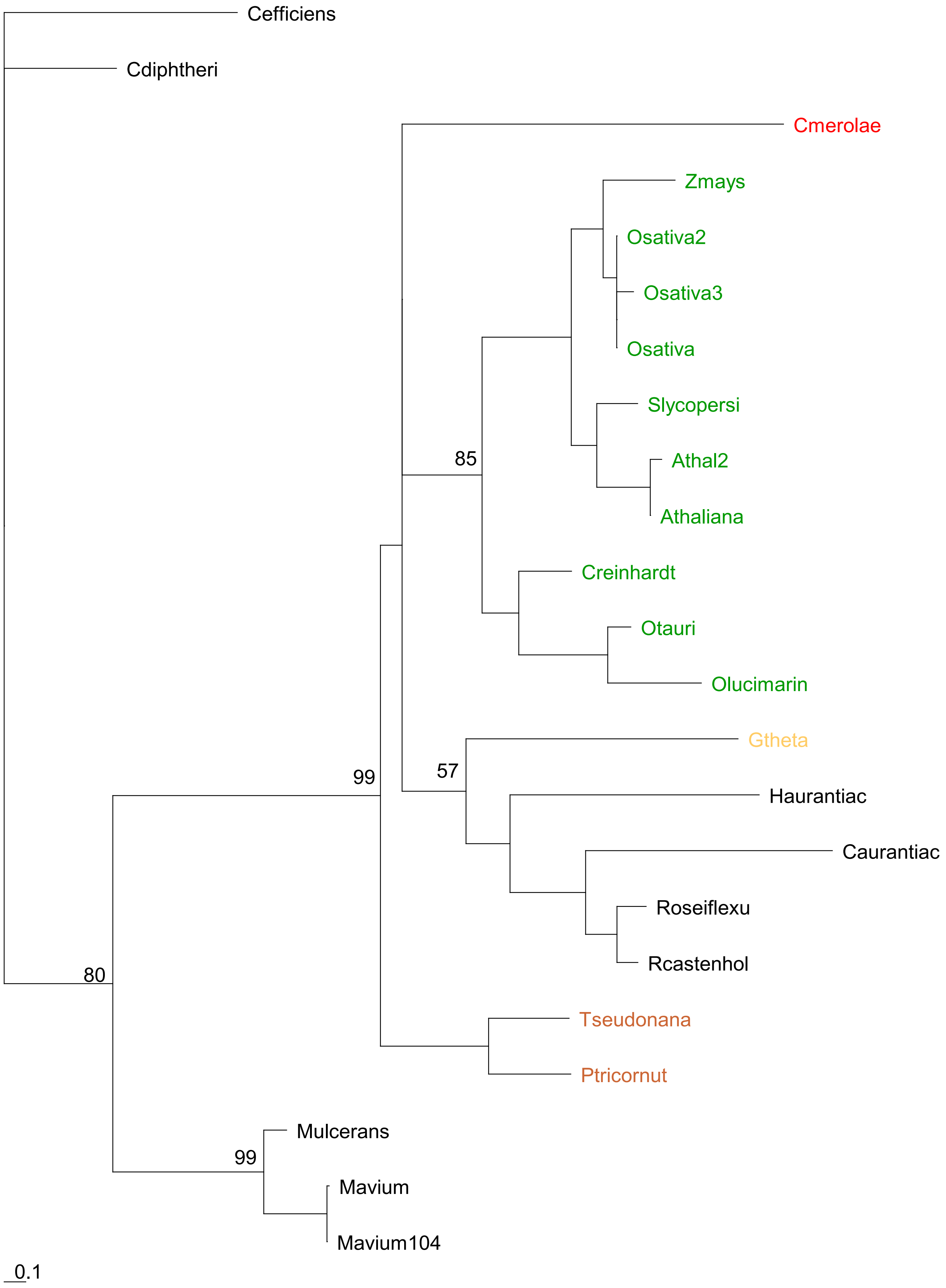

AT1G54350: ABC Transporter

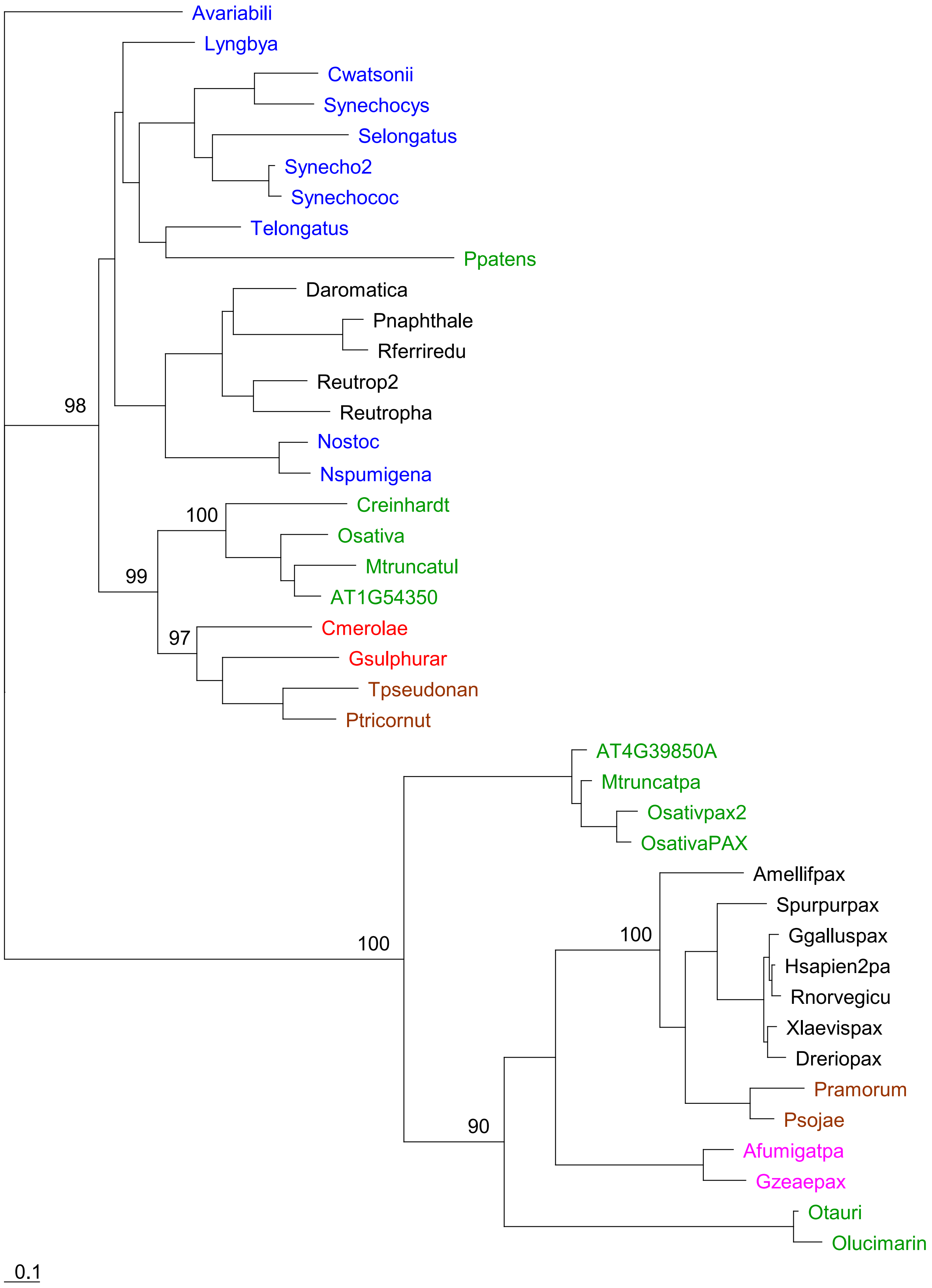

AT2G02590: Expressed Protein

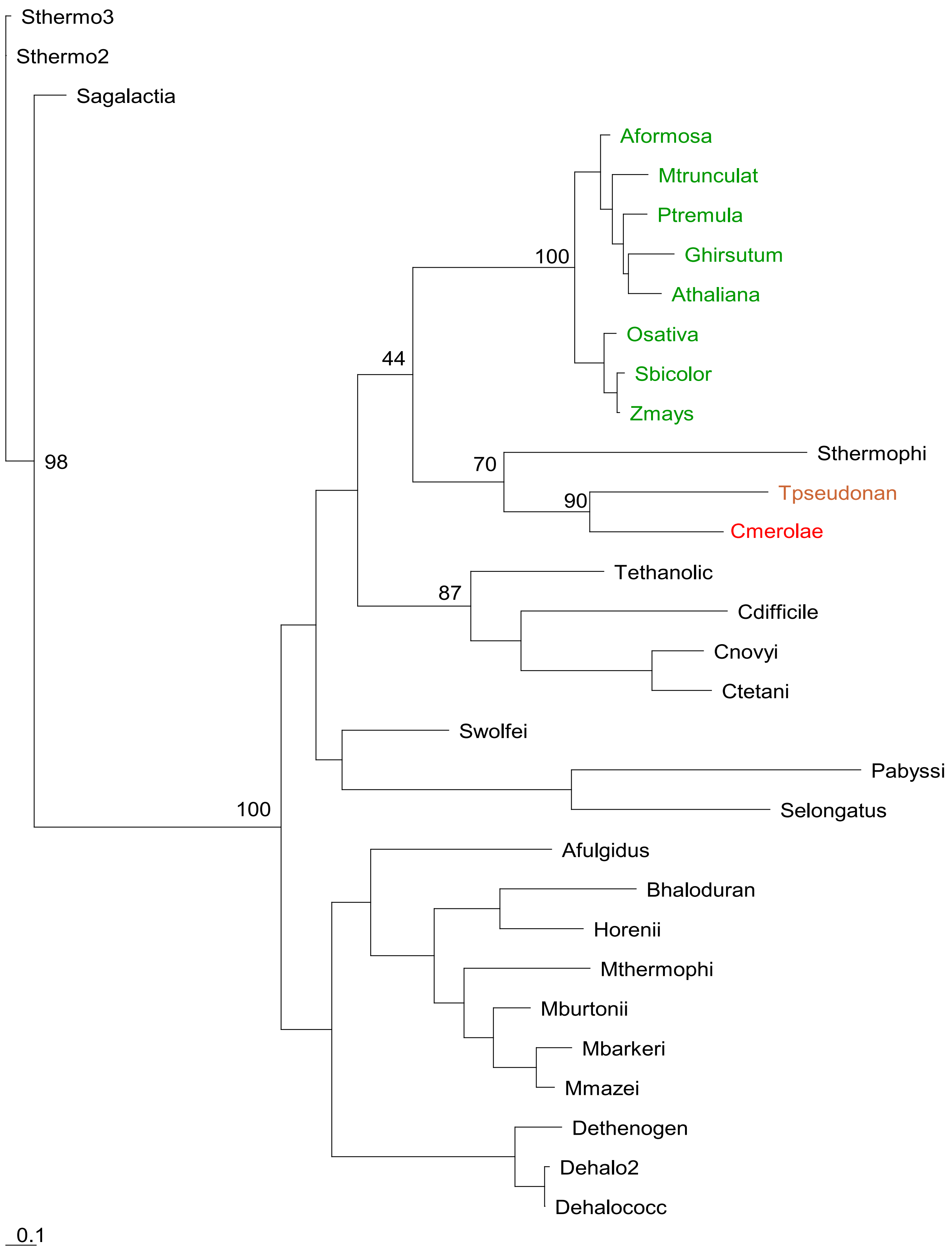

AT2G21340: Enhanced Disease Susceptibility Protein

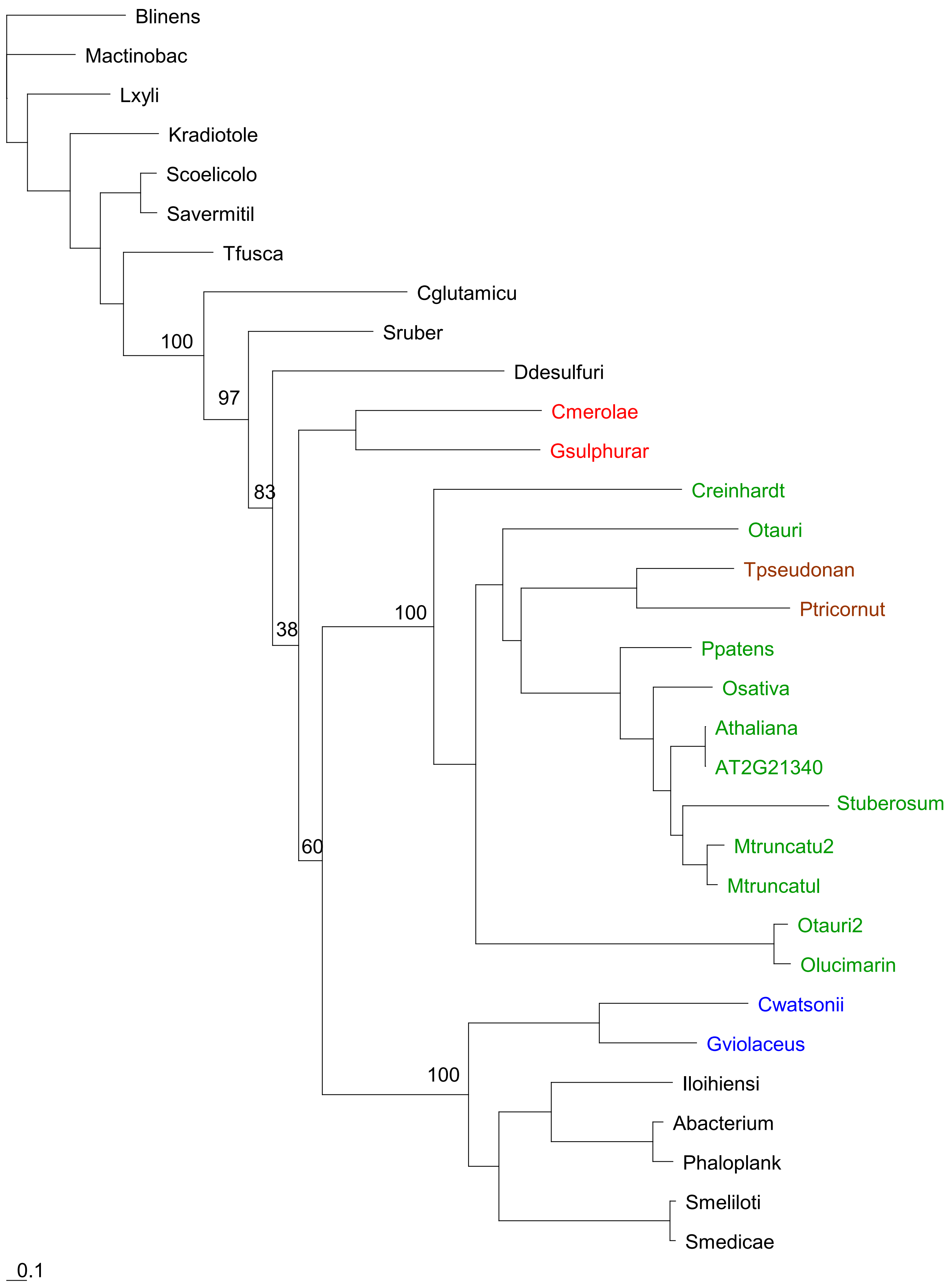

AT4G30580: ATS2

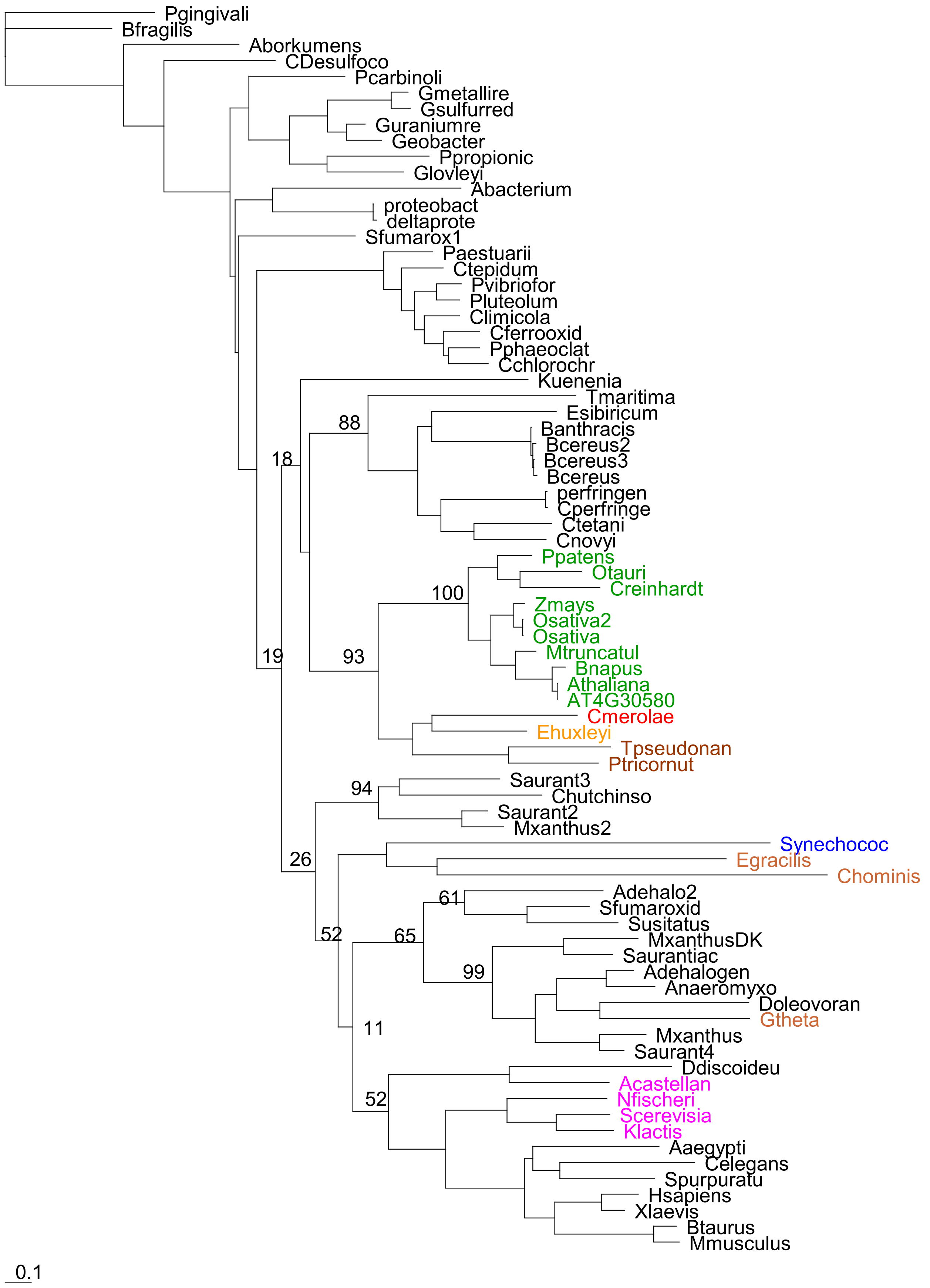

AT5G03555: Allantoin Family Permease

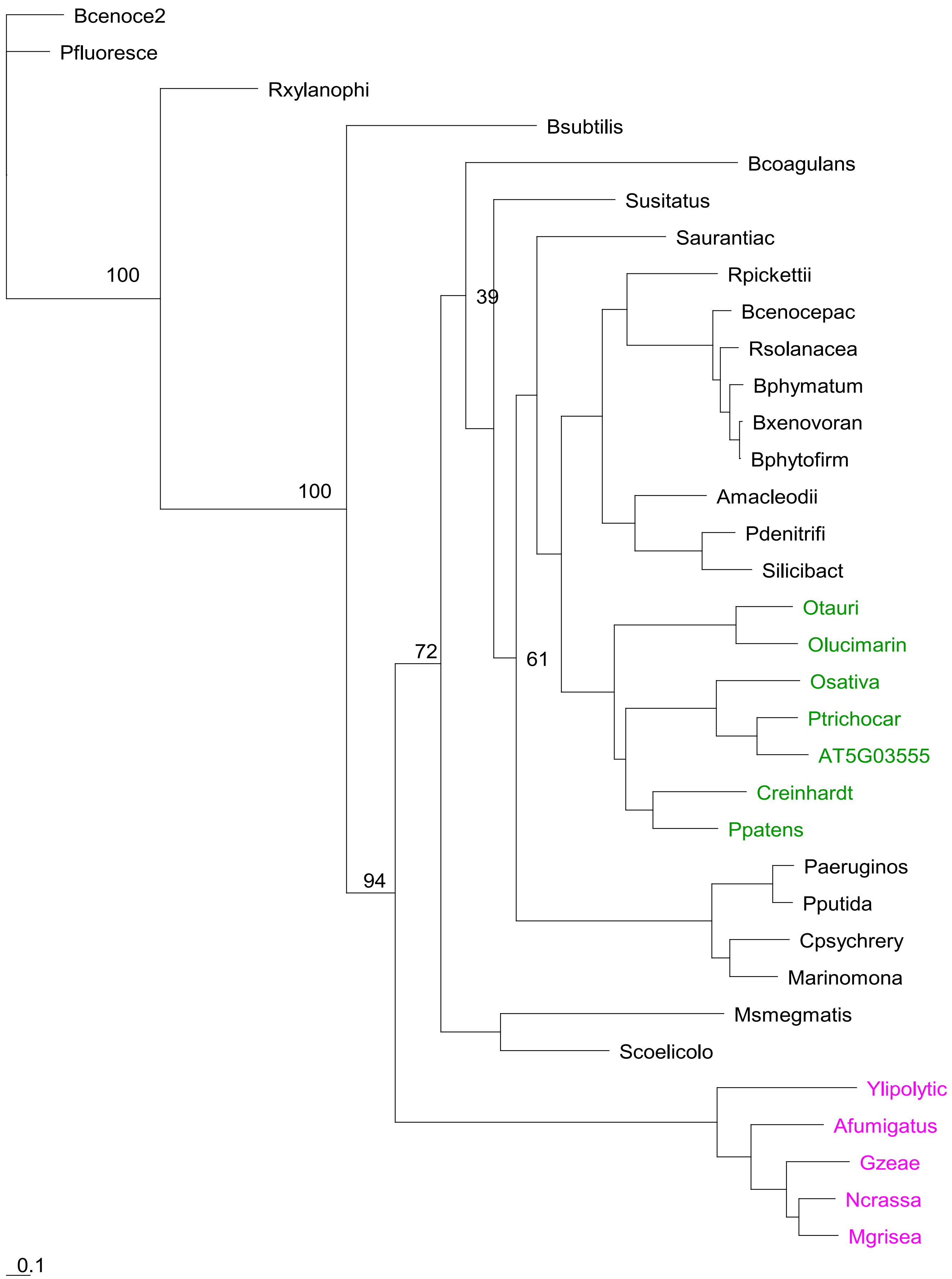

AT5G13720: Expressed Protein

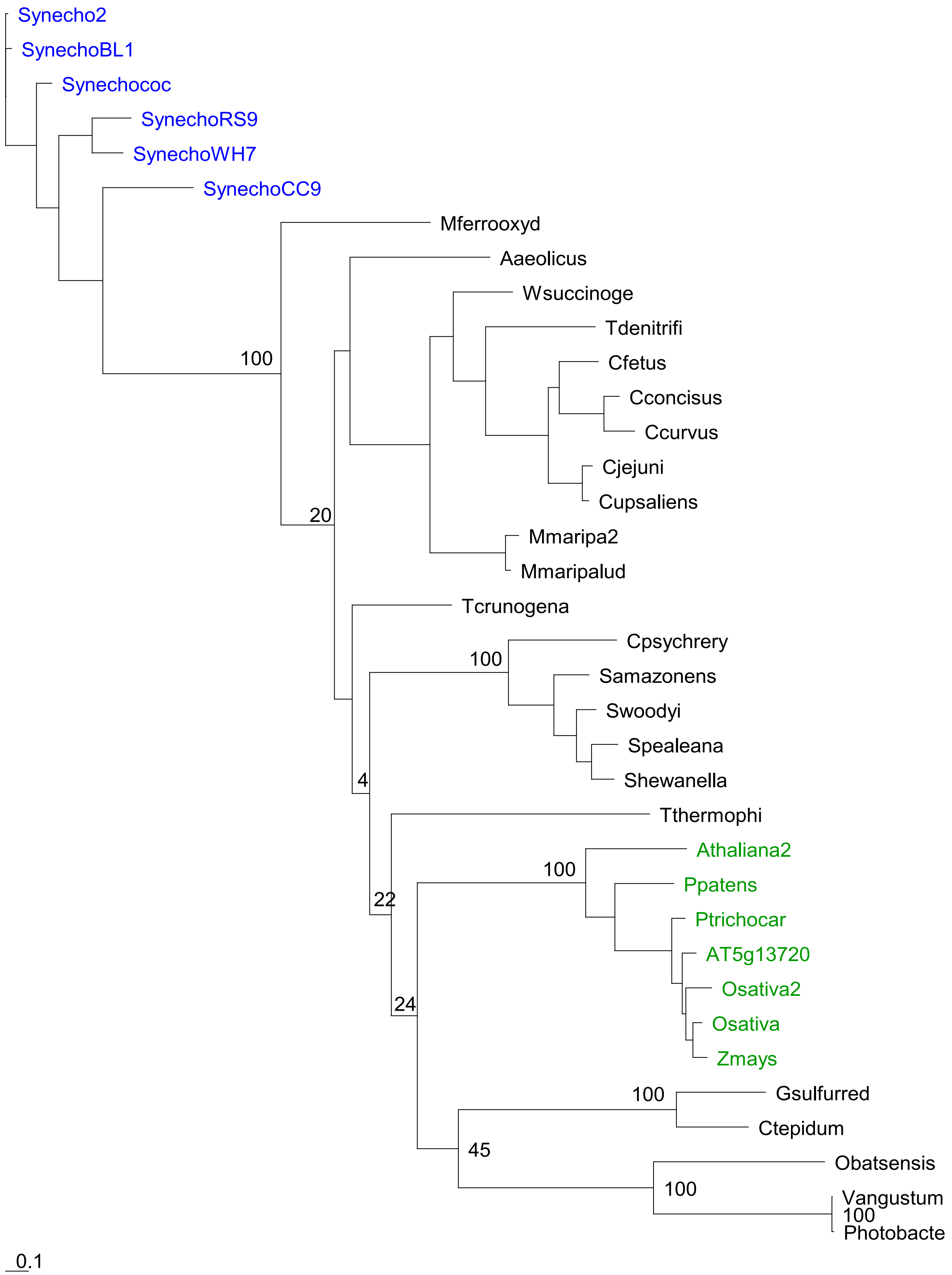

AT5G62720: Integral Membrane HPP Family Protein

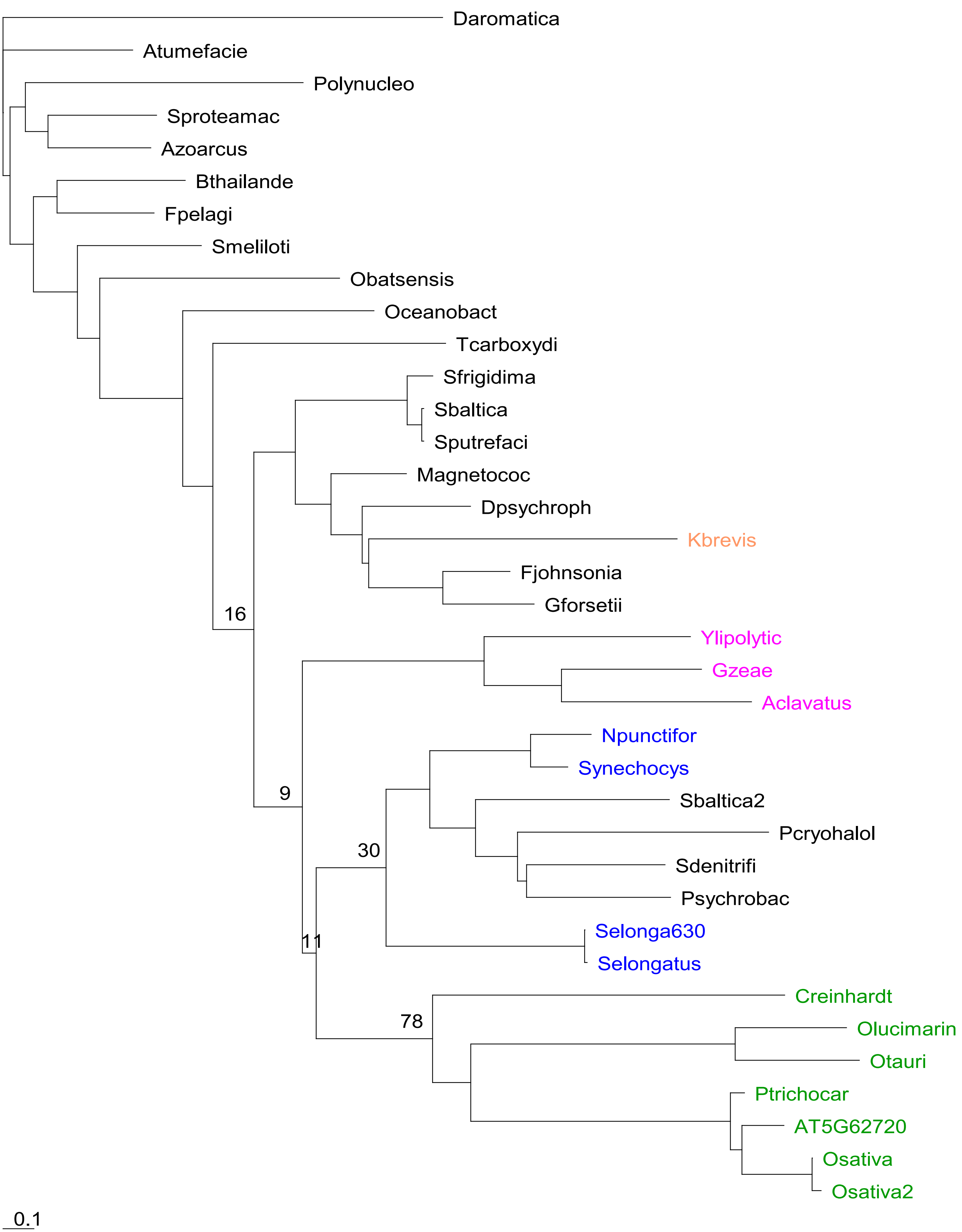

AT1G78560, AT2G26900, AT3G25410: Bile Acid:Sodium Symporter

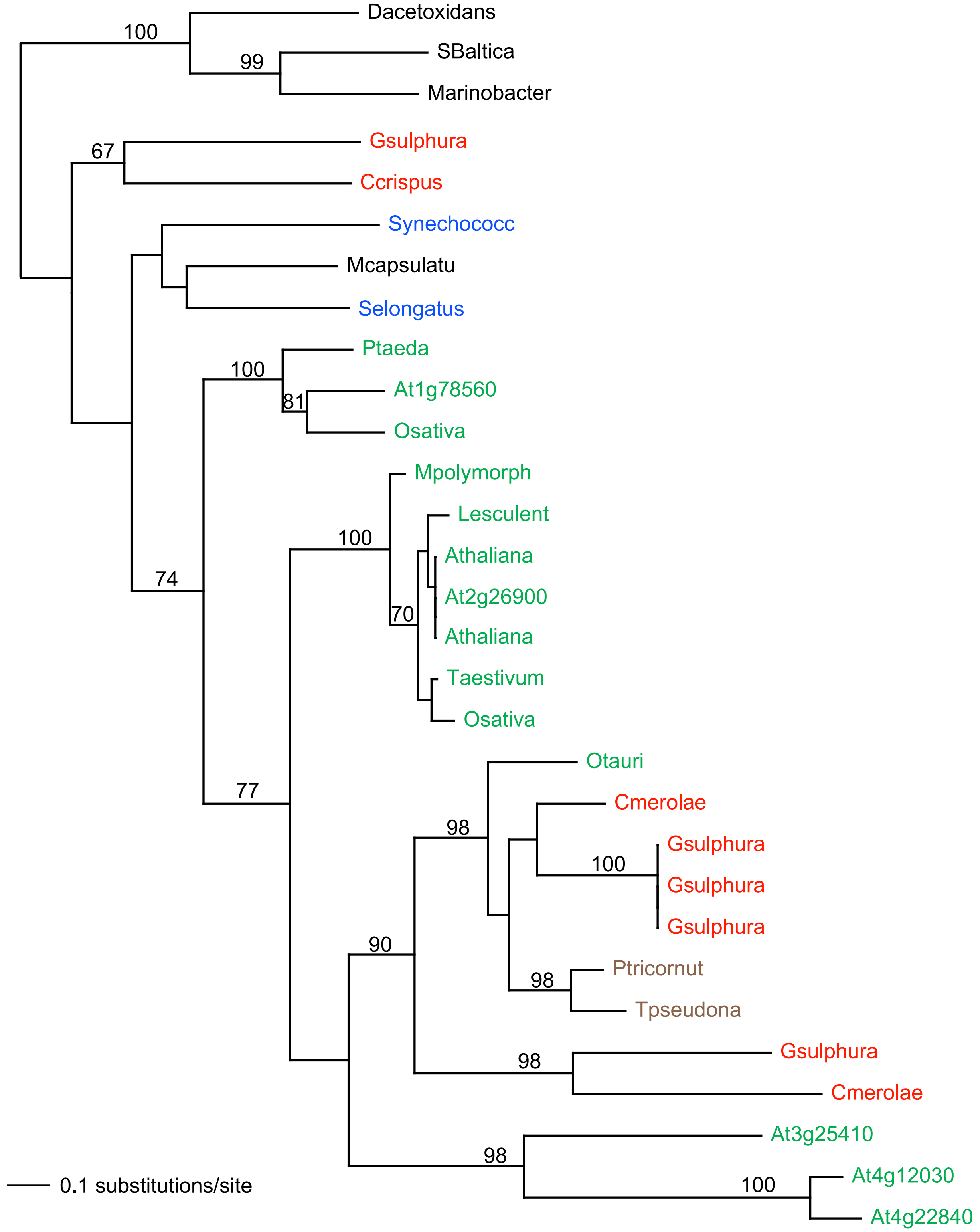

Supplementary Material:

Host Origin of Plastid Solute Transporters in the First Photosynthetic Eukaryotes

Heather M Tyra, Mark Linka, Andreas P.M. Weber, and Debashish Bhattacharya

Figure S5. Plastid targeted solute transporters of putative “Plantae-specific” origin in Plantae. Four unique genes fall in this category. These are PHYML trees with the numbers above the branches inferred from a PHYML bootstrap analysis. Only bootstrap values ≥ 60% are shown. Branch lengths are proportional to the number of substitutions per site (see scale bars). The different algae are shown in different text colors: red for red algae, green for green algae and land plants, magenta for glaucophytes, and brown for chromalveolates. The inclusion of chromalveolates within the Plantae is believed to reflect horizontal or endosymbiotic gene transfer events (e.g., Li et al. 2006).

At5g17520: MEX1

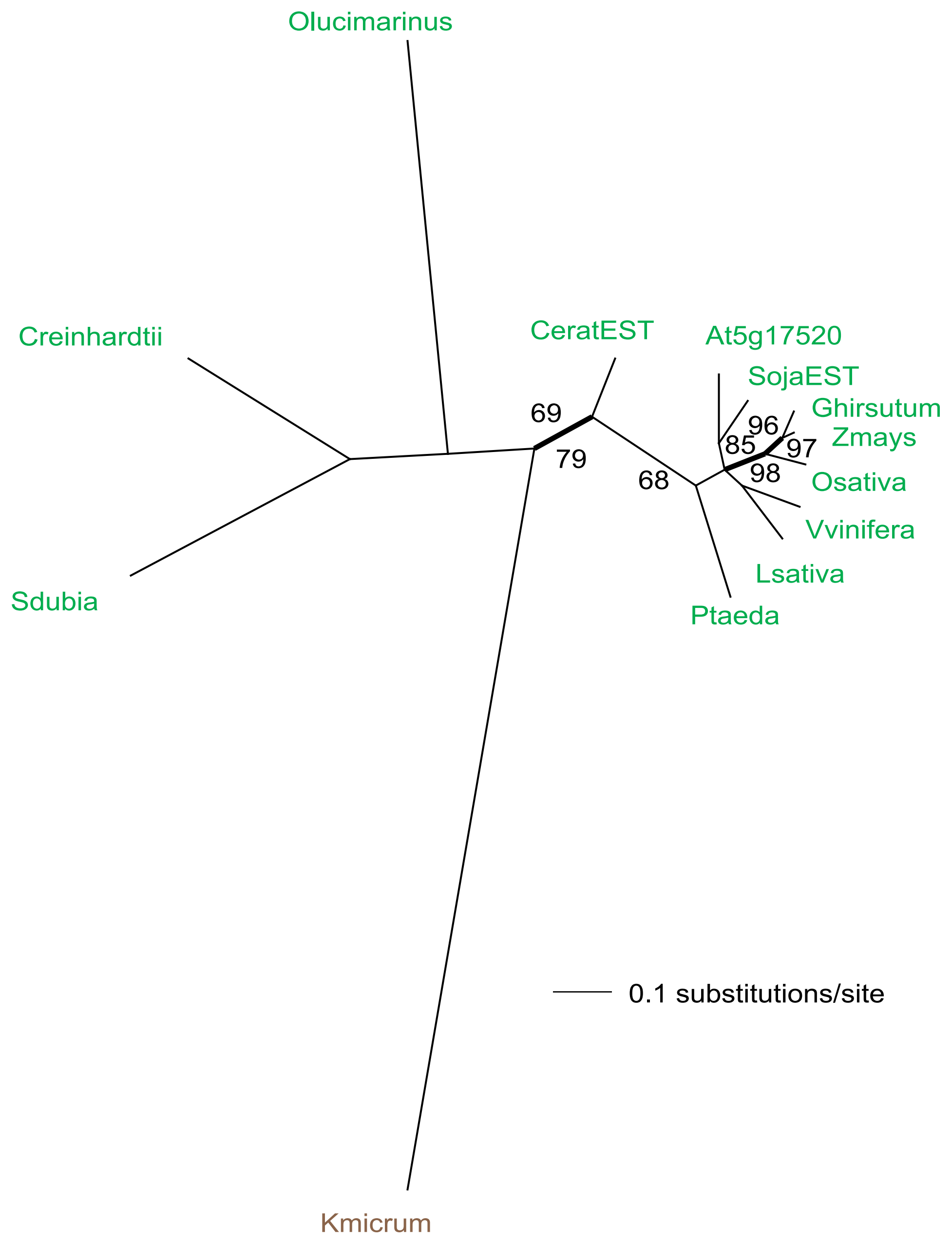

AT3G57280: Expressed Protein

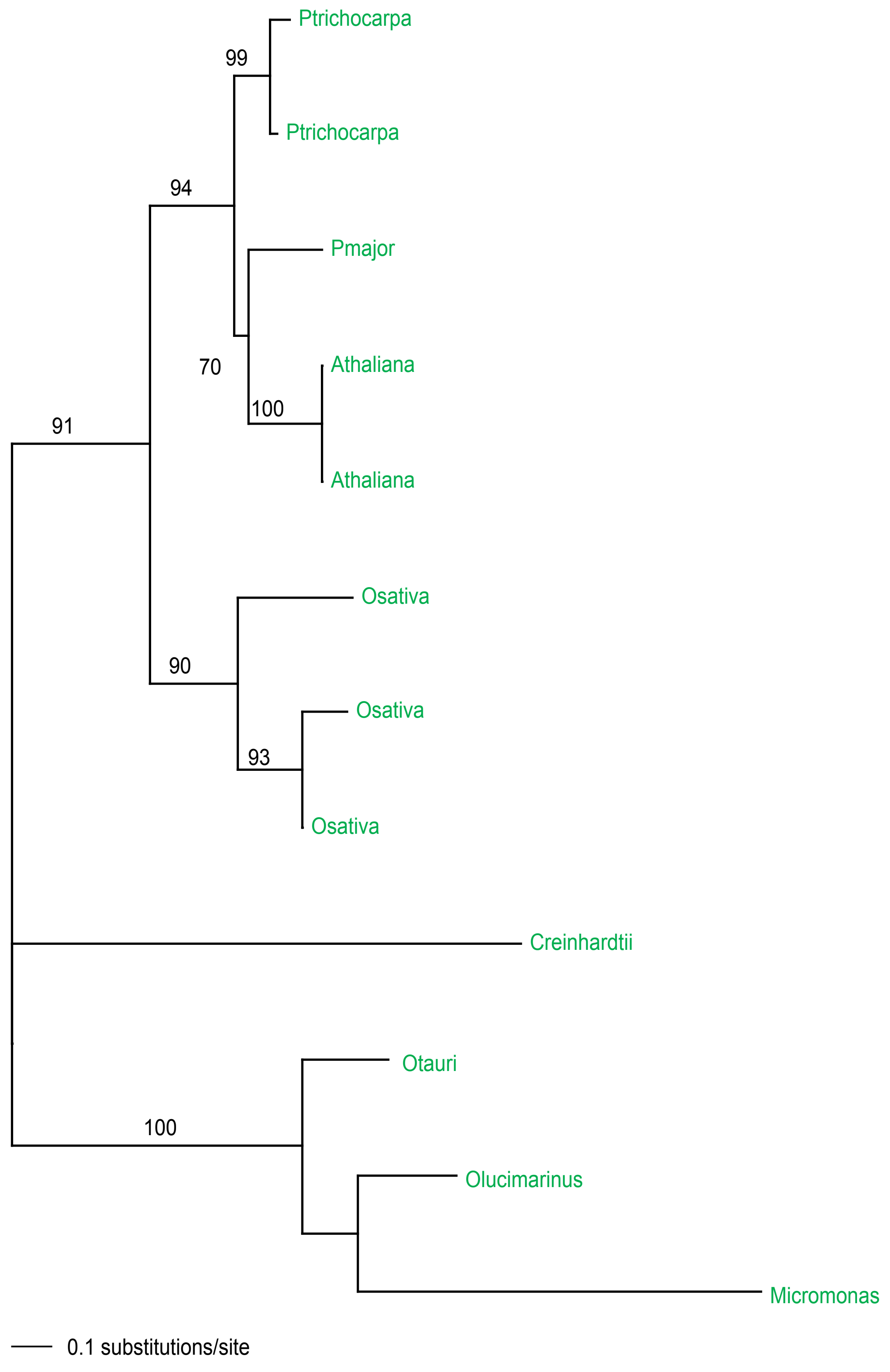

At5g24690: Expressed Protein

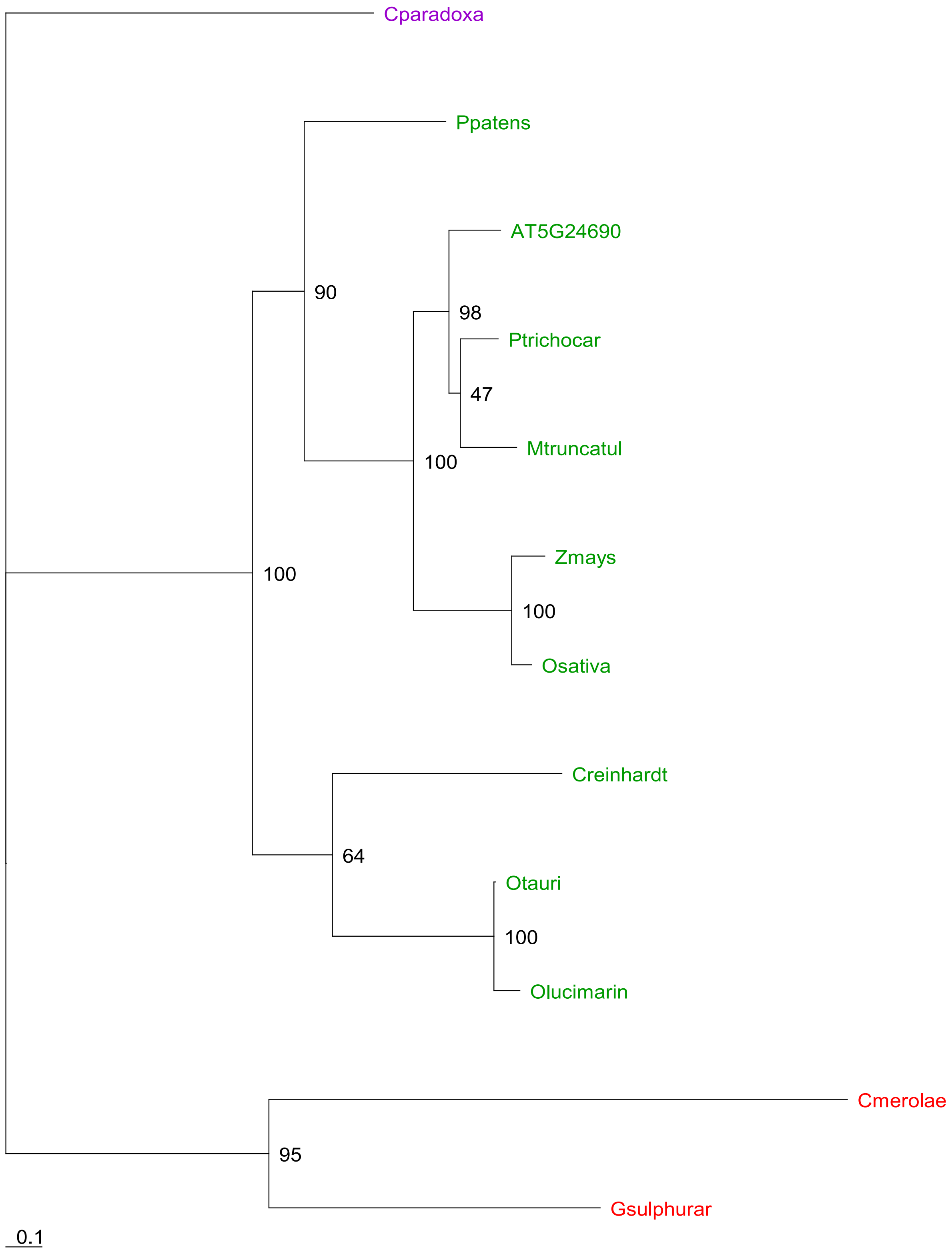

At2g38550: ATS2

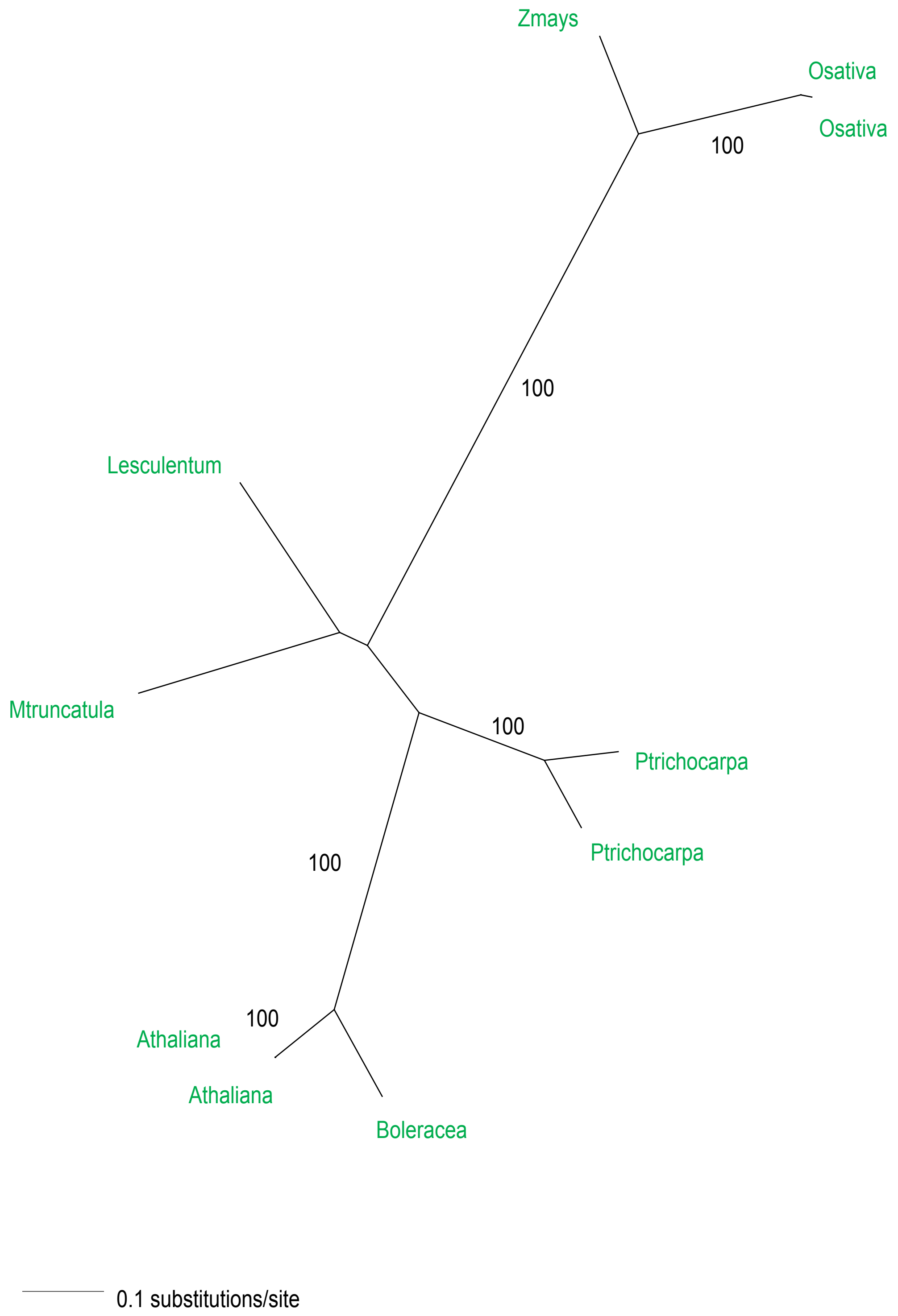

Supplement: Additional data file 1 — PHYML bootstrap trees of plastid targeted solute transporters of putative 'Host', 'Cyanobacterial', 'Chlamydia-like', 'Other', and 'Plantae-specific' origin found in our study. [file gb-2007-8-10-r212-S1.pdf]
